# Supplementary material for: Metagenomics of Neotropical Single-Stranded DNA Viruses in Tomato Cultivars with and without the Ty-1 Gene
Source: Viruses. 2020 Jul 28;12(8):819. doi: 10.3390/v12080819 (PMC7472167; doi:10.3390/v12080819)
Supplement: Supplementary file 1 [file viruses-12-00819-s001.pdf]

viruses

MDPI

Supplementary Materials: Metagenomics of Neotropical Single-Stranded DNA Viruses in Tomato Cultivars with and without the *Ty*-1 Gene

Table S1. Geographic distribution of 21 species of begomovirus reported naturally infecting tomatoes in Brazil.

Species	Acronym	Geographic Distribution
Chino del tomate Amazonas virus	CdTAV	AM
Tomato bright yellow mosaic virus	ToBYMV	BA
Tomato bright yellow mottle virus	ToBYMoV	TO
Tomato chlorotic mottle virus	ToCMoV	BA, MG, DF, ES, PE & RJ
Tomato common mosaic virus	ToCmMV	RJ, MG & ES
Tomato golden leaf distortion virus	ToGLDV	TO
Tomato golden leaf spot virus	ToGLSV	TO
Tomato golden mosaic virus	TGMV	BA, DF, MG, PR, RN, RJ & SP
Tomato golden vein virus	ToGVV	GO, DF & MG
Tomato interveinal chlorosis virus	ToICV	PE
Tomato leaf distortion virus	ToLDV	MG & RJ
Tomato mild mosaic virus	ToMMV	MG & RJ
Tomato mottle leaf curl virus	ToMoLCV	MG, GO, DF & PE
Tomato rugose mosaic virus	ToRMV	MG, GO, DF, SP, PR & BA
Tomato rugose yellow leaf curl virus	TRYLCV	RS
Tomato severe rugose virus	ToSRV	DF, GO, MG, RJ, SP, PE & RS
Tomato yellow spot virus	ToYVSV	GO & MG
Tomato yellow vein streak virus	ToYVSV	DF, GO, MG, RS, RJ & SP
Tomato leaf curl purple vein virus	ToLCPVV	PI
Tomato interveinal chlorosis virus-2	ToICV2	GO
Tomato chlorotic leaf curl virus	ToCLCV	PA

Amazonas (AM); Bahia (BA); Tocantins (TO); Minas Gerais (MG); Distrito Federal (DF); Espírito (ES); Pernambuco (PE); Paraná (PR); Rio Grande do Norte (RN); Rio Grande do Sul (RS); Rio de Janeiro (RJ); São Paulo (SP) and Pará (PA). References: Genbank. https://www.ncbi.nlm.nih.gov/genbank/ and Reis, L. N. A., Metagenomic analysis of the begomovirus diversity in tomatoes in Central Brazil and impact of the *Ty*-1 tolerance gene on viral evolutionary dynamics. 2020.

Sample sequences with *Ty*-1 NGS with codes

>B_GO_247_LR_LVV_27_-_MT215002 Tomato severe rugose virus (ToSRV) - DNA B

ACCGGATGGCCGCGCGATTTTTCTTATCTGCTACGTGGCGAAATCGTGTACGTTGCCTCGCG
CTTTCCATTTTAATTGAGCGCTTTTTTGAAGTCCGCGAAATGAGTTAATTGTCTTTTTGAAAT
CCGCTTTTGTGAATCACCTTTAATTTGAATTAAAGGTTGGATAGTTCATATTGATCAATCATT
TCGCTGGTTTATTTCCTGTCGTGGTGTAATTACAACCGTTCGTTAAAAATATAAGAAATTTAC
GACGTGGACTGTCTAAATTTCATCTACATAGTTAATTTGACAAATGAATGCATATTTAAACT
CTGCTTTCGTGTGGGTTTACACCACGTCTATACATATTGTCCAGGTTATTTTGTATAAGTATA
ATTTTTATTTTGTCTTATCTTATTATATGAACATGTATCCCATTAAGTATAGACGTGGAATGTT
GTTTAATCATCGACGAGGTTACTCATCTAATCCCGTATTTAAGCGTTTACACGGAGCGAAAC
GAAGTGATTTCAAGCGTCGTTCGAGTAATCAGATTAAGAGCATGGATGAGACTAAAATGTC
TGTTCAGCGGATTCATGAGAACCAGTTTGGCCCTGAATTTGTAATTGGCCACAATTCTGCCA
TATCCACATTCATTACATTCCCTACTCTTTGTAAGACTGTCCCGAACCGTTGTAGGTCATACA

TAAAGTTAAGACGACTACGTTTTAAAGGAACAATCAAGATTGACCGTGTTCATGCTGAGGT
GAATATGGACGGTACAAGTCCAATGATTGAAGGAGTCTTCTCTCTGGTTGTAGTCGTTGATC
GCAAACCTCATTTGGGCTCATCTGGAACTCTGCATTCTTTTGATGAGATATTTGGTGCAAGG
ATTCATAGCCATGGTAATTTGGCAATAGTATCCTCTCTGAAAGAGCGTTTTTACATACGTCA
CGTTTGGAAGAAAGTAATATCTGTTGAGAAGGATACAACCATGGTTGATGTTGAAGGAAGT
ACTATTTTATCTAACAGGCGTTTTAATTGTGGTCATCCCTTTAAGGATATTGACCGTGAATCA
TGTAATGGTGTTTATGCAAACATAAGCAAGAACGCCCTGTTAGTTTATTACTGTTGGATGTC
TGATAATGTGTCTAAGGCATCGACATTTGTATCATTTGACCTTGATTATGTTGGCTAAATAGT
AAGTGTAATACGTGATTAGTAATAATATTTAACTCGCTGTAATAATAAATACAAGTACTTGT
TCAGTAATAATGTTCAACTCGCAGTAATAACAAAAAAATGTGTTTAATAATATAGAATTTA
TTTTAAAGATTTTGGCTGTGAAGGAGTACAGTTGCTGTTTATACATTCATGGGCCGCTGCTTT
AACAAGTTCGTTTAATTGGGCCATTGACAGTGTTATGTTCGATTGGGCCCTTTGAGCACCAA
TTACGGACGCAGAATCACCTGGGTCTAATGTGCCTGTACCCAGTCTATGTAATTGTCTGTAT
GGATGCATCGCGTTCTCTATATCTGAGCCCGCCTCTGATGGGCTTAAGCCTACAGTACTTCT
AACAGCCCATGACTCTCCGGGCATTATTTCAAGTGGGTTGTGAAGGCCCAATCTTGATATTG
AGGCGGATCTAATCATCTTCCTTTCCCATTTGCCATAGCCTACGTGACTGAAATCAATGTCC
TTATCTGTAAATTGTTTTGAAAGGATCTTCACCGTTGGGGCACGGAAAGGTATATCAACCGA
ATGTTTAGCCGTCGAGAGTTTCAGTCTCCCTTTGAATTTAGCGAAGTGGGTACTCTGGTGAA
CGTTCGTGTCACATACTCTGTAGTACAATTTCCATGGAATTGGGTCCTTAAGGGAGAAGAAC
GACGACGAAAAGTAGTGAAGATCTATGTTACATCTGATAGGAAAGGTCCACGACGCCTGT
AGTGATTCATTGTCCGTCATTCTTCTGTCATGAATTTCGACTATAACTGAACCTACGGCGTTA
ATTGGTACCTGTTGTCTGTATTCTATTACGCAATGGTCGATTTTCATACAGCTACGACTAAGC
CTTGCGCTTAATTGAGACGCCGTTGAAGGGAATTGAAGCATTATCTCGGTTAGATCATGGG
AAAGCTGATATTCATCACGCTTAGATTCTATATAATTAAATGCGTTGGGAGGATTCACAAGC
TGAGATTCCATTATTGAAAATAGGGAGCGCAGCGACAATGTTTGAGGAAAGTTAATAAGG
GAAGATGATAATATTTTCGTCAACTGAATATATGACAAGAAAATTGTCTGTTGATCCAATTG
GGAAATTGGAGATGATAATAAGTAAAAAACGAGGAATCAGGAGACGACGAATTGAGAGG
AGATATTGTCTATTTTAGGTGTAATTGATAAATACCCCTCTACTTGCTCTTTAAATAGAATTT
TCAAGGCAATGGCATATTTGTAAATATGACCCTTACTACCAAATGGTAGCTGCTCTAAAACT
CATATGAATTGGTAGTTATGGTAGCTCTTATATAGTAGAAGTTCTCTTTAAGGAGATTGCTA
CACGTGGCGGCCATCCGTTATAATATT

>A_GO_247_LR_LVV_27_-_MT215001 Tomato severe rugose virus (ToSRV) - DNA A

ACCGGATGGCCGCGCGATTTTTCACCCCTTTAGTTTCAATTAAAGTAAAGTGATTGTCTGTG
GCCCAATCATATTGGGCCTGTCGAGCTTAGATATTTGTAACAACTTAAGGCCCAAGTTGTTA
AACGGCTATAAATTGAACATACACTTTACTTTTGCTTTAATTCAAAATGCCTAAGCGTGATG
CCCCATGGCGTTTAACGGCGGGAACTTCAAAGGTTTCCCGCTCTGTCAATTATTCTCCCCGT
GCAGGATATGGACCCAAATATAACAAGGCCGCTGAGTGGGTGAACAGGCCCATGTACAGG
AAGCCCAGGATCTACCGTACTTTGAGAGGCCCAGATGTTCCTAGAGGCTGTGAAGGGCCTT
GTAAGGTTCAGTCTTACGAGTCTCGTCATGATGTTTCCCATATCGGGAAGGTGATTTGTGTG
TCTGACGTTACACGTGGTAACGGTATTACTCACCGTGTTGGTAAGCGTTTCTGCGTGAAGTC
TGTATATATTTTAGGGAAGGTATGGATGGACGAGACCATCAAGTTGAAGAATCACACAAAT
AGTGTGATGTTCTGGTTGGTTAGAGATCGGAGACCTTATTCGTCACCTATGGATTTTGGCCA
GGTGTTCAACATGTTTGACAACGAGCCTAGCACTGCAACTGTTAAGAACGATCTTCGGGAT
CGTTTTCAGGTCATGCACAAGTTTTATGCCAAGGTTACTGGTGGACAGTATGCCAGTAATGA
GCAGGCATTAGTGAAGCGCTTTTGGAAGGTCAACAACAACGTAGTCTACAACCATCAGGA

GGCAGGGAAATACGAGAATCATACTGAGAACGCCTTGCTATTGTATATGGCATGTACTCAT
GCCTCTAACCCCGTGTATGCTACATTGAAAATTCGGATCTATTTTTATGATTCGATTACTAAT
TAATAAAATTTAAATTTTATTGAATGATTTTCGAGTACAGCATTTACATATGATTTGTCTGTT
GCGAAACGAACAGCTCTGATTACATTATTAATGGAAATAACGCCTAATCGATCTAGATACA
ATAAGACTAAATATTTAAATCTAGTTAAATATGTCGTCCCAGAAGCTGTCAGTGAAGTCGT
CCATATCTGGAAGTTGAGGAAGCTCTTGTGGAGATGCAAYGCTCTCCTCAGGTTGTGGTTGA
ACCGTATTTGGACGTGGTAGACTCTGCTCGCGGTGTACATTGGATCCTCCACTCTGTTTATCT
TGAAATAGAGGGGATTTGATATCTCCCAAATATAGACGCCATTCTCTGCCTGACGTGCAGT
GATGAGTTCCCCTGTGCGTGAATCCATGTCCTCTGCAGTCGATGTGTACGTAAATAGAGCAC
CCGCACTCTATATCAATTCGTCGTCTCCTGATTCCTCGTTTTTTAGCAGCTCTGTGTTGTACCT
TGATAGAGGGGGGTGTTAAGGAAGACGAATTTCGCATTGTGCTTTGTCCAATTATTTAGACT
TGCATTTTCTTCTTTGTCGAGGAAACATTTATAGCTGGCCCCCTCGCCAGGATTGCAAAGCA
CGATGCATGGGATACCACCTTTAATTTGAACTGGCTTTCCGTATTTGCAGTTTGATTGCCAAT
CCTTTTGGGCCCCAAGCAATTCTTTCCAGTGCTTTAACTTTAGATAGTGCGGTGCGATGTCAT
CAATGACGTTATATTCAACATGATTTGAATAAACCCTAGGATTGAAATCTAGGTGTCCACTC
AAATAATTATGGGCCCCTAATGCACGTGCCCACATCGTCTTTCCCGTTCGAGAATCACCTTC
AATGATGATACTAATAGGTCGTTCCGGCCGCGCAGCGGCACCTCTCCCAAAATAGTCATCT
GCCCAATCTTGCATCTCTCCAGGCACGCTAGTGAAAGAGGAGAGGGGAAACGTAGGGGCC
CATGGCTCCGGAGCCCTTGTAAATATCCTATCTAAATTACTATTTAGATTGTGAAACTGAAA
TAAGAACTTTTCAGGCAGCTTCTCACGGATTATCTGCAAGGCGACGTCTTTGGAAGGTGCGT
TCAAGGCTTCTGCGGCAGCGTCGTTAGCTGTCTGGCAACCGCCTCTAGCACTTCTTCCGTCG
ATTTGGAATTCCCCCCACTCGATAGTATCTCCGTCCTTGTCGACATAGGATTTGACGTCGGA
CGATGACTTAGCTCTCTGAATGTTCGGATGGAAATGTGTTGACCTTGTTGGGGAGACCAGGT
CGAAGAATCGTTGATTTTGGCAGCAGTAGTTGCCCTCAAATTGAAGAAGCACGTGGAGATG
AGGCTCCCCATTTTCATGGAGCTCTCTGCAAACCTTGATGAACTTCTTATTTGTAGGAGTGTT
TAGGGTTTTTAATTGGGAAAGTGCTTCTTCTTTCGATAATGAGCATTTGGGATATGTGAGGA
AATAATTCTTCGCTTTTATTTGAAAGCGCTTTGGAGATGATGGCATATTTGTAAATATGACC
CTTACTACCAATTGGTAGCTGCTCTAAAACTCATATGAATTGGTAGTTATGGTAGCTCTTAT
ATAGTAGAAGTTCCTTTAAGGAGATTGCTACACGTGGCGGCCATCCGTTATAATATT

>B_DF_227_LR_LVV_26_-_MT215007 Tomato rugose mosaic virus (ToRMV) - DNA B

ACCGGATGGCCGCGCGATTTTTCTTATCTGCTACGTGGCGAAATCGTGTACGTTGCCTCGCG
CTTTCCATTTTAATTGAGCGCTTTTTTGAAGTCCGCGAAATGAGTTAATTGTCTTTTTGAAAT
CCGCTTTTGTGAATCACCTTTAATTTGAATTAAAGGTTGGATAGTTCATATTGATCAATCATT
TCGCTGGTTTATTTCCTGTCGTGGTGTAATTACAACCGTTCGTTAAAAATATAAGAAATTTAC
GACGTGGACTGTCTAAATTTCATCTACATAGTTAATTTGACAAATGAATGCATATTTAAACT
CTGCTTTCGTGTGGGTTTACACCACGTCTATACATATTGTCCAGGTTATTTTGTATAAGTATA
ATTTTTATTTTGTCTTATCTTATTATATGAACATGTATCCCATTAAGTATAGACGTGGAATGTT
GTTTAATCATCGACGAGGTTACTCATCTAATCCCGTATTTAAGCGTTTACACGGAGCGAAAC
GAAGTGATTTCAAGCGTCGTTCGAGTAATCAGATTAAGAGCATGGATGAGACTAAAATGTC
TGTTCAGCGGATTCATGAGAACCAGTTTGGCCCTGAATTTGTAATTGGCCACAATTCTGCCA
TATCCACATTCATTACATTCCCTACTCTTTGTAAGACTGTCCCGAACCGTTGTAGGTCATACA
TAAAGTTAAGACGACTACGTTTTAAAGGAACAATCAAGATTGACCGTGTTCATGCTGAGGT
GAATATGGACGGTACAAGTCCAATGATTGAAGGAGTCTTCTCTCTGGTTGTAGTCGTTGATC

GCAAACCTCATTTGGGCTCATCTGGAACTCTGCATTCTTTTGATGAGATATTTGGTGCAAGG
ATTCATAGCCATGGTAATTTGGCAATAGTATCCTCTCTGAAAGAGCGTTTTTACATACGTCA
CGTTTGGAAGAAAGTAATATCTGTTGAGAAGGATACAACCATGGTTGATGTTGAAGGAAGT
ACTATTTTATCTAACAGGCGTTTTAATTGTGGTCATCCCTTTAAGGATATTGACCGTGAATCA
TGTAATGGTGTTTATGCAAACATAAGCAAGAACGCCCTGTTAGTTTATTACTGTTGGATGTC
TGATAATGTGTCTAAGGCATCGACATTTGTATCATTTGACCTTGATTATGTTGGCTAAATAGT
AAGTGTAATACGTGATTAGTAATAATATTTAACTCGCTGTAATAATAAATACAAGTACTTGT
TCAGTAATAATGTTCAACTCGCAGTAATAACAAAAAAATGTGTTTAATAATATAGAATTTA
TTTTAAAGATTTTGGCTGTGAAGGAGTACAGTTGCTGTTTATACATTCATGGGCCGCTGCTTT
AACAAGTTCGTTTAATTGGGCCATTGACAGTGTTATGTTCGATTGGGCCCTTTGAGCACCAA
TTACGGACGCAGAATCACCTGGGTCTAATGTGCCTGTACCCAGTCTATGTAATTGTCTGTAT
GGATGCATCGCGTTCTCTATATCTGAGCCCGCCTCTGATGGGCTTAAGCCTACAGTACTTCT
AACAGCCCATGACTCTCCGGGCATTATTTCAAGTGGGTTGTGAAGGCCCAATCTTGATATTG
AGGCGGATCTAATCATCTTCCTTTCCCATTTGCCATAGCCTACGTGACTGAAATCAATGTCC
TTATCTGTAAATTGTTTTGAAAGGATCTTCACCGTTGGGGCACGGAAAGGTATATCAACCGA
ATGTTTAGCCGTCGAGAGTTTCAGTCTCCCTTTGAATTTAGCGAAGTGGGTACTCTGGTGAA
CGTTCGTGTCACATACTCTGTAGTACAATTTCCATGGAATTGGGTCCTTAAGGGAGAAGAAC
GACGACGAAAAGTAGTGAAGATCTATGTTACATCTGATAGGAAAGGTCCACGACGCCTGT
AGTGATTCATTGTCCGTCATTCTTCTGTCATGAATTTCGACTATAACTGAACCTACGGCGTTA
ATTGGTACCTGTTGTCTGTATTCTATTACGCAATGGTCGATTTTCATACAGCTACGACTAAGC
CTTGCGCTTAATTGAGACGCCGTTGAAGGGAATTGAAGCATTATCTCGGTTAGATCATGGG
AAAGCTGATATTCATCACGCTTAGATTCTATATAATTAAATGCGTTGGGAGGATTCACAAGC
TGAGATTCCATTATTGAAAATAGGGAGCGCAGCGACAATGTTTGAGGAAAGTTAATAAGG
GAAGATGATAATATTTTCGTCAACTGAATATATGACAAGAAAATTGTCTGTTGATCCAATTG
GGAAATTGGAGATGATAATAAGTAAAAAACGAGGAATCAGGAGACGACGAATTGAGAGG
AGATATTGTCTATTTTAGGTGTAATTGATAAATACCCCTCTACTTGCTCTTTAAATAGAATTT
TCAAGGCAATGGCATATTTGTAAATATGACCCTTACTACCAAATGGTAGCTGCTCTAAAACT
CATATGAATTGGTAGTTATGGTAGCTCTTATATAGTAGAAGTTCTCTTTAAGGAGATTGCTA
CACGTGGCGGCCATCCGTTATAATATT

>A_DF_227_LR_LVV_26_-_MT215006 Tomato rugose mosaic virus (ToRMV) - DNA A

ACCGGATGGCCGCGCGATTTTTTTAAGGCCCATTTATTTTGGGCCTCTCTTTTGGGCCTTCAC
CTTTAATTTTAATGAAAGTAATTTTGACCCAATCATGATGCATTTGACGAGCTAAGATATTT
AGAACTACTTAACGCCCAAGTTGCATAACGGCTATAAATTAAACGTACATATGTCGATAGC
TTTAATTCAAAATGCCTAAGCGCGATGCCCCATGGCGCCACATGGCTAGTACATCTAAGAT
TAGCCGGGGCGTTAATAATTCTCCACGTGCAGGAGTTGGGCCACGGTCCAACAAGGCCACT
GACTGGGTAAACAGGCCCATGTACAGGAAGCCCAAGATATATCGGATGTATAGAACCCCC
GATGTTCCAAGGGGTTGTGAAGGCCCATGTAAAGTGCAGTCGTTTGAACAGCGTCACGATA
TCTCCCATACTGGTAAGGTGATATGCGTCTCCGATGTGACACGTGGTAACGGTATTACACAC
CGTGTGGGTAAGCGTTTCTGTGTTAAGTCTGTGTATATTCTGGGTAAGATATGGATGGATGA
GAACATCAAGTTGAAGAACCACACGAACAGTGCCATGTTCTGGTTGGTCAGGGACCGTAG
ACCTTATGGTACCCCTATGGACTTTGGCCAGGTGTTTAACATGTTCGACAACGAGCCTAGCA
CCGCCACTGTGAAAAACGATCTCCGTGATCGTTTTCAAGTCATGCATAAGTTCTATGCTAAG
GTCACAGGTGGACAATACGCAAGCAACGAGCAGGCGCTTGTCAAGCGGTTCTGGAAGGTC

AACAATCATGTCGTCTACAACCATCAAGAAGCTGGAAAATACGAGAATCATACGGAGAAC
GCCCTATTATTGTATATGGCATGTACTCATGCCTCAAACCCCGTGTATGCAACTTTAAAAAT
TCGGATCTATTTTTATGATTCGATAACAAATTAATAAATTTTGAATTTTATTGAATGATTCTC
GAGTACAGCATTTACATATCGTCTGTCTGTTGCGAAGCGAACAGCTCTAATTACATTATTAA
CTGAAATAACGCCTAGCTGATCTATGTACAACATAAGTAAATATTTAAATCTATTTAAATAT
GTCATCCCAGAAGCTCTCAGGGATGTCGTCCAGATTTGGAAGTTGAGATAAGCCTTGTGGA
GACCCAGTACTCTCCTCAGGTTGTGGTTGAACCGTATTTGGACGTGGTAGACTCTGGTCCTC
GTGTATAGTATGTCCTCGACGTTGTACATCTTGAAATAAAGGGGATTTGATATTTCCCAAGT
ATACACGCCATTCTCTGCCTGATGTGCAGTGATGAGTTCCCCTGTGCGTGAATCCATGTCCT
GCGCAATCTATGTGTACGAAAATGGAGCAGCCGCACTGTAAATCAATTCGTCTTCTTCGTAT
AGCTCTCCTCTTAGCAGCCCGATGTTGAACTTTGATAGAGGGGGGAGTTGAGGAAGATGAA
TTGTGCATTGTGCTTTGTCCAACTACTTAATGCTGAGTTCTCCTCTTTGTCGAGGAAACATTT
ATAGCTGGCCCCCTCGCCAGGATTGCAAAGCACGATGCATGGGATACCACCTTTAATTTGA
ACTGGCTTTCCGTATTTGCAGTTTGATTGCCAATCCTTTTGGGCCCCAAGCAATTCTTTCCAG
TGCTTTAACTTTAGATAGTGCGGTGCGATGTCATCAATGACGTTATATTCAACATGATTTGA
ATAAACCCTAGGATTGAAATCTAGGTGTCCACTCAAATAATTATGGGCCCCTAATGCACGT
GCCCACATCGTCTTTCCCGTTCGAGAATCACCTTCAATGATGATACTAATAGGTCGTTCCGG
CCGCGCAGCGGCACCTCTCCCAAAATAGTCATCTGCCCAAGCTTGCATCTCTCTCGGTACGC
TAGTGAAAGAGGAGAGGGGAAACGTAGGGGCCCATGGCTCCGGAGCCCTTGTAAATATCC
TATCTAAATTACTATTTAGATTGTGAAACTGAAATAAGAACTTTTCAGGCAGCTTCTCACGG
ATTATCTGCAAGGCGACGTCTTTGGAAGGTGCGTTCAAGGCTTCTGCGGCAGCGTCGTTAGC
TGTCTGGCAACCGCCTCTAGCACTTCTTCCGTCGATTTGGAATTCCCCCCACTCGATAGTATC
TCCGTCCTTGTCGACATAGGATTTGACGTCGGACGATGACTTAGCTCTCTGAATGTTCGGAT
GGAAATGTGTTGACCTTGTTGGGGAGACCAGGTCGAAGAATCGTTGATTTTGGCAGCAGTA
GTTGCCCTCAAATTGAAGAAGCACGTGGAGATGAGGCTCCCCATTTTCATGGAGCTCTCTG
CAAACCTTGATGAACTTCTTATTTGTAGGAGTGTTTAGGGTTTTTAATTGGGAAAGTGCTTCT
TCTTTCGATAATGAGCATTTGGGATATGTGAGGAAATAATTCTTCGCTTTTATTTGAAAGCG
CTTTGGAGCTGATGGCATATTTGTAAATATGACCCTTACTACCAAATGGTAGCTGCTCTAAA
ACTCATATGAATTGGTAGTTATGGTAGCTCTTATATAGTAGAAGTTCCTTTAAGGAGATTGC
TACACGTGGCGGCCATCCGTTATAATATTACACACGTGGCGGCCATCCGATATAATATT

>A_DF_529_LR_LVV_25_-_MT215005 Tomato mottle leaf curl virus (ToMoLCV) - DNA A

ACCGGATGGCCGCGCGGGTTTTTTTGACCCGCTCCGTGATGTATTTTTTGTCTTTTACTATGT
GGTCCAGTCAATAAATGACAAATATGACCGTCCAATCAGAAATGGTCCTCAAAGCCTAATT
ATTTAAAAATACTTGGTCACTAAGTTTGGTAAAGTTTATAAATGGTCCTTCCTCGTAATGTTA
TACCAACTTTAAGTCATAATGCCTAAGCGTGATGCCCCATGGCGCTCAATGGCGGGACCT
CAAAGGTTAGTCGGTCCGCCAATTTCTCCCCTCGTGGAGGAATCGGGTCGAAATCAGAGAA
GGCCCAGGCCTGGGTTAACAGGCCCATGTTCAGGAAGCCCAGGATATATCGGATGTATAGA
ACCCCCGATGTACCAAAGGGATGCGAAGGCCCATGTAAGGTCCAATCCTTCGAGCAGCGT
CACGATGTCTCTCATACGGGGAAGGTGATGTGTATTTCCGATGTCACTCGTGGTAACGGTAT
TACCCACCGCGTGGGTAAACGTTTTTGTGTCAAGTCTGTGTATATATTAGGCAAGGTCTGGA
TGGACGACAACATCAAGTTGAAGAACCACACGAATAGTGTGATGTTCTGGTTAGTTAGGGA
CAGGAGACCGTATGGCACTCCCATGGATTTTGGCCAGGTGTTCAACATGTTTGACAACGAG
CCCAGTACTGCCACGGTGAAGAACGATCTCCGCGATCGTTACCAGGTCCTGCACCGGTTCT

ATGCCAAGGTTACAGGTGGACAATACGCCAGCAACGAGCAGGCTATTGTCAAGAGGTTCT
GGAAGGTCAACAACCATGTGGTGTACAACCATCAGGAGGCTGGGAAATACGAGAATCATA
CGGAGAACGCTCTCTTATTGTATATGGCATGTACTCATGCCTCTAACCCCGTGTATGCTACTT
TAAAAATTCGGATCTATTTTTATGATTCGATAACAAATTAATAAAGTTTAAATTTTATTGAAT
GATTTTCGAGTACATAATTTACATATGGTTTGTCTGTTGCGAATTGAACAGCTCTAATTACAT
TATTAATAGAAATGACTCCTAAACGATCTAAATACATATTAACTAAATCCCTAAACCTATTT
AAATAAGTCGTCCCAGAAGCTGTCGTTGACGTCGTCCATATTTGGAAGTTGAGGAAGCACT
TGTGGAGATCCAACGCTCTCCTCAGGTTGTGGTTGAACCGTATCTGTATGTGGTATATCCTTG
TGTTCGTGTGTATCGGGTTCTCTACGTTTGTTATCTTGAAATATAGGGGATTTGGGACCTCCC
AGATAAACGCGCCATTCTGTGCCTGACGTGCAGTGATGAGTTCCTCGGTGCGTGAATCCATT
GCCGATGCAGTCGATGTGTTGGTATATGGTGCAGCCACAGTTTAGGTCTATGCGCTTGCGTC
GAATGACCCTCTTCTTAGCTATCCTGTGTTGTGGTTTTATAGAGGGGGGTGTCGAGGAAGAT
GAATTTTGCATTGTGGAGTGTCCAGTTTTTGAGGGCTGTGTTTTCCTCTTTGTCGAGAAAATC
TTTATAGCTGGCCCCTTCTCCTGGATTGCATAGCACGATTGAGGGTATCCCTCCTTTAATTTG
AACTGGCTTTCCGTATTTGCAGTTTGATTGCCAGTCCTTTTGGGCCCCAATGAGCTCTTTCCA
ATGCTTCATCTTTAGATATTGCGGAGTGATGTCATCAATGACATTGTACTCCGCTTCGTTTGA
GTAAACCCTAGGATTGAAATCCAGGTGTCCACTCAAATAGTTATGTGGGCCTAATGCACGC
GCCCACATCGTCTTCCCCGTCCTCGAATCACCCTCGATGATCAAACTAATAGGTCTCTCCGG
CCGCGCAGCGGGATCCAGACCAAAATAATCATCAGCCCACTCTTGCATCTCCTCCGGAACC
TCAGTAAAGGAGGAGAGTGGAAACGGAGGAACCCATGGTTCCGGAGCCTTTTGAAAGATG
CGTTCTAAATTAGAATGGATGTTGTGATGTTGAAGGACGAAGTCCTTCGGTTGCTCCTCCCT
CAATATATTGAGGGCCTCCATGACGGATCCTGCGTTGAGGACCTTGGCATACGTGTCGTTGG
CAGATTGCTGACCTCCTCTAGCTGATCTGCCGTCGATCTGGAAAACTCCATGATCAATGAAG
TCTCCGTCTTTCTCCACGTAGGATTTGACATCTGATGAGCTCTTAGCTCCCTGAACGTTCGGA
TGGAAATGTGTTGATCTGGTTGGGGATATGAGGTCGAACAATCGTTCGTTTGTGCACTGTAA
TTTCCCTTCGAACTGGATGAGCACGTGGAGATGAGGTTCCCCATCTTCGTGTAATTCTCTGG
CAACTCGGACGAACAATTTATTAACTGGCGTCTGTAACCCTAATAATTGAGATAGAGCTTCT
TCTTTTGTTAAAGAGCACTTTGGATAAGTGAGGAAATAATTTTTAGAGTGGATTCTAAAACG
ACGTGGTAATGGCATTTTTGTAATTAAGGCGTGTACACCAATTGAGTTCTCTCTAAAACTCT
ATGGCAATTGGTGTATTGGGGTACAATATATACTAGAAGTCTCTATAGAACTCTTAATCATA
GTCACACACGTGGCGGCCATCCGTATAATATT

>B_DF_640_AA_LVV_23_MN147864 Tomato mosaic severe dwarf virus (ToMSDV)- DNA B (New species #1)

ACCGGATGGCCGCGATTTTTTGGTGTCCTGACCCGCTACCGCATTTGGAGCTCCGCGTGTTTT
TGATTTATTGACCCGGTCCCACTGTTGGCGTTCCGCATGTTTTGGTGTCCCGTCCCGCTGCCG
CAATTGGGTCTGTGCTCCCACGTGGTGGGTCCTTAGTGCATTCATTCAAACAAGATGAATTC
AAATTAAAGTGGAGTTTATTTATGTCGTCGAATCATATTGCTGCTGATGAGCTTAGTTGTAA
TTCTTGATGTCGATGTTGTTGACTCATCTTTAATTTGAAATTTGAATTATCGTGGCTCGTCGTC
CAACTAAGTCTTATGTTTATAATGATTAGGTCATCTGTCTCTACGAAGGAAAAGTGTGACGT
GGACCAATAATTGTTTAACTACGGACTTTATTTACGTGATATAATGAACAAGGAATTTTATA
TATAAGTGTCTTATTGATTGAGGTTGAATTAATGTGTCAAAATGTATCCTAGTAAATATAGA
CGTGGGGTGACGTCAACTCAGCGACGAGGTTATTCACGATATCCCGTGTTCAAGCGTTCCTA
TGGTGCTAAACGTATCGATGGGAAACGTCGGTCGAGTAATTCGAACAAGTCACAAGAAGA
GAGCAAGATGTCTGCTCAACGTATACACGAAAACCAGTATGGCTCAGAATTTGTGATTGCA
CATAATTGTGCTATATCAACATTTATAACGTTCCCTAGTCTTGGTAAGACGGAGCCCTGCCG

ATCACGTTCATATATTAAGTTAAAACGATTACGTTTTAAAGGTATAGTAAAACTTGAACGTG
TTCAACCTGATGTGAATATGGACGGCTCAACTCCAAAGACCGAGGGAGTATTTTCCTTGGTT
ATTGTTGTTGATCGTAAACCTCATCTGAGTGCATCCGGAAGTCTCCACACATTTGACCAGCT
ATTTGGTGCAAGGATTCATAGTCATGGCAACTTAGCCATAACTCCTTCGTTGAAAGAGCGTT
TCTATATACGACACGTGGTTAAACGTGTTGTGTCGGTTGAGAAGGACACGATGATGATTGA
CCTTGAAGGAGCTACATCCTTGTCTAATAGGCGTTATAATTGTTGGTCTGCGTTTAATGATCT
TGAACGCGATTCATGTAATGGGGTATATTCTAATATTAATAAGAATGCCCTATTAGTTTATT
ATTGCTGGATGTCTGATGTTGTTTCAAGGGCGTCGACATTTGTATCATTTGACCTTGATTATG
TTGGTTGATTATTAAGTTAACCGTGTGTATTTGCATAAAATATAGACAAGTGTGTACTGATT
GTTATATTATAAATCCAAAAAATAATTATAAATAAGATATGGTCTTGCAAAATAAATAATT
GTAATAACTTTATATTTATTTTAATGACTTGGGTTGTTGTGGAGTACAATTTGTGTTAATGCA
CTCTTGGACCGTTGTCCTCACAATATCGTTTAGTTGGGCCAACGACATTGTTATGTTGGATTG
TGTCCTTTGAGCCCCGACTATTGAAGCTGATTCACCTGGGTCTAGCATGGCGGTCCCTAGTC
TGTTGAGTTCTCGATACGGATGTATTGTGTCACCTATGTCGGACTCCGAATTTTCATGGCTGT
ATCCTATCGTGCTTCTGGTAGCCCATGACTCTCCGGGCTTTAGTTCAATTGGGCCGGGTAGC
CCAAATCTAGACGTGGAAGCGGATCGTATGAGCTTTCTCTCCCATTTTCCGTAGCCCACATG
ACAGAAATCTATGTCTTTTTCTGTGAACTGCTTAGACAAGATTTTAACCGTCGGAGCTCGGA
ATGGGATATCAACTGAATGTTTAGCAGTGGACAGTTTGAGTTTGCCTTTAAATTTGGCGAAA
TGTGTGCTCTGATGTACGTTGGTGTCGCAAACTCTGTAATATAGCTTCCATGGAATTGGGTC
CTTTAGGGAAAAGAAGGAAGAGGAGAAATAATGTAGATCTATGTTACACCTGATCGGAAA
TGTCCATGACGCTTGTAAGGATTCGTTGTCTGTCATCCTTTTGTCATGAATCTCGACAATTAC
CGAACCTGTAGCGTTGATAGGTACTTGTTGTCTGTATTCGATGACGCAGTGGTCGATCTTCA
TACAGCTACGACTGAGCCTTGCGCTTAATTGAGCTGTAGTAGACGGAAATTGCAAGATGAT
CTCAGTTAGATCATGCGATAGTTGATACTCGTCGCGATGAGATTCGATATAATTAAAGGCGT
TTGGTGGGTGTACTAGCTGAGAATCCATTATAAGAATTAAACTTATGAAATCAGGTTGCGC
AGCGAAAGCGTTTAAGGAAAATGAAAGAGGAAAGAGAATAAGTTATGTAAACTGTGTGGG
TAATTGATATTGGCACTGCGTTTAAGAGGTTTGTCTCTTTATATAGACAACGAACTGTTTATT
GAGTATTAAATAGACGTATTGAGTATTAAATAGACGTATTGTGAAGTTTTGTTGTCATTTAA
TCCGTTAATGGCATTTTAGTAAATATGTGTGAGGACACCAGCGATGGCTCTCAACTTCTATG
CAATGTGTTGGTGTCCTGGTGTCCCATTTATACTAAAACCCTCTAGGACACTGGGGGGTACG
TGGCGGCCATCCGTTAATATT

>A_DF_640_AA_LVV_MN147863 Tomato mosaic severe dwarf virus (ToMSDV)- DNA A (New species #1)

ACCGGATGGCCGCGATTTTTTGGTGTCCCGACCCGCGCGCGCCCATTGCGCTGCTTTCTGAC
GATGGTGGTCCCATGTGTCGCAATTGTTTCGCGTGAAAGCTTATTTATTTCAAAATACTTCGC
AGCCAAGTTTGGTAAAGTTGTATATATTGTGTATAGATTGTTGACTGACATTTGCCTTTAATT
CAAAATGTCTAAGCGGGCTGCCCCCTCGTGGCGCTCTATGGCGGGTATCTCAAAGGTTAGC
CGCTCACTTAATTTCTCGCCTCGTGGAGGTATTAATCCCAAATTTGATAGGGCCTCAGCGTG
GGTCAATCGACCCATGTACAGGAAGCCCAGGATATATCGGACTATTCGAAGCCCTGATGTC
CCTAAAGGGTGTGAGGGGCCTTGTAAGGTCCAATCATTTGAACAGCGCCACGATATCTCCC
ATACCGGGAAGGTGCTGTGTATATCTGATGTGACACGGGGCAGTGGTATTACCCACCGTGT
CGGTAAGCGTTTTTGCGTGAAGTCCGTTTATATTTTAGGCAAGGTATGGATGGACGAGAAC
ATCAAGTTGAAGAACCATACGAACAGCGTCATGTTCTGGTTGGTTAGAGACAGGAGACCCT
ATGGCACGCCGATGGATTTTGGACAGGTGTTCAACCTGTTTGATAATGAGCCCAGTACTGC
GACTGTGAAGAACGATCTCCGGGATCGTTTCCAAGTGATGCACAGATTCCACACTAAGGTG

ACGGGTGGACAGTATGCGAGCAACGAGCAGGCTCTTGTCCGGCGATTTTGGAAGGTCAAC
ACCCATGTTGTGTACAACCATCAGGAAGCTGCGAAGTATGAGAACCACACGGAGAACGCC
CTCTTATTGTATATGGCATGTACTCATGCATCTAACCCTGTGTATGCAACCCTTAAGATCCG
GATCTATTTCTACGATTCGGTATCGAATTAATAAAGTTTGAATTTTATTGAATGATCTTCGAG
TACAGATGTTACATATGGTCTGTGTGTTGCGAAACGAACAGCTCTAATTACATTATTAATGG
ATATAACGCCCAAACTATCTAAATAAAACATGATGAAATGTCTAAATCTATCTAAATATGT
CGTCCCAGAAGCTGTCACTGAAGTCGTCCAGACTTGGAAGTTCAGGTATGCCTTGTGGAGA
TCCAACGCTCTCCTGAGGTTGTGGTTGAACCGTATCTGGATGTGGTAGACTCTGGTCGTGGT
GTAGAGTGGGTCTTCGACTCGGTATATCTTGAAATATAGGGGATTTGGTACTTCCCAGATAT
ACACGCCATTCTCCGCTTGAGGTGCAGTGATGAGTTCCCCTGTGCGTGAATCCATGACCGAC
GCAGTTGATGTGGACGTATACTGAGCACCCGCAGTTGAGGTCTATTCGTCTACGTCGAATTG
CCCTGCGCTTGGCTGCTCTGTGTTGAGCTTTGATAGAGGGGGGATTCGAGGAAGACGAACC
TCGCATTCTTGAGAGTCCAATTTCTTAGGGATGCGTTTTCCTCTTTGTCGAGGAAATATTTAT
AGCTGGCTCCTTCGCCAGGATTGCAGAGCACGATTGATGGTATACCTCCTTTAATTTGAACT
GGCTTTCCGTATTTGCAGTTTGTCTGCCAGTTGCTTTGGGCCCCAATTAATTCTTTCCAGTGCT
TTAACTTTAAATAATGAGGAGGGATGTCATCAATGACGTTGTACTCTGCATTATTGTTGTAA
ACCCTAGAATTCAAATCAATGTGGCCGCTGATATAATTGTGGACCCCCAATGCTATGGCCC
ACATCGTCTTCCCCGTCCTCGAATCCCCCTCAATAATTAAACTAATAGGTCTCTCCGGCCGC
GCAGCGGAACCCCTTCCAAAATAGTCATCTGCCCATTCCTGCATCTCCGCGGGAACGTTAG
TGAACGATGAGAGGGGAAACGGAGGAACCCTCGGTTCAGGAGCTTTTTGGAAGAGACGTT
CAATGTTAGCCTTTACGTTATGATAACTTACAACGAACGTCTTGGGGTCTCCTGCTCTGATA
ATCTCCAGAGCCTCTCCTGCACTAGCTGCATTAACTGCGTTGTGGTAGACGTCGTCTTTATTG
GCCTTCGTACCCCCAGACACCTTGTATTGTCCGGATTCACAATAATCACCCTCTTTGGTGAT
GTAATTCTTGACGGCGTTGGTGTCCTTGGCTGCCTGAATATTTGGGTGAAAAGTGGCAGACC
TTCTGGGGTGAGTGAGGTCGAAAAATCTAACATCCTTGATGTTAGACTTTCCTGAGAGTTGT
ATAAGACAATGGAGGTGGGGGAATCCGTCGGAGTGTTCCTCCCTGGCGACTCGTATATATG
TGGGTTTGACGATTGACCATGGAAGGGATTGAAGCATTTGAAGAGCTTCATCTTTTGGTATA
TCGCATTGGGGATATGTGAGGAATATATTTTTGGCAGCAAGCCTAAATTGATTAGGGTTTCG
TGGCATTTTGGTAAATATAGGGCAGGACACCAGCAATGGCTCTCAACTTCTATGCAATGTGT
TGGTGTCCTGGTGTCCCATTTATACTAAAACCCTCTAGGACACTGGGGGGCACGTGGCGGC
CATCCGTTTAATATT

>B_GO_308_LR_LVV_24_-_MT215004 Tomato chlorotic mottle virus (ToCMoV) - DNA B

ACCGGATGGCCGCGCGATCTTTCTCACTCTTTTAATCCTGTCCGTCCACGTGTTTAATGAATT
TAGTTGAGCGCGAATTTGAAGTCCGTGAATTGTGTTGAGCGCTTTATTTGAGTTCCGCTTTCG
GATGGCCGCCACGTCCATATATCTAAGACCGTTGAATTAAATATGATAAGCGGAACTTTAT
CGAATTTTGCATGGTGTTTTTTATTGGTGATTAATTTTGAAATTCGACCGTTAGATTGCACTT
GATATTGAATATCGACCATCGTTCTCGAATAATGTGTCATTTGTACAAGTATTTGTTATGTGT
GCAATCATACAACATTACCTTAACTTGGACCAATTAAATATTGACAGTATAACCAAAATAA
CCCATTGATGCAATTACAGCTATCTATATAATGCGATTGATATGGATAATTATAAAACGTCC
AGTAAGAATTTTGAACATGTATTTTAATACTAATAAGCGTCGTTGGTTATCCATCAACCGTC
GTAATTACGTGCGATATCCTACGTTTAAAAAGTCACATTCTGTGGTACGCAATGATGGGAA
ACGTCGACTGGGTATTTCAAACAAGCCCCACGACGATAGTAAGATGTCTTCGCATACGATT
CATGAAAACCAATATGGGCCTGACTTTGTATTGGGCCATAACACAGCGTTATCCACTTTTAT
AACTTACCCCTCTCTTGGTAAAAGTGAGCCCAATCGTATTAAGTCATACATTAAGTTGAAAC
GTCTGCGCTTTAAAGGTACTGTTAAGATTGAACGTGTTCCTACCGATATGAACATGAATGGA

ATACCTGCTAAGATTGAAGGGGTATTTTCTTTGGTTATTGTTGTCGATCGTAAACCTCATTTG
AGTCCTTCTGGTAGTCTCTACACATTCGATGAGTTATTTGGTGCTAGGATCCATAGCCATGG
AAATTTAGCGATAATCCCGTCTCTGAAGGATCGTTTTTACATACGTCATGTTGCTAAACGTG
TCTTGTCAGTGGAGAAGGATAGTAATATGATTGACGTTGCTGGGACTACAACACTCTCTAGT
AGGCGTTATAATTGCTGGGCAAATTTTAGAGATTTCGATCATGAATCCTGTAACGGTGTATA
TGCTAACATAAGCAAGAACGCCCTACTAGTTTATTATTGTTGGATGTCGGAAACGGTTTCCA
AAGCATCTACATTTGTATCATTTGATCTTGATTATATTGGATAATTATATAGATCAATTTGCA
CATTATAAGTTGATAAGTTTAAATATGATAATAAATTGTCCTAGTGCTTATTAAATACTCAG
AAAATCTTAATTTATTTTAAAGACTTTGGTTCAGATGGAGTACAATTGGTATTAATACATTCT
TGTACTGTTGACTTAACAATCTCATTTAATTGGGCCAAAGATAATGTAATGTTGGACTGCGT
TCTTTGCGCCGCAACGATTGACGCTGATTCTCCTGGGTCTAGAATTATCGTGTCCAATCTGTT
CAGGTCCTTGTATGGATTTAGATCGTTGCCCCGTTCGGAGTCCACACATGAATTGCTAAGTC
CAATTGTACTTCTGGAGGCCCACGACTCTCCAGGTTTAATTTCTATTGGGCAGTTAAGCCCA
AATCTGGACGTCGAAGCGCATCTGACTAATTTCCTCTCCCATGTCCCGTAACCCACGTGAAC
AAAATCGATATCCTTATCTGAAAATTGCTTCGACAATATTTTAACTGTCGGTGCCCGGAAAG
GGATATCTACTGAGTGTTTTGCTGTGGATAGTTTTAACTTGCCCTTGAACTTGGCGAAATGTG
TCCTTTGGTGCACATTCGTATCGCTAACCCGGTAGTACAATTTCCATGGAATTGGGTCCTTC
AGGGAAAAGAATGACGACGAGAAATAATGTAAGTCTATGTTACATCTAATTGGGAAGGTC
CATGACGCCTGCAATGACTCGTTGTCCGTCATTCGCTTGTCATGTATTTCTACTACGACGGAT
CCTGTGGCGTTTATCGGGACTTGCTGTCTGTATTCGATGACGCAATGATCTATTTTCATGCAG
CTCCGACTGAGTTTAGCACTAATTTGAGATGCCGTAGATGGAAATTGTAAAACAATTTCAGT
TAGATCATGAGACAATTGATATTCATCTCTTTGAGACTCAACATAATTAAAAGCATTTGGAG
GGATAACCAATTGAGAACTCATTATAATAAAAAAAGGCTGCGCAGCTGAATCGATAACTG
GAATTGATAAGCGTAATTGATATGAACCCACTAAGAACAATGTCAGAAGACAATATGTGTT
GCGCTGTGACGATTAACGAAATATATTGTGGTAATGATGATAGTCAGTACTAACTGGTTTGT
ATTTATAGACGGATTGAAGACTGAGTATATACGAGGAAACAATGCATAGCGACGTCGTTCT
TTGACGGAATGGCATTCCTCTAAATTAAGATAATGGCATATTTGTAATAATAGTTGGGCACC
GATTGGGGTCTTCTCAAACTTACTAAAGCAATTGGGGACTGGGGTCTTATATATACTAGAAC
CCTCTATAGAACTTTCAATCTCGTTCACACACGTGGCGGCCATCCGATATAATATT

>A_GO_308_LR_LVV_24_-_MT215003 Tomato chlorotic mottle virus (ToCMoV) - DNA A

ACCGGATGGCCGCGCGATTTTTTTAAGGCCCATTTATTTTGGGCCTCTCTTTTGGGCCTTCAC
CTTTAATTTTATTGAAAGTAAATTTGGCCCAATCATGATGCATTTGACGAGCTAAGATATTT
ACAACTACTTAACGCCCAAGTTGCATAACGGCTATAAATTAAACGTGCATATGTCGATAAC
TTTAATTCAAAATGCCTAAGCGCGATGCCCCATGGCGCCACATGGCTAGTACATCTAAGAT
TAGCCGGGGCGTTAATAATTCTCCTCGAGCAGGAGTTGGGCCACGGTCCAACAAGGCCTCT
GACTGGGTAAACAGGCCCATGTACAGGAAGCCCAAGATATATCGGATGTATAGAACCCCC
GATGTTCCAAGGGGTTGTGAAGGCCCATGTAAAGTGCAATCGTTTGAACAGCGTCACGATA
TCTCCCATACTGGTAAGGTGATATGCGTCTCCGATGTGACACGTGGTAACGGTATTACACAC
CGTGTGGGTAAGCGTTTCTGTGTTAAGTCTGTGTATATTCTGGGTAAGATATGGATGGATGA
GAACATCAAGTTGAAGAACCACACGAACAGTGCCATGTTCTGGTTGGTCAGGGACCGTAG
ACCTTATGGTACCCCTATGGACTTTGGCCAGGTGTTTAACATGTTCGACAACGAGCCTAGCA
CCGCCACTGTGAAAAACGATCTCCGTGATCGTTTTCAAGTCATGCATAAGTTCTATGCTAAG
GTCACAGGTGGACAATACGCAAGCAACGAGCAGGCGCTTGTCAAGCGGTTCTGGAAGGTC
AACAATCATGTCGTCTACAACCATCAAGAAGCTGGAAAATACGAGAATCATACGGAGAAC
GCCCTGTTATTGTATATGGCATGTACTCATGCCTCAAACCCCGTGTATGCAACTTTAAAAT

TCGGATCTATTTTTATGATTCGATAACAAATTAATAAATTTTGAATTTTATTGAATGATTCTC
GAGTACAGCATTTACATATCGTTTGTCTGTTGCGAAGCGAACAGCTCTAATTACATTATTAA
CTGAAATAACGCCTAGCTGATCTATGTACAACATAAGTAAATATTTAAATCTATTTAAATAT
GTCATCCCAGAAGCTCTCAGGGATGTCGTCCAGATTTGGAAGTTGAGATAAGCCTTGTGGA
GACCCAGTACTCTCCTCAGGTTGTGGTTGAACCGTATTTGGACGTGGTAGACTCTGGTCCTC
GTGTATAGTATGTCCTCGACGTTGTACATCTTGAAATAAAGGGGATTTGATATTTCCCAAGT
ATACACGCCATTCTCTGCCTGATGTGCAGTGATGAGTTCCCCTGTGCGTGAATCCATGTCCT
GCGCAATCTATGTGTACGAAAATGGAGCAGCCGCACTGTAAATCAATTCGTCTTCTTCGTAT
AGCTCTCTTCTTAGCAGCCCGATGTTGAACTTTGATAGAGGGGGGAGTTGAGGAAGATGAA
TTGTGCATTGTGCTTTGTCCAACTACTTAATGCTGAGTTCTCCTCTTTGTCGAGGAAACATTT
ATAGCTGGCCCCCTCGCCAGGATTGCAAAGCACGATGCATGGGATACCACCTTTAATTTGA
ACTGGCTTTCCGTATTTACAGTTTGATTGCCAATCCTTTTGGGCCCCAATCAATTCTTTCCAG
TGCTTTAACTTTAGATAGTGCGGTGCGACATCATCAATGACGTTATACTGCACATCGTTTGA
GTATACCTTTGAATTGAAATCGAGGTGACCACTCAAGTAATTATGGGACCCTAATGCACGT
GCCCACATAGTTTTGCCACTTCGAGAATCACCCTCGATTATTATACTGATAGGTCTCTCCGG
CCGCGCAGCGGCATCTCTCCCGAAATAGTCATCTACCCAATCTTGCATCTCGACAGGCACG
TTAGTGAACGACGAGAGTGGAAACGGAGGAACCCAAGGTTCTGGAGCCTTTGCGAAAATA
CGCTCTAAATTTGACCTAATGTTATGATGTTGAAGCACGAAATCTTTGGGTTGTTCCTCTTTT
AATATATTGAGTGCTTCCATGACTGATCCTGCGTTGAGAACCTTGGCGTACGTGTCGTTGGC
AGATTGCTGACCTCCTCTAGCTGATCTGCCATCGATCTGGAAAACTCCATGATCAACGAAG
TCTCCGTCTTTCTCCACGTATGATTTGACATCTGACGAGCTCTTAGCTCCCTGTATGTTTGGA
TGGAAATGTGCTGACCTACTTGGGGAGACGAGGTCGAAGAACTTGTTGTTTTTGCAGTTGTA
TTTACCTTCGAATTGGATGAGCACGTGGAGATGAGGTTGCCCATCTTCGTGTAGTTCTCTCG
AGACACGAATGAATAATTTATTAGTTGGTGTGTTTAGGGCTAATAATTGGGAAAGTGCTTCT
TCTTTGGAAAGAGAACAGTGTGGATATGTTAGAAAATAATTTTTGGAATTTACTCGGAAAT
GTTTGGGCGGTGGCATATTTGTAATAATAGTTGGGCACCGATTGGGGTCTTCTCAAACTTAC
TAAAGCAATTGGGGACTGGGGTCCTATATATACTAGAACCCTCTATAGAACTTTCAATCTCG
TTCACACACGTGGCGGCCATCCGATATAATATT

>A_GO_305_LR_LVV_09_MN928612 Tomato golden vein virus (TGVV) - DNA A

ACCGGATGGCCGCGCGATTTTTTAAAGTGGTCCCACTGTGAGTGACCAATCAGATTGTGTCT
TGGACGGTTATTTATTTCGAAATACTTAGGCGCTAAGTTGTTAAAGTTGTATAAATTAGACA
TTCATAAGTGGTCGACATACTTTAATTCAAAATGCCAAAGCGAGATGCCCCATGGCGTATA
GTGGCAGGGACCACGAAAGTATCCCGCTCTTCTAATTATTCACCTCGGGGAGGTATACCCA
AGCGGGATGCTTGGGTAAACAGGCCCATGTACAGGAAGCCCAGGATATATCGTACGTTGA
GAGGGCCTGATGTTCCTAAAGGGTGTGAAGGCCCATGTAAAGTTCAATCCTATGAGCAACG
TCATGACATTTCTCATCTTGGCAAGGTGATGTGTATCTCTGATGTGACACGTGGTAATGGTA
TTACACACCGTGTTGGTAAGCGTTTTTGCGTTAAGTCTGTGTACATATTGGGTAAGATATGG
ATGGACGAAAATATTAAACTGAAGAACCATACCAACAGTGTTATTTTTTGGTTAGTGAGGG
ATCGTAGACCCTATGGGACACCTATGGATTTTGGTCAGGTGTTTAACATGTTTGATAATGAG
CCTAGTACTGCTACGGTGAAGAACGATCTTCGTGATCGTTATCAAGTCATTCATCGATTCAA
TGCTAAGGTTACGGGTGGACAGTATGCTAGCAACGAGCAAGCTCTTGTTCGGCGTTTCTGG
AAGGTCAATAATCATGTGGTTTATAATCATCAGGAAGCAGCGAAGTATGAGAATCATACGG
AGAATGCTCTGTTATTGTATATGGCATGTACTCATGCCTCTAACCCCGTGTATGCTACATTGA
AAATTCGGATCTATTTTTATGATTCGATTACTAATTAATAAAATTTAAATTTTATTGAATGAT
TTTCGAGTACAGCATTTACATATGATTTGTCTGTTGCGAAACGAACAGCTCTGATTACATTA

TTAATGGAAATAACACCTAATTGGTTCAGATACAATAAAACGAAATATCTAAATCTAGTTA
AATATGTCATCCCAGAAGCTCTCAGGGATGTCGTCCAGACTTGGAAATTTAGAAATGCCTT
GTGGAGACCCAACGCTTTCCGCAGGTTGTGGTTGAAACGTATCTGCACATGGTATATCCTGG
TCGTTGTATAATAGGGGTCCTCGACGTTGGTTATCTTGAAATAGAGGGGATTTGTAATCTCC
CAAATAAAGACGCCATTCTCTGCTTGAGGCACAGTGATGGGTTCCGCGGTGCGTGAATCCA
TTATTTCTACAGTTGATGTGGATGTATATGGAGCAGCCGCAGTTTAGGTCTATGCGTTTACG
CCTGGTTGTTTTCTGTTTGGCGGCTCTGTGCCGTGGCTTGATAGAGGGGGGAGTTGAGGAAG
ATGAATTTAGCATTATGCAGTGTCCAAGCTCTTAGAGATGCATTTTCATGTTTGTCAAGGAA
GTCTTTATAACTAGCCCCCTCTCCTGGATTGCAAAGCACGATTGATGGGATCCCTCCTTTAA
TTTGAACCGGCTTTCCGTACTTGCAATTGCTTTGCCAGTCACGCTGGGACCCAATTAGTTCTT
TCCAGTGCTTCATTTTTAGATAATGCGGAGTGACATCATCAATGACGTTATACTCCACTTCA
TTTGAATAAACTTTAGAATTAAAATCCAAGTGTCCACTTAAATAATTATGTGGTCCTAGTGC
ACGAGCCCACATAGTCTTCCCCGTCCTAGAATCACCCTCAATGATGATACTAATAGGTCTTT
CCGGCCGCGCAGCGGCACCCCTTCCGAAATACTCATCAGCCCATTGCTGCATCTCATCAGG
AACGTGAGTAAAGGAAGATAATTCAAATATAGGGACCCATGGGGCTGGAGCCTTTGCAAA
TATTTTTTGGACATGTGATTGTATTTTATCCAAATGAAGAACATAATCTCTAGGTTGTTCTTC
TTTTAATATATTTAAGGCCTTGGATAGATTTTCTGCGTTTAGAACCTTCGCATAAGTGTCATT
GGCAGTTTGGCAGCCTCCTCTTGCTGATCTTCCATCGATCTGGAAATTTCCAAAATCAATGA
AGTCTCCGTCTTTTTCCATGTAGGTCTTGACGTCGGACGAGCTTTTAGCTCCCTGAATGTTTG
GATGGAAATGTGCTGATCTTGTTGGGGATACCAAGTCGAAGAATCGTTGATTCTGGCATTTG
AATTTGCCTTCGAATTGAATAAGCACATGGAGATGAGGTTCCCCATCTTCGTGAAATTCTCT
GGTTACACGAATAAATTTCTTGTTTGTTGGTGTTTTAAGTGCCTGTAATTGGGAAAGTGTTTC
CTCTTTGGTTAATGAACAATGAGGGTATGTAAGGAAGAAATTTTTTGCATTTACAGTGAAAC
GCTTTGGTGGTGGCATTGTAGTAAATAAGAAGATGAGACCCGATAGCTCTCCTTCAAACTT
GGCGAAATGAATTGGGGAATGGGTCTCAATATATAGTAGAGTCCATTATAGAATATAATTG
CCACGTGGCGGCCATCCGATATAATATT

>B_GO_305_LR_LVV_09_MN928613 Tomato golden vein virus (TGVV) - DNA B

ACCGGATGGCCGCGCGATTTTTTTTTGGTGGACCCATTCTGAAAGTAATTGAGCGCATTTTT
GACGTCCGCGAATTGAGTTGAGCGCAATATTTGAGTTCCGAAAATATAGTTAGTGGAGTAA
CTTTAATTTGAAATAAATTACAGCTTTTAGGGTAACCAATCAGCTTGCGATTCAGGAGTCTA
TTTTTTTAGGGTAACCAATCAGCTTGCGATTCAGGAGTCTATTTATCTATTACATTTATGTCA
GTGTCAAGGTATACGGTATTCTTCACGTGGACCAATTAATTGTTCGCTATGGAGTCTAACTA
ATTAATATACGAAGTGATGATTTTTTTATATAAATTCCACATATATTGTGGTAGATATGATAT
CGCATCATGTCTCCTACTAGCTATAGACGTGGTTGGTCGTTTGCTCAACGACGAGGTTATTC
TCGTAAATATTTGTTCAAACGTCCTTATTATTTTAAACGTACAGATGGGAAACACCGATCGG
GTAACTCGAATCAGGTCAATGAGGATATTAAGTTGTCGCAACAGCGTATACATGAGAACCA
GTTTGGTCCGGAATTTGTAATGGGTCATAATACAGCAATATCGACTTTTATCACTTTTCCTAG
TCTATGTAAAACTGAACCGGGCAGATCCAGGGCATACATTAAGTTGAAACGTTTACGTTTC
AAAGGTACCGTCAAGATTGAGCGTGTTCATGCTGATGTGACTATGGATGGTATAATACCAA
AGATTGAAGGCGTATTTTCTATGGTTGTCGTTGTTGATCGTAAACCCCATTTGAGTTCATCTG
GTTGTCTGCATACATTTGATGAGCTCTTTGGTGCACGGATCCACAGTCATGGCAATTTGGCT
GTAACTCCAGCTTTGAAAGAGCGTTTTTACATACGTCACGTTTGGAAGAAAGTAATATCTGT
TGAGAAGGACAGTATGATGGTTGATCTTGAAGGAACGACATCGTTGAGTAATAAGCGTTTT
AATTGTTGGTCTGCTTTTAAAGATCTTGATCGTGATTCATGTAATGGTGTTTATGCGAATATA
AGCAAGAACGCCCTTTTAGTTTATTACTGTTGGATGTCGGATACGATGTCTACGGCGTCATC

ATTTGTATCGTTCGATCTTGATTATGTTGGTTAAAAATTAAAATATTAATGTATTTATACTTA
CATGAGAAAATAATTCATTTAAGAAAACGTGAGAAAATAAATCATTTAACAAAACGTAAG
AAAATAAATCATTTAAGAAAACGTAATCACATAATATAATTATAACTACTGAAATATTGGC
TGCGCAGCTGCATGCAAAAAAAAATATTGGCAACTTGATATTTATTTCAATGATTTGGGTTG
TGGAGGAATACAGTTAGATTTAATACATTCTTGGACTGTTGACCTAACAAGATCGTTTAATT
GGGCCACTGACAGCGTGATGTTGGATTGCGTTCTTTGGGCCCCAATTATTGAAGCCGAATCA
CCTGGATCTAATATGGCAGTTCCTAGCCTATTTAGATCTTTGTATGGATGTAACGCCTCCCCT
ATGTCTGATTGGGCTTCTAATTGGGTCGTCTCTATTGTACTCCTTGTGGCCCATGACTCTCCT
GGATTTAGTTCTATTGGGCCATGAAGCCCAAATCTAGACGTTGATGCCGATCTTATCAATTT
TCTCTCCCACTTTCCATAACCTACATGAGAGAAGTCGATGTCATTGACGGTAAATTGTTTGG
ACATTATCTTCACTGTGGGTGCCCTGAAAGGTATATCAACCGAATGTTTTGCCGTCGATAGC
TTCAATTTTCCTTTGAATTTTGCGAAGTGGGTCATCTGATGAACGTTTGAGTCAGAAACTTTA
TAATATAGCTTCCATGGAATTGGGTCTTTGAGTGAGAAAAATGATGACGAGAAATAGTGGA
GGTCTATGTTGCACCTGATTGGAAAAGTCCATGACGCCTGTAAGGATTCATTTTCAGTCATC
CTCTTGTCATGAATCTCCACAATTACTGATCCAGTTGCGTTGATTGGTACCTGTTGTCTATAT
TCAATGACGCAATGATCAATCTTCATACAGCTTCGATTTAGTCTTGCACTTAATTGAGTTGCT
TTTGATGGAAATTGCAATATGATTTCAGTAAGGTCATGAGATAGTTGATATTCATCACGACG
AGATTCTATATAATTAAAGGCGTTGGGTGGGCAAACTACTTGAGAGTCCATATGTATTAGA
AGATTGAATAAGTGTAGAGAATAATTTAGAAAAAGTAAAGGAAACAGTAGTAGTGATAGA
TAAGATGAGTGAATGGATGTGATATTATACAGAATTGTCGATCCTTATATAGACTCTTTACT
GGTTATGGATAATTTGGTTAAACAATACTGAGTATACTTACTAAATAAGTTTATAAAGTTAT
GGTGAAACGCTTTGGTGGTGGCATTGTAGTAAATAAGAAGATGAGACCCGATAGCTCTCCT
TCAAACTTGGCGAAATGAATTGGGGAATGGGTCTCAATATATAGTAGAGTCCATTATAGAA
TATAATTGCCACGTGGCGGCCATCCGATATAATATT

Sample sequences without *Ty*-1 NGS with codes

>A_GO_169_LR_LVV_14_-_MT214096 Tomato yellow net virus (ToYNV) - DNA A (New species #3)

ACTGGATGGCCGCGCGATTTTTGGAGTCCTATGCGCTCTGGCCCAGCCCATTTGAATTAAAG
ATGATATCTTTTCGCTCGACCAATGATATCGCTTCTGGGAAGCCTAGATATTTGTTCCGATAC
TTACGGCCGAAGTTCTTGATTAACGGCTATAAATTAAAGGAAGACTGGTCACAGTCTTTAAT
TCATAATGCCTAAGCGGGACGCCCCGTGGCGCCTGATGGCGGGAACTTCGAAGGTTAGCCG
CTCTACGAACAGTGCCCCTCGTGGAGGGTTTGGGCCTAAGATCAACAAGGCCTCTGAATGG
GTTAACAGGCCAATGTATAGGAAGCCCAGGATATACCGGGCTTTCAGAACTCCCGACGTTC
CTCGAGGGTGTGAAGGGCCTTGCAAGGTGCAGTCGTACGAGCAGCGACACGACGTCTCAC
ATGTCGGCAAGGTGATGTGCATATCCGATGTGACACGTGGTAACGGCATTACCCACCGTGT
CGGTAAGCGTTTTTGTGTTAAGTCTGTGTACATTTTAGGTAAGATATGGATGGATGAGAATA
TCAAGCTCAAGAATCACACGAATAGTGCGATGTTCTGGTTGGTCAGAGATCGTAGACCGTA
TGGTACCCCAATGGATTTTGGGCAGGTGTTCAACATGTTCGACAACGAGCCCAGCACTGCC
ACGGTTAAGAACGATCTCCGCGATCGTTACCAGGTCTTGCATAAGTTCTATGCCAAGGTCA
CCGGAGGACAGTATGCCAGCAATGAACAGGCCCTAGTCAAGAGGTTCTGGAAGGTCAACA
ATCATGTGGTCTACAACCATCAGGAGGCTGGCAAGTATGATAACCACACGGAGAACGCTCT
GTTACTGTATATGGCATGTACTCATGCCTCTAACCCTGTGTATGCTACTCTTAAGATCCGGAT
CTATTTTTACGATTCGGTCACGAATTAATAAAATTTGAATTTTATTGAATGATTTTCGAGTAC
ATGGCTTACATACGATCTGTCTGTTGCGAAACGAACAGCTCTAATTACATTGTTAATTGCGA

TAACCCCTAATTGATCTAGGTACAACAGGACTAAATGTCTAAATCTATTTAAATAAGTCTTC
CCAGAAGCTGTCAGATATGTCGTCCAGACCTGGAAATTCAAATAGGCCTTGTGGAGATGCA
ACGCTTTCCTCAGGTTGTGGTTGAACCTTATTTGGACGTGATACACTCTGGTCGTCGTGAAC
GGTAGATCCTCTACTTTGGACATCTTGAAATACAGGGGATTTATTATCTCCCAGATATAGAC
GCCATTCTCTGCTTGATGTGCAGTGATGAGTTCCCCTGTGCGTGAATCCATGACCTGTACAA
TTGAGGTGCTGATATATGGTACAGCCGCAGTCTAAGTCAACTCGTCGTCGCCGGACGGCTCT
CTTCTTCGCAATCCTGTGTTTCGTTTTGATAGAGGGGGGCGTCGAGGAAGATGAATTTAGCA
TTGTGAAGTGTCCAGGCTTTTAGTGATGCATTTTCCTCTCTCTCGAGGAATGCTTTATAACTG
GCCCCCTCGCCAGGATTGCAGAGCACGATTGATGGAATTCCTCCTTTAATTTGAACTGGCTT
ACCGTACTTACAATTTGACTGCCAGTCTTTTTGAGCACCAATCAATTCCTTCCAGTGCTTTAG
CTTTAGATAATGCGGGGTGACATCATCGATGACGTTATACTCAACATCGTTTGAGTAAACCC
TAGAATTGAAGTCCAGATGTCCACTTAAATAATTATGTGGGCCTAATGCACGAGCCCACAT
CGTCTTCCCTGTCCTAGAGTCACCCTCCACTATGATACTAACAGGTCTCTCCGGCCGCGCAG
CGGAACCTCTACCAAAATAATCGTCAGCCCATTCCTGCATCTCGTCGGGAACGTTAGTGAA
GGAGGAGAGGGGAAACGGAGGAGCCCATGGATCTGGAGCCTTTTTGAACAGCCGATCTAT
GTTAGCCTTGATGTTATGATAATTCACGATGAACGTCTTTGGGTCTCCTGCCCTGATAATGTC
AATAGCCTCTCCGACAGTAGGTGCATTGATGGCGTTGTGGTAGACGTCGTCTTTATTTGTCTT
TGTTCCGCCAGACACCTTGTACTGTCCGGATTCACAATAATCACCCTCTTTGGTGATATAATT
CTTGACGGCGTTGGTGTCTTTGGCTGTCTGAACATTTGGGTGAAAACGGGCAGACCTTCTGG
GGTGAGTGAGGTCGAAAAATCTAACATCCTTGATGTTCGACTTTCCTGAGAGTTGTATGAGA
CAGTGGAGATGGGGATGCCCGTCTGAATGTTCCTCTCTGGCGACTCTGATATATGTCGGTGT
GACGACCGACCACGATAGTCCGAGAAGCATTTGAAGAGCTTCATCTTTTGGAATATCGCAC
TGGGGATATGTTAAGAATATATTTCTAGCTTGTAATCTAAATGATTTGGGGTTTCGTGGCATT
TTTGTAAATAAGATAGGGGACTCCAGGGGTTGAGCTCTCAACTTCTATGTCCTATTTGCTGG
AGTCTGGAGTCCTATATATACTAGAAGGCTAAAATCCTCTTCCAGGACTCCAAGTGGTACG
GACGGGACTCCAGGGGGCAAAAGCGGCCATCCACTTAATATT

>B_DF_667_LR_LVV_22_-_MT214085 Tomato severe rugose virus (ToSRV) - DNA B

ACCGGATGGCCGCGCGATTTTTCTTATCTGCTACGTGGCGAAATCGTGTACGTTGCCTCGCG
CTTTCCATTTTAATTGAGCGCTTTTTTGAAGTCCGCGAAATGAGTTAATTGTCTTTTTNAAAT
CCGCTNTTTGTGAATCACCTTTAATTTGAATTAAAGGTTGGATAGTTCATATTGATCAATCAT
TTCGCTGGTTTATTTCNTGTCGTGGTGTAATTACAACCGTTCGTTAAAAATATAAGAAATTTA
CGACGTGGACTGTCTAAATTTCATCTACATAGTTAATTTGACAAATGAATGCATATTTAAAC
TCTGCTTTCGTGTGGGTTTACACCACGTCTATACATATTGTCCAGGTTATTTTGTATAAGTAT
AATTTTTATTTTGTCTTATCTTATTATATGAACATGTATCCCATTAAGTATAGACGTGGAATG
TTGTTTAATCATCGACGAGGTTACTCATCTAATCCCGTATTTAAGCGTTTACACGGAGCGAA
ACGAAGTGATTTCAAGCGTCGTTCGAGTAATCAGATTAAGAGCATGGATGAGACTAAAATG
TCTGTTCAGCGGATTCATGAGAACCAGTTTGGCCCTGAATTTGTAATTGGCCACAATTCTGC
CATATCCACATTCATTACATTCCCTACTCTTTGTAAGACTGTCCCGAACCGTTGTAGGTCATA
CATAAAGTTAAGACGACTACGTTTTAAAGGAACAATCAAGATTGACCGTGTTCATGCTGAG
GTGAATATGGACGGTACAAGTCCAATGATTGAAGGAGTCTTCTCTCTGGTTGTAGTCGTTGA
TCGCAAACCTCATTTGGGCTCATCTGGAACTCTGCATTCTTTTGATGAGATATTTGGTGCAA
GGATTCATAGCCATGGTAACCTGGCAATAGTATCCTCTCTGAAAGAGCGTTTTTACATACGT
CACGTTTGGAAGAAAGTAATATCTGTTGAGAAGGATACAACCATGGTTGATGTTGAAGGAA
GTACTATTTTATCTAACAGGCGTTTTAATTGTTGGTCATCCTTTAAGGATATTGACCGTGAAT
CATGTAATGGTGTTTATGCAAACATAAGCAAGAACGCCCTGTTAGTTTATTACTGTTGGATG

TCTGATAATGTGTCTAAGGCATCGACATTTGTATCATTTGACCTTGATTATGTTGGCTAAATA
GTAAGTGTAATACGTGATTAGTAATAATGTTTAACTCGCTGTAATAATAAATACAAGTACTT
GTTCAGTAATAATGTTCAACTCGCAGTAATAACAAAAAAATGTGTTTAATAATATAGAATTT
ATTTTAAAGATTTTGGCTGTGAAGGAGTACAGTTGCTGTTTATACATTCATGGGCCGCTGCTT
TAACAAGTTCGTTTAATTGGGCCATTGACAGTGTTATGTTCGATTGGGCCCTTTGAGCACCA
ATTACGGACGCAGAATCACCTGGGTCTAATGTGCCTGTACCCAGTCTATGTAATTGTCTGTA
TGGATGCATCGCGTTCTCTATATCTGAGCCCGCCTCTGATGGGCTTAAGCCTACAGTACTTC
TAACAGCCCATGACTCTCCGGGCATTATTTCAAGTGGGTTGTGAAGGCCCAATCTTGATATT
GAGGCGGATCTGATCATCTTCCTTTCCCATTTGCCATAGCCTACGTGACTGAAATCAATGTC
CTTATCTGTAAATTGTTTTGAAAGGATCTTCACCGTTGGGGCACGGAAAGGTATATCAACCG
AATGTTTAGCCGTCGAGAGTTTCAGTCTCCCTTTGAATTTAGCGAAGTGGGTACTCTGGTGA
ACGTTCGTGTCACATACTCTGTAGTACAATTTCCATGGAATTGGGTCCTTAAGGGAGAAGA
ACGACGACGAAAAGTAGTGAAGATCTATGTTACATCTGATAGGAAAGGTCCACGACGCCT
GTAGTGATTCATTGTCCGTCATTCTTCTGTCATGAATTTCGACTATAACTGAACCTACGGCGT
TAATTGGTACCTGTTGTCTGTATTCTATTACGCAATGGTCGATTTTCATACAGCTACGACTAA
GCCTTGCGCTTAATTGAGACGCCGTTGAAGGGAATTGAAGCATTATCTCGGTTAGATCATGG
GAAAGCTGATATTCATCACGCTTAGATTCTATATAATTAAATGCGTTGGGAGGATTCACAA
GCTGAGATTCCATTATTGAAAATAGGGAGCGCAGCGACAATGTTTGAGGAAAGTTAATAA
GGGAAGATGATAATATTTTCGTCAACTGAATATATGACAAGAAAATTGTCTGTTGATCCAA
TTGGGAAATTGGAGATGATAATAACTAAAAAACGAGGAATCAGGAGACGACGAATTGAG
AGGAGATATTGTCTATTTTAGGTGTAATTGATAAATACCCCTCTATTTGCTCTTTAAATAGAA
TTTTCAAGGCAAGGGCGCTTTGGAGCTGATGGCATATTTGTAAATATGACCCTTACTACCAA
ATGGTAGCTGCTCTAAAACTCATATGAATTGGTAGTTATGGTAGCTCTTATATAGTAGAAGT
TCTTTTTAAGGAGATTGCTACACGTGGCGGCCATCCGTTATAATATT

>A_DF_667_LR_LVV_22_-_MT214084 Tomato severe rugose virus (ToSRV) - DNA A

ACCGGATGGCCGCGCGATTTTTCACCCCTTTAGTTTCAATTAAAGTAAAGTGATTGTCTGTG
GCCCAATCATATTGGGCCTGTCGAGCTTAGATATTTGTAACAACTTAAGGCCCAAGTTGTTA
AACGGCTATAAATTGAACATACACTTTACTTTTGCTTTAATTCAAAATGCCTAAGCGTGATG
CCCCATGGCGTTTAACGGCGGGAACTTCAAAGGTTTCCCGCTCTGTCAATTATTCTCCCCGT
GCAGGATATGGACCCAAATATAACAAGGCCGCTGAGTGGGTGAACAGGCCCATGTACAGG
AAGCCCAGGATCTACCGTACTTTGAGAGGCCCAGATGTTCCTAGAGGCTGTGAAGGGCCTT
GTAAGGTTCAGTCTTACGAGTCTCGTCATGATGTTTCCCATGTCGGGAAGGTGATTTGTGTG
TCTGACGTTACACGTGGTAACGGTATTACTCACCGTGTTGGTAAGCGTTTCTGCGTGAAGTC
TGTATATATTTTAGGGAAGGTATGGATGGACGAGAGCATCAAGTTGAAGAATCACACAAAT
AGTGTGATGTTCTGGTTGGTTAGAGATCGGAGACCTTATTCGACACCTATGGATTTTGGCCA
GGTGTTCAACATGTTTGACAACGAGCCTAGCACTGCAACTGTTAAGAACGATCTTCGGGAT
CGTTTTCAGGTCATGCACAAGTTTTATGCCAAGGTTACTGGTGGACAGTATGCCAGTAATGA
GCAGGCATTAGTGAAGCGCTTTTGGAAGGTCAACAACAACGTAGTCTACAACCATCAGGA
GGCAGGGAAATACGAGAATCATACTGAGAACGCCTTGCTATTGTATATGGCATGTACTCAT
GCCTCTAACCCCGTGTATGCTACATTGAAAATTCGGATCTATTTTTATGATTCGATTACTAAT
TAATAAAATTTAAATTTTATTGAATGATTTTCGAGTACAGCATTTACATATGATTTGTCTGTT
GCGAAACGAACAGCTCTGATTACATTATTAATGGAAATAACGCCTAATCGATCTAGATACA
ATAAGACTAAATATTTAAATCTAGTTAAATATGTCGTCCCAGAAGCTGTCAGTGAAGTCGT
CCATATCTGGAAGTTGAGGAAGCTCTTGTGGAGATGCAATGCTCTCCTCAGGTTGTGGTTGA
ACCGTATTTGGACGTGGTAGACTCTGCTCGCGGTGTACATTGGATCCTCCACTCTGTTTATCT

TGAAATAGAGGGGATTTGATATCTCCCAAATATACACGCCATTCTCTGCCTGATGTGCAGTG
ATGAGTTCCCCTGTGCGTGAATCCATGTCCAGCGCAATCTATGTGTACGAAAATGGAGCAG
CCGCACTGTAAATCAATTCGTCTTCTTCGTATAGCTCTCCTCTTAGCAGCCCGATGTTGAACT
TTGATAGAGGGGGGAGTTGAGGAAGATGAATTGTGCATTGTGCTTTGTCCAACTACTTAATG
CTGAGTTCTCCTCTTTGTCGAGGAAACATTTATAGCTGGCCCCCTCGCCAGGATTGCAAAGC
ACGATGCATGGGATACCACCTTTAATTTGAACTGGCTTTCCGTATTTACAGTTTGATTGCCA
ATCCTTTTGGGCCCCAATCAATTCTTTCCAGTGCTTTAACTTTAGATAGTGCGGTGCGATGTC
ATCAATGACGTTATATTCAACATGATTTGAATAAACCCTAGGATTGAAATCTAGGTGTCCAC
TCAAATAATTATGGGCCCCTAATGCACGTGCCCACATCGTCTTTCCCGTTCGAGAATCACCT
TCAATGATGATACTAATAGGTCGTTCCGGCCGCGCAGCGGCACCTCTCCCAAAATAGTCAT
CTGCCCAAGCTTGCATCTCTCTCGGTACGCTAGTGAAAGAGGAGAGGGGAAACGTAGGGG
CCCATGGCTCCGGAGCCCTTGTAAATATCCTATCTAAATTACTATTTAGATTGTGAAACTGA
AATAAGAACTTTTCAGGCAGCTTCTCACGGATTATCTGCAAGGCGACGTCTTTGGAAGGTG
CGTTCAAGGCTTCTGCGGCAGCGTCGTTAGCTGTCTGGCAACCGCCTCTAGCACTTCTTCCG
TCGATTTGGAATTCCCCCCACTCGATAGTATCTCCGTCCTTGTCGACATAGGATTTGACGTC
GGACGATGACTTAGCTCTCTGAATGTTCGGATGGAAATGTGTTGACCTTGTTGGGGAGACC
AGGTCGAAGAATCGTTGATTTTGGCAGCAGTAGTTGCCCTCAAATTGAAGAAGCACGTGGA
GATGAGGCTCCCCATTTTCATGGAGCTCTCTGCAAACCTTGATGAACTTCTTATTTGTAGGA
GTGTTTAGGGTTTTTAATTGGGAAAGTGCTTCTTCTTTCGATAATGAGCATTTGGGATATGTG
AGGAAATAATTCTTCGCTTTTATTTGAAAGCGCTTTGGAGCTGATGGCATATTTGTAAATAT
GACCCTTACTACCAAATGGTAGCTGCTCTAAAACTCATATGAATTGGTAGTTATGGTAGCTC
TTATATAGTAGAAGTTCTTTCTAAGGAGATTGCTACACGTGGCGGCCATCCGTTATAATATT

>B_GO_118_LR_LVV_21_-_MT214091 Tomato rugose mosaic virus (ToRMV) - DNA B

ACCGGATGGCCGCGCGATTTTTCTTATCTGCTACGTGGCGAAATCGTGTACGTTGCCTCGCG
CTTTCCATTTTAATTGAGCGCTTTTTTGAAGTCCGCGAAATGAGTTAATTGTCTTTTTNAAAT
CCGCTNTTTGTGAATCACCTTTAATTTGAATTAAAGGTTGGATAGTTCATATTGATCAATCAT
TTCGCTGGTTTATTTCNTGTCGTGGTGTAATTACAACCGTTCGTTAAAAATATAAGAAATTTA
CGACGTGGACTGTCTAAATTTCATCTACATAGTTAATTTGACAAATGAATGCATATTTAAAC
TCTGCTTTCGTGTGGGTTTACACCACGTCTATACATATTGTCCAGGTTATTTTGTATAAGTAT
AATTTTTATTTTGTCTTATCTTATTATATGAACATGTATCCCATTAAGTATAGACGTGGAATG
TTGTTTAATCATCGACGAGGTTACTCATCTAATCCCGTATTTAAGCGTTTACACGGAGCGAA
ACGAAGTGATTTCAAGCGTCGTTCGAGTAATCAGATTAAGAGCATGGATGAGACTAAAATG
TCTGTTCAGCGGATTCATGAGAACCAGTTTGGCCCTGAATTTGTAATTGGCCACAATTCTGC
CATATCCACATTCATTACATTCCCTACTCTTTGTAAGACTGTCCCGAACCGTTGTAGGTCATA
CATAAAGTTAAGACGACTACGTTTTAAAGGAACAATCAAGATTGACCGTGTTCATGCTGAG
GTGAATATGGACGGTACAAGTCCAATGATTGAAGGAGTCTTCTCTCTGGTTGTAGTCGTTGA
TCGCAAACCTCATTTGGGCTCATCTGGAACTCTGCATTCTTTTGATGAGATATTTGGTGCAA
GGATTCATAGCCATGGTAACCTGGCAATAGTATCCTCTCTGAAAGAGCGTTTTTACATACGT
CACGTTTGGAAGAAAGTAATATCTGTTGAGAAGGATACAACCATGGTTGATGTTGAAGGAA
GTACTATTTTATCTAACAGGCGTTTTAATTGTTGGTCATCCTTTAAGGATATTGACCGTGAAT
CATGTAATGGTGTTTATGCAAACATAAGCAAGAACGCCCTGTTAGTTTATTACTGTTGGATG
TCTGATAATGTGTCTAAGGCATCGACATTTGTATCATTTGACCTTGATTATGTTGGCTAAATA
GTAAGTGTAATACGTGATTAGTAATAATGTTTAACTCGCTGTAATAATAAATACAAGTACTT
GTTCAGTAATAATGTTCAACTCGCAGTAATAACAAAAAAATGTGTTTAATAATATAGAATTT
ATTTTAAAGATTTTGGCTGTGAAGGAGTACAGTTGCTGTTTATACATTCATGGGCCGCTGCTT

TAACAAGTTCGTTTAATTGGGCCATTGACAGTGTTATGTTCGATTGGGCCCTTTGAGCACCA
ATTACGGACGCAGAATCACCTGGGTCTAATGTGCCTGTACCCAGTCTATGTAATTGTCTGTA
TGGATGCATCGCGTTCTCTATATCTGAGCCCGCCTCTGATGGGCTTAAGCCTACAGTACTTC
TAACAGCCCATGACTCTCCGGGCATTATTTCAAGTGGGTTGTGAAGGCCCAATCTTGATATT
GAGGCGGATCTGATCATCTTCCTTTCCCATTTGCCATAGCCTACGTGACTGAAATCAATGTC
CTTATCTGTAAATTGTTTTGAAAGGATCTTCACCGTTGGGGCACGGAAAGGTATATCAACCG
AATGTTTAGCCGTCGAGAGTTTCAGTCTCCCTTTGAATTTAGCGAAGTGGGTACTCTGGTGA
ACGTTCGTGTCACATACTCTGTAGTACAATTTCCATGGAATTGGGTCCTTAAGGGAGAAGA
ACGACGACGAAAAGTAGTGAAGATCTATGTTACATCTGATAGGAAAGGTCCACGACGCCT
GTAGTGATTCATTGTCCGTCATTCTTCTGTCATGAATTTCGACTATAACTGAACCTACGGCGT
TAATTGGTACCTGTTGTCTGTATTCTATTACGCAATGGTCGATTTTCATACAGCTACGACTAA
GCCTTGCGCTTAATTGAGACGCCGTTGAAGGGAATTGAAGCATTATCTCGGTTAGATCATGG
GAAAGCTGATATTCATCACGCTTAGATTCTATATAATTAAATGCGTTGGGAGGATTCACAA
GCTGAGATTCCATTATTGAAAATAGGGAGCGCAGCGACAATGTTTGAGGAAAGTTAATAA
GGGAAGATGATAATATTTTCGTCAACTGAATATATGACAAGAAAATTGTCTGTTGATCCAA
TTGGGAAATTGGAGATGATAATAACTAAAAAACGAGGAATCAGGAGACGACGAATTGAG
AGGAGATATTGTCTATTTTAGGTGTAATTGATAAATACCCCTCTATTTGCTCTTTAAATAGAA
TTTTCAAGGCAAGGGCGCTTTGGAGCTGATGGCATATTTGTAAATATGACCCTTACTACCAA
ATGGTAGCTGCTCTAAAACTCATATGAATTGGTAGTTATGGTAGCTCTTATATAGTAGAAGT
TCTTTTTAAGGAGATTGCTACACGTGGCGGCCATCCGTTATAATATT

>A_GO_118_LR_LVV_21_-_MT214090 Tomato rugose mosaic virus (ToRMV) - DNA A

ACCGGATGGCCGCGCGATTTTTTTAAGGCCCATTTATTTTGGGCCTCTCTTTTGGGCCTTCAC
CTTTAATTTTAATGAAAGTAATTTTGACCCAATCATGATGCATTTGACGAGCTAAGATATTT
AGAACTACTTAACGCCCAAGTTGCATAACGGCTATAAATTAAACGTACATATGTCGATAGC
TTTAATTCAAAATGCCTAAGCGCGATGCCCCATGGCGCCACATGGCTAGTACATCTAAGAT
TAGCCGGGGCGTTAATAATTCTCCACGTGCAGGAGTTGGGCCACGGTCCAACAAGGCCACT
GACTGGGTAAACAGGCCCATGTACAGGAAGCCCAAGATATATCGGATGTATAGAACCCCC
GATGTTCCAAGGGGTTGTGAAGGCCCATGTAAAGTGCAGTCGTTTGAACAGCGTCACGATA
TCTCCCATACTGGTAAGGTGATGTGCGTCTCCGATGTGACACGTGGTAACGGTATTACACAC
CGTGTGGGTAAGCGTTTCTGTGTTAAGTCTGTGTATATTCTGGGTAAGATATGGATGGATGA
GAACATCAAGTTGAAGAACCACACGAACAGTGCCATGTTCTGGTTGGTCAGGGACCGTAG
ACCTTATGGTACCCCTATGGACTTTGGCCAGGTGTTTAACATGTTCGACAACGAGCCTAGCA
CCGCCACTGTGAAAAACGATCTCCGTGATCGTTTTCAAGTCATGCATAAGTTCTATGCTAAG
GTCACAGGTGGACAATACGCAAGCAACGAGCAGGCGCTTGTCAAGCGGTTCTGGAAGGTC
AACAATCATGTCGTCTACAACCATCAAGAAGCTGGAAAATACGAGAATCATACGGAGAAC
GCCCTATTATTGTATATGGCATGTACTCATGCCTCAAACCCCGTGTACGCAACTTTAAAAAT
TCGGATCTATTTTTATGATTCGATAACAAATTAATAAATTTTGAATTTTATTGAATGATTCTC
GAGTACAGCATTTACATATCGTCTGTCTGTTGCGAAGCGAACAGCTCTAATTACATTATTAA
CTGAAATAACGCCTAGCTGATCTATGTACAACATAAGTAAATATTTAAATCTATTTAAATAT
GTCATCCCAGAAGCTCTCAGGGATGTCGTCCAGATTTGGAAGTTGAGATAAGCCTTGTGGA
GACCCAGTACTCTCCTCAGGTTGTGGTTGAACCGTATTTGGACGTGGTAGACTCTGGTCCTC
GTGTATAGTATGTCCTCGACGTTGTACATCTTGAAATAAAGGGGATTTGATATTTCCCAAGT
ATACACGCCATTCTCTGCCTGATGTGCAGTGATGAGTTCCCCTGTGCGTGAATCCATGTCCA
GCGCAATCTATGTGTACGAAAATGGAGCAGCCGCACTGTAAATCAATTCGTCTTCTTCGTAT
AGCTCTCCTCTTAGCAGCCCGATGTTGAACTTTGATAGAGGGGGGAGTTGAGGAAGATGAA

TTGTGCATTGTGCTTTGTCCAACTACTTAATGCTGAGTTCTCCTCTTTGTCGAGGAAACATTT
ATAGCTGGCCCCCTCGCCAGGATTGCAAAGCACGATGCATGGGATACCACCTTTAATTTGA
ACTGGCTTTCCGTATTTACAGTTTGATTGCCAATCCCTTTGGGCCCCAATCAATTCTTTCCAG
TGCTTTAACTTTAGATAGTGCGGTGCGACATCATCAATGACGTTATATTCAACATGATTTGA
ATAAACCCTAGGATTGAAATCTAGGTGTCCACTCAAATAATTATGGGCCCCTAATGCACGT
GCCCACATCGTCTTTCCCGTTCGAGAATCACCTTCAATGATGATACTAATAGGTCGTTCCGG
CCGCGCAGCGGCACCTCTCCCAAAATAGTCATCTGCCCAAGCTTGCATCTCTCTCGGTACGC
TAGTGAAAGAGGAGAGGGGAAACGTAGGGGCCCATGGCTCCGGAGCCCTTGTAAATATCC
TATCTAAATTACTATTTAGATTGTGAAACTGAAATAAGAACTTTTCAGGCAGCTTCTCACGG
ATTATCTGCAAGGCGACGTCTTTGGAAGGTGCGTTCAAGGCTTCTGCGGCAGCGTCGTTAGC
TGTCTGGCAACCGCCTCTAGCACTTCTTCCGTCGATTTGGAATTCCCCCCACTCGATAGTATC
TCCGTCCTTGTCGACATAGGATTTGACGTCGGACGATGACTTAGCTCTCTGAATGTTCGGAT
GGAAATGTGTTGACCTTGTTGGGGAGACCAGGTCGAAGAATCGTTGATTTTGGCAGCAGTA
GTTGCCCTCAAATTGAAGAAGCACGTGGAGATGAGGCTCCCCATTTTCATGGAGCTCTCTG
CAAACCTTGATGAACTTCTTATTTGTAGGAGTGTTTAGGGTTTTTAATTGGGAAAGTGCTTCT
TCTTTCGATAATGAGCATTTGGGATATGTGAGGAAATAATTCTTCGCTTTTATTTGAAAGCG
CTTTGGAGCTGATGGCATATTTGTAAATATGACCCTTACTACCAAATGGTAGCTGCTCTAAA
ACTCATATGAATTGGTAGTTATGGTAGCTCTTATATAGTAGAAGTTCTTTCTAAGGAGATTG
CTACACGTGGCGGCCATCCGTTATAATATT

>A_GO_384_LR_LVV_19_-_MT214088 Tomato mottle leaf curl virus (ToMoLCV) - DNA A

ACCGGATGGCCGCGCGGGTTTTTTTTGACCCGCTCCGTGACGTGTTTTTTGTCTTTTACTATGT
GGTCCAGTCAATAAATGACAAATATGACCGTCCAATCAGAAATGGTCCTCAAAGCCTAATT
ATTTAAAAATACTTGGTCACTAAGTTTGGTAAAGTTTATAAATGGTCCTTCCTCGTAATGTTA
TACCAACTTTAAGTCATAATGCCTAAGCGTGATGCCCCATGGCGCTCAATGGCGGGGACCT
CAAAGGTTAGCCGCTCTGCAAATTTCTCCCCTCGTGGAGGAATCGGGTCGAAATCAGAGAA
GGCCCAGGCCTGGGTTAACAGGCCCATGTACAGGAAGCCCAGGATATATCGGATGTATAG
AACCCCCGATGTTCCAAGGGGTTGTGAAGGCCCATGTAAAGTCCAATCGTTTGAACAGCGT
CACGATGTCTCTCATACTGGGAAGGTGATGTGTATTTCCGATGTCACTCGTGGTAACGGTAT
TACCCACCGCGTGGGTAAACGTTTTTGTGTCAAGTCTGTGTATATATTAGGCAAGGTCTGGA
TGGATGACAACATCAAGTTGAAGAACCACACGAATAGTGTGATGTTCTGGTTAGTTAGGGA
CAGGAGACCGTATGGCACTCCCATGGATTTTGGCCAGGTGTTCAACATGTTTGACAACGAG
CCCAGTACTGCCACGGTGAAGAACGATCTCCGCGATCGTTACCAGGTCCTGCACAAGTTCT
ATGCTAAGGTCACAGGTGGACAATACGCAAGCAACGAGCAGGCGCTTGTCAAGCGGTTCT
GGAAGGTCAACAATCATGTCGTCTACAACCATCAAGAAGCTGGAAAATACGAGAATCATA
CGGAGAACGCCCTATTATTGTATATGGCATGTACTCATGCCTCTAACCCCGTGTATGCTACT
TTAAAAATTCGGATCTATTTTTATGATTCGATAACAAATTAATAAATTTTAAATTTTATTGAA
TGATTTTCGAGTACAGCATTTACATATGGTTTGTCTGTTGCGAAACGAACAGCTCTAATTAC
ATTATTAATTGAAATAACTCCTAAACGATCTAAATACATATTAACTAAATCCCTAAACCTAT
TTAAATAAGTCGTCCCAGAAGCTGTCGTTGACGTCGTCCCGACTTGGAAGTTCAGGAAGCA
CTTTTGGAGATCCAATGCTCTCCTGAGGTTGTGGTTGAACCGTATCTGGATGTGGTAGATCC
TTGTGTTCGTGTATATTGGATTCTCTACGTTTGTTATCTTGAAATAGAGGGGATTTGGGACCT
CCCAGATAAACGCGCCATTCTGTGCCTGATGTGCAGTGATGACTTCCCCGGTGCGTGAATCC
ATTGCCGACGCAGTCGATGTGTTGGTATATGGTGCAGCCACAGTTTAGGTCTATGCGCTTGC
GTCGAATGACCCTCTTTTTAGCTATCCTGTGCTGTGGTTTTATAGAGGGGGGTGTTGAGGAA
GATGAATTTTGCATTGTGCAGTGTCCAGTTTCTGAGGGCTGTGTTTTCTTGTCTTTCGAGAAA

CTCTTTATAGCTGGCCCCCTCTCCTGGATTACAGAGCACGATTGAGGGTATCCCTCCTTTAAT
TTGAACTGGCTTTCCGTATTTGCAGTTTGATTGCCAGTCCTTTTGGGCCCCTATGAGCTCTTTC
CAATGCTTCATCTTTAGATATTGCGGAGTGACGTCATCAATGACGTTGTACTCAGCTTCGTTT
GAGTAAACCCTAGAATTGAAATCCAAGTGTCCACTCAAATAGTTATGTGGGCCTAGTGCAC
GAGCCCACATCGTCTTCCCCGTCCTCGAATCACCCTCGATGATTATACTAATAGGTCTCTCC
GGCCGCGCAGCGGATCCACACCAAAATAATCATCCGCCCACTCTTGCATCTCCTCCGGAA
CCTCAGTAAAGGAGGAGAGGGGAAACGGAGGAACCCATGGTTCCGGAGCATTCTGAAAG
ATACGTTCTAAATTAGAACGTATGTTGTGATGTTGTAGGACGAAGTCCTTCGGTTGCTCCTC
CCTCAATATATTGAGGGCCTCCATGACGGATCCTGCGTTGAGGACCTTGGCATACGTGTCGT
TGGCAGATTGCTGACCTCCTCTAGCTGATCTGCCATCGATCTGGAAAACTCCATGATCAATG
AAGTCTCCGTCTTTCTCCACGTAGGATTTGACATCTGACGAGCTCTTAGCTCCCTGTATGTTT
GGATGGAAATGTGCTGACCTACTTGGGGAGACGAGGTCGAAGAACTTGTTGTTTTTGCAGC
TGTATTTACCTTCGAATTGGATGAGCACGTGGAGATGAGGTTGCCCATCTTCGTGTAGTTCT
CTCGAGACACGAATGAACAATTTATTAGTTGGTGTGTTTAGGGCTAATAATTGGGAAAGTG
CTTCTTCTTTGGAAAGAGAACACTGTGGATAAGTGAGGAAATAATTTCTAGCATTTAATCTA
AATGGCTTAGGCGGTGGCATATTTGTAATAATAGTTGGGCACCGATTGGTGTCTTCTCAAAC
TTACTAAAGCAATTGGGGACTGGGGTCTTATATATACTAGAACCCTCTATAGAACTTTCAAT
CTCGTTCACACACGTGGCGGCCATCCGTATAATATT

>A_MG_378_LR_LVV_15_-_MT214095 Tomato golden net virus (ToGNV) - DNA A (New species #2)

ACTGGATGGCCGCGATTTTTCCCCGCTCCTGACCCGCTTCCGCAAATCTGGGCCGCAAATC
TGGGTCCTACTAATAATGACAAATTTGACTGACCAATCAGATATCGTCCTCGGAGCTTATTT
ATTTTGGAATACTCCGGCGCGAATTTGTTATATAAAGGGCCATGTTAACATGGCCCAATAA
GCTTTTTCGGAAATGCCGAAGCGGTATGCCCCATTCCGCACAATGGCGGGAACCTCGAAGA
TATCCCGCACGTCCAATATGTCTCCGCGTGGTGGTATCCGGCCCAAATTCGACAAGGGCTCT
GAGTGGGTCAACAGGCCCATGTACAGGAAACCCAGGATATACCGTACTTTACGCAGCCCA
GACGTGCCCAGGGGTTGCGAGGGGCCTTGTAAGGTCCAGTCTTTTGAACAGCGTCATGATG
TGTCTCATACGGGCAAGGTGTTGTGTGTCTCTGATGTGACACGGGGTAATGGTATTACCCAC
CGTGTCGGTAAGCGTTTTTGTGTTAAGTCTGTGTATATATTAGGGAAGGTGTGGATGGACGA
TAATATTAAGTTGAAGAACCATACGAATAGCGTATTGTTTTGGTTAGTTAGGGATAGGAGG
CCGTATGGTAACCCTATGGACTTCGGCCAGGTTTTCAATCTGTATGATAATGAGCCCAGTAC
CGCTACTGTGAAGAACGATCTCCGTGATCGTTTTCAGGTTTTGCACAAGTTCTCCGCCAAGG
TCACGGGTGGTCAGTATGCCAGTAACGAGCAGGCTATTGTGCGGCGTTTTTGGAAGGTCTA
CAACCACGTGGTCTACAACCACCAAGAGACCGCGAAGTACGAGAATCACACGGAGAACG
CCCTGTTATTGTATATGGCATGTACGCATGCCTCCAACCCTGTGTATGCTACCCTTAAGATTC
GGATCTATTTTTATGATTCGGTCTTAAATTAATAAATATTAAATTTTATTGAATGGTCTTCGA
GTACATAATTTACATACGATTTGTCTGTTGCGAAACGAACAGCTCTAATTACATTGTTAATG
GAAATAACGCCTAATCGATCTAGATACAATAAGACTAAATATTTAAATCTAGTTAAATATG
TCGTCCCAGAAGCTGTCAGTGAAGTCGTCCATATCTGGAAGTTGAGGAAGCTCTTGTGGAG
ATGCAATGCTCTCCTCAGGTTGTGGTTGAACCGTATTTGGATGTGGTACACTCTTGTGTTTGC
GTGGAGTAAAACCTCCACGTTTGTTATCTTGAAATAAAGGGGATTGTGCACCTCCCAGGTAT
AGACGCCATTCTCCGCTTGACGTCGCGTGATGCGTTCCTCTGTGCGTGAATCCATGTCCTCG
GCAGTCTATGTGCATGTATATCGAGCAACCGCACTGTAAGTCAATGCGTCTCCTCCTGATGG
CCCTGCTATTCTTCTTAGCTATCCTGTGCTGTGGTTTTATAGAGGGGGGTGTCGAGGAAGAT
GAATTTTGCATTGTGGAGTGTCCAGTTTTTGAGGGCTGTGTTTTCCTCTTTGTCGAGAAAATC

TTTATAGCTGGCCCCCTCTCCTGGATTACAGAGCACGATTGAGGGTATCCCTCCTTTAATTTG
AACCGGCTTTCCGTACTTGCAATTTGATTGCCAGTCCCTTTGGGCCCCAATTAATTCTTTCCA
GTGCTTTAACTTTAGATAGTGCGGTGCGACATCATCAATGACGTTATACTCCACTTCGTTTG
AGTAAACCCTAGGATTGAAATCCAGGTGTCCACTCAAATAATTATGTGGGCCTAATGCACG
CGCCCACATTGTCTTCCCCGTCCTCGAATCACCCTCCACGATGATTGAAACCGGTCTGAAAC
TACTATGAAAATCTGGCCGCGCAGCGGAAAGACGACCAAAATAATCATCAGCCCACTCCT
GCATCTCATCGGGAACGTTAGTGAAGGAGGAGAGTGAAAACGGAGGAGCCCATGGCTCTG
GAGACTTAGCGAATATCTTCTGAATATTCGCATTGATGTTGTGATGTTGAACTATGAAGGCC
TTCGGGTCTCCTGTCCGGATAATTGCAAGAGCCTCTGATGCAGATCTTGCATTGATGGCGTT
GTGATAGACGTCGTCTTTGTTGGTCTTAGTTCCGCCAGACACCTTGTATTGTCCGGATTCACA
ATAATCACCCTCCTTGGTGATGTAATTCTTGACGGCATTGGAGTCTTTGGCTGCCTGTACATT
TGGGTGAAAATTGGTAGACCTTCTTGGGTGAGTAAGGTCGAAAAATCTAGCATCCTTGATG
TTTGACTTACTGGATAGTTGTATGAGGCAGTGGAGGTGGGGGTGTCCGTCGGAGTGTTCCTC
TCTGGCGACCCTAATGTATGTAGGGGTGACGACTGACCATTTAAGGTTTTGAAGCATCTGA
AGAACTTCATCTTTCGTGATATCACACTGGGGATATGTGAGGAAGATGTTCTTTGCAGCAAG
TCTGAACGAATTCTGATTACGGGGCATGTTGTTAAATATATATTTGTGAACAGTTGAGGACA
CCAGGCTTTGCTCTCAACTTCTGTGCTATTTGTTGGTGTTCTGGTGTCCCATTTATACTAAAA
GCCTCTATGAATCTCTTAGGACACCAGGGGTAAAAGCGGCCATCCTTATAATATT

>A_MG_388_LR_LVV_18_-_MT214089 Tomato common mosaic virus (ToCmMV) - DNA A

ACTGGATGGCCGCGCAAAAATATGGACTTGGGCCGCTTTTTCTGGCCCAATTAAAACAGGC
CTGACAAGCTTAGATATTTGTAACAACTTAGAGCCCAAGTTGTATAACGGCTATAAATTGA
ATCTCTATGTTATTTGAACTTTAATTCGAAATGCCTAAGCGAGATGCCCCATGGCCCCACAG
GGCACCTTCGACGAAGATTAGCCGTTCAGCTAATTTCTCCCCTCGTTCGGGAACTGGGCCTG
GGCTTAGCAAAGCCGCTGAATGGGTCAACCGGCCCATGTACAGGAAGCCCAGGATATACA
GAACTATGAGAACGCCCGACGTCCCTAGAGGCTGTGAAGGCCCATGTAAGGTCCAGTCCTA
CGAGCAACGCCACGATATTTCACATACTGGGAAGGTGATGTGCATATCAGACGTCACACGT
GGCAACGGAATTACACACCGTGTCGGTAAGCGTTTCTGCGTTAAGTCTGTGTATATTCTAGG
GAAGATATGGATGGACGAGAACATCAAGTTGAAAAACCATACGAACAGTGCTATGTTCTG
GTTGGTCAGAGACCGAAGACCGTATGGCACCCCTATGGATTTTGGCCAGGTGTTCAACATG
TTTGACAACGAGCCCAGCACTGCAACCGTGAAGAACGATCTCCGTGATCGTTTTCAGGTCA
TGCATAAGTTCTATGCTAAGGTCACAGGTGGACAATACGCAAGCAACGAGCAGGCGCTTGT
CAAGCGGTTCTGGAAGGTCAACAATCATGTCGTCTACAACCATCAAGAAGCTGGAAAATA
CGAGAATCATACGGAGAACGCCCTATTATTGTATATGGCATGTACTCATGCCTCAAACCCC
GTGTACGCAACTTTAAAAATTCGGATCTATTTTTATGATTCGATAACAAATTAATAAATTTT
GAATTTTATTGAATGATTCTCGAGTACAGCATTTACATATCGTCTGTCTGTTGCGAAGCGAA
CAGCTCTAATTACATTATTAACTGAAATAACGCCTAGCTGATCTATGTACAACATAAGTAA
ATATTTAAATCTATTTAAATATGTCATCCCAGAAGCTCTCAGGGATGTCGTCCAGATTTGGA
AGTTGAGATAAGCCTTGTGGAGACCCAGTACTCTCCTCAGGTTGTGGTTGAACCGTATTTGG
ACGTGGTAGACTCTGGTCCTCGTGTATAGTATGTCCTCGACGTTGTACATCTTGAAATAAAG
GGGATTTGATAGCTCCCATATATAGACGCCATTCTCTGCCTGACGCGCAGTGATGCGTTCCC
CTGTGCGTGAATCCATAGTTACTGCAGTTGAGGTTAACGTACAAAGAGCAGCCACAGTTTA
GGTCTATCCTTCTACGTCGAGGTGCTCTCTTCGCAATCCTGTGCCGTAGTTTGATAGAGGGG
GGAGTTGAGGATGATGAATTTAGCATTATGGAGTGTCCACGACTTGAGAGATGCGTTTTCCT
CTTTGTCGAGGAAACATTTATAGCTGGCCCCCTCGCCAGGATTGCAAAGCACGATGCATGG
GATACCACCTTTAATTTGAACTGGCTTTCCGTATTTACAGTTTGATTGCCAATCCTTTTGGGC

CCCAATCAATTCTTTCCAGTGCTTTAACTTTAGATAGTGCGGTGCGACATCATCAATGACGT
TATACTGCACATCGTTTGAGTAAACCCTAGAATTGAAATCCAGGTGTCCACTTAGATAATTA
TGTGGGCCAAGTGCACGCGCCCACATCGTCTTCCCCGTCCGACTATTACCCTCGATGACAAT
ACTGATAGGTCTGATAGGCCGCGCAGCGGCATCCCTTCCAAAATAATCATCAGCCCATTCT
TGCATCTCGTCGGGCACGTTAGTGAATGAGGAGAGGGGAAACGGAGGAACCCACGGTGCC
GGAGCCTTTTGGAAAATCCTCTCTAGATTAGCCTTCACATTGTGATAGTTGACGAGGAAAGT
CTTGGGGTCTCCGGCCCTGATAATGTCGAGAGCCTCTCCCGGAGAAGGTGCATTGACGGCG
TTGTGGTACACGTCGTCTTTATTTGCCTTTGTTCCCCCAGACACCTTGTACTGTCCGGATTCA
CAATAATCACCTTCTTTGGTGATGTAATTCTTGACTGCATTGGTGTCTTTGGCTGCCTGAACA
TTTGGGTGAAATCCGGCAGACCGTCTGGGGTGAGTGAGGTCGAAAAACCTAACATCCTTGA
TATTCGATTTTCCGGAGAGTTGGACAAGACAGTGAAGATGGGGGAACCCGTCGGAGTGTTC
CTCTCTGGCGACGCGTATGTATATGGGTTTGACGACTGACCACGGAAGACATTGAAGCATT
TGAAGAGCTTCATCTTTTGGTATATCGCACTGTGGATATGTTAGAAATATGTTTCTCGCCGTT
AATCTGAATATATTAGGGTTCCGTGGCATTTTTGTAAATATAGGTGAGGACACCAGGGATTG
CTCTCAACTTCTGTGCTATTTGCTGGTGTCCTGGTGTCCCATTTATACTAGAACTCTCTAGGG
TAATCTCAGGGGCAAAAGCGGCCATCCATATAATATT

>B_DF_50_LR_LVV_20_-_MT214087 Tomato chlorotic mottle virus (ToCMoV)- DNA B

ACCGGATGGCCGCGCGATCTTCCTCACTCTTTTAATCCTGGCCGTCCACGTGTTTAATGAATT
TAGTTGAGCGCGAATTTGAAGTCCGTGAATTGTGTTGAGCGCTTTATTTGAGTTCCGCTTTCG
GATGGCCGCCACGTCCATATATCTAAGACCGTTGAATTAAATATGATAAGCGGAACTTTAT
CGAATTTCGCATGGTGTTTTTTATTGGTGATTAATTTTGAAATTCGACCGTTAGATTGCACTT
GATATTGAATATCGACCATCGTTCTCGAATAATGTGTCATTTGTACAAGTATTTGTTATGTGT
GCAATCATACAACATACGTAACGTGGACCAATTAAATATTGACAGTATAACCAAAATAAC
CCATTGATGCAATTACAGCTATCTATATAATGTGATTGATATTGATATTTTTAAAACATCCA
CTAAGAATTTTGAACATGTATTTTAATAGTAATAAGCGTCGTTGGTCGTCCATCAACCGTCG
TAATAACGTACGATATTCTACGTTTAAAAGGTCACATGCTGTGGTACGCAATGATGGGAAA
CGTCGATTGGGTATTTCAAACAAGCCCCTCGACGATACTAAGATGTCTTCGCATAGGATTCA
TGAAAACCAATATGGGCCTGACTTTGTATTGGGCCATAACACAGCGTTATCCACTTTTATAA
CTTACCCCTCTCTTGGTAAAAGTGAGCCCAACCGTATTAAGTCATACATTAAGTTGAAACGT
CTGCGCTTTAAAGGTACTGTTAAGATTGAACGTGTTCCTACCGATATGAACATGAATGGAAT
ACCTGCTAAGATTGACGGGGTATTTTCTTTGGTTATTGTTGTCGATCGTAAACCTCATTTGAG
TCCTTCTGGTAGTCTCTACACATTCGATGAGTTATTTGGTGCTAGGATCCATAGCCATGGAA
ATTTAGCGATAATCCCTTCTCTGAAGGATCGTTTTTACATACGTCATGTTGCTAAACGTGTCT
TGTCAGTGGAGAAGGATAGTAATATGATTGACGTTGATGGGACGACAACACTCTCTAGTAG
GCGTTATAATTGCTGGGCAAATTTTAGAGATTTCGATCATGAATCCTGTAACGGTGTATATG
CTAACATAAGCAAGAACGCCCTACTAGTTTATTATTGTTGGATGTCGGAAACGGTATCCAA
AGCATCTACATTTGTATCATTTGATCTTGATTATATTGGATAATTATATAGATCAATTTGCAC
ATTATAAGTTGATAAGTTTAAATATGATAATAAATTGTCCTAGTGCTTATTAAATACTCAGA
AAAACTTAAATTATTTTAAAGACTTTGGTTCAGATGGAGTACAATTGGTATTAATACATTCT
TGTACTGTTGACTTAACAATCTCATTTAATTGGGCCAAAGATAAGGTAATGTTGGACTGCGT
TCTTTGCGCCGCAACGATTGACGCTGATTCTCCTGGGTCTAGAATTATCGTGTCCAATCTGTT
CAGGTCCTTGTATGGATTTAGATCGTTGCCCCGTTCGGAGTCCACACATGAATTGCTAAGTC
CAATTGTACTTCTGGAGGCCCACGACTCTCCAGGTTTAATTTCTATTGGGCCGTTAAGCCCA
AATCTGGATGTCGAAGCGCATCTGACTAATTTCCTCTCCCATATCCCGTAACCCACATGAAC
AAAATCGATATCCTTATCTGAAAATTGCTTCGACAATATTTTAACTGTTGGTGCCCGGAAAG

GGATATCTACTGAGTGTTTTGCTGTGGATAGTTTTAACTTGCCCTTGAACTTGGCGAAATGG
GTCCTTTGGTGCACATTCGTATCGCTAACCCTGTAGTATAATTTCCATGGAATTGGGTCCTTC
AGCGAAAAGAATGACGACGAGAAATAATGTAAGTCTATGTTACATCTAATTGGGAAGGTC
CATGACGCTTGCAATGACTCGTTGTCCGTCATTCTCTTGTCATGTATTTCTACTATAACGGAT
CCTGTGGCGTTTATCGGGACTTGCTGTCTGTATTCGATGACACAATGATCTATTTTCATGCAA
CTCCGACTGAGTTTAGCACTAATTTGAGATGCCGTAGATGGAAATTGTAGAACAATTTCAGT
TAGATCATGAGACAATTGATATTCATCTCTTTGAGACTCAACATAATTAAAAGCATTTGGAG
GGATAACCAATTGAGAACTCATTATATTAAAAAAAGGCTGCGCAGCAGAATCGACAACTG
GAATTAATAAGTGTAAATGATATGAACACACTAACAAGAATATCAGAAGACAATATGTGT
TGCGCTGTGACGATTAACGAAATATATTGTGGTAATGATGATAGTCAGTACTAACTGGTTTG
TATTTATAGACGGATTGAAGACTGAGTATATACGAGGAAACAATGCATAACGACGTCGTTA
TTTGACGGAATGGCATTCCCCTAAATTAAGATAATGGCATATTTGTAATAATAGTTGGGCAC
CGATTGGGGTCTTCTCAAACTTACTAAAGCAATTGGGGACTGGGGTCTTATATATACTAGAA
CCCTCTATAGAACTTTCAATCTCSTTCACACACGTGGCGGCCATCCGATATAATATT

>A_DF_50_LR_LVV_20_-_MT214086 Tomato chlorotic mottle virus (ToCMoV)- DNA A

ACCGGATGGCCGCGCGATTTTTTTAAGGCCCATTTATTTTGGGCCTCTCTTTTGGGCCTTCAC
CTTTAATTTTATTGAAAGTAATTTTGACCCAATCATGATGCATTTGACGAGCTAAGATATTTA
GAACTACTTAACGCCCAAGTTGCATAACGGCTATAAATTAAACGTACATATGTCGATAGCT
TTAATTCAAAATGCCTAAGCGCGATGCCCCATGGCGCCACATGGCTAGTACATCTAAGATT
AGCCGGGGCGTTAATAATTCTCCACGTGCAGGAGTTGGGCCACGGTCCAACAAGGCCACTG
ACTGGGTAAACAGGCCCATGTACAGGAAGCCCAAGATATATCGGATGTATAGAACCCCCG
ATGTTCCAAGGGGTTGTGAAGGCCCATGTAAAGTGCAGTCGTTTGAACAGCGTCACGATAT
CTCCCATACTGGTAAGGTGATGTGCGTCTCCGATGTGACACGTGGTAACGGTATTACACAC
CGTGTGGGTAAGCGTTTCTGTGTTAAGTCTGTGTATATTCTGGGTAAGATATGGATGGATGA
GAACATCAAGTTGAAGAACCACACGAACAGTGCCATGTTCTGGTTGGTCAGGGACCGTAG
ACCTTATGGTACCCCTATGGACTTTGGCCAGGTGTTTAACATGTTCGACAACGAGCCTAGCA
CCGCCACTGTGAAAAACGATCTCCGTGATCGTTTTCAAGTCATGCATAAGTTCTATGCTAAG
GTCACAGGTGGACAATACGCAAGCAACGAGCAGGCGCTTGTCAAGCGGTTCTGGAAGGTC
AACAATCATGTCGTCTACAACCATCAAGAAGCTGGAAAATACGAGAATCATACGGAGAAC
GCCCTATTATTGTATATGGCATGTACTCATGCCTCAAACCCCGTGTACGCAACTTTAAAAAT
TCGGATCTATTTTTATGATTCGATAACAAATTAATAAATTTTGAATTTTATTGAATGATTCTC
GAGTACAGCATTTACATATCGTCTGTCTGTTGCGAAGCGAACAGCTCTAATTACATTATTAA
CTGAAATAACGCCTAGCTGATCTATGTACAACATAAGTAAATATTTAAATCTATTTAAATAT
GTCATCCCAGAAGCTCTCAGGGATGTCGTCCAGATTTGGAAGTTGAGATAAGCCTTGTGGA
GACCCAGTACTCTCCTCAGGTTGTGGTTGAACCGTATTTGGACGTGGTAGACTCTGGTCCTC
GTGTATAGTATGTCCTCGACGTTGTACATCTTGAAATAAAGGGGATTTGATATTTCCCAAGT
ATACACGCCATTCTCTGCCTGATGTGCAGTGATGAGTTCCCCTGTGCGTGAATCCATGTCCA
GCGCAATCTATGTGTACGAAAATGGAGCAGCCGCACTGTAAATCAATTCGTCTTCTTCGTAT
AGCTCTCCTCTTAGCAGCCCGATGTTGAACTTTGATAGAGGGGGGAGTTGAGGAAGATGAA
TTGTGCATTGTGCTTTGTCCAACTACTTAATGCTGAGTTCTCCTCTTTGTCGAGGAAACATTT
ATAGCTGGCCCCCTCGCCAGGATTGCAAAGCACGATGCATGGGATACCACCTTTAATTTGA
ACTGGCTTTCCGTATTTACAGTTTGATTGCCAATCCCTTTGGGCCCCAATCAATTCTTTCCAG
TGCTTTAACTTTAGATACTGCGGTGCGACATCATCGATGACGTTATACTGCACATCGTTTGA
GTATACCTTTGAATTGAAATCGAGGTGACCACTCAAGTAATTATGGGACCCTAATGCACGT
GCCCACATAGTTTTGCCACTTCGAGAATCACCTTCGATTATTATACTGATAGGTCTTTCCGGC

CGCGCAGCGGCATCTCTCCCGAAATAGTCATCTACCCAATCTTGCATCTCGACAGGCACGTT
AGTGAACGACGAGAGGGGAAACGGAGGAACCCAAGGTTCTGGAGCCTTTGCGAAAATAC
GCTCTAAATTTGATCTAATGTTATGATGTTGAAGCACGAAATCTTTGGGTTGTTCCTCTTTTA
ATATATTGAGTGCTTCCATGACTGATCCTGCGTTGAGAACCTTGGCGTACGTGTCGTTGGCA
GATTGCTGACCTCCTCTAGCTGATCTGCCATCGATCTGGAAAACTCCATGATCAACGAAGTC
TCCGTCTTTCTCCACGTATGATTTGACGTCTGACGAGCTCTTAGCTCCCTGTATGTTTGGATG
GAAATGTGCTGACCTACTTGGGGAGACGAGGTCGAAGAACTTGTTGTTTTTGCAGCTGTATT
TACCTTCGAATTGGATGAGCACGTGGAGATGAGGTTGCCCATCTTCGTGTAGTTCTCTCGAG
ACACGAATGAACAATTTATTTGTTGGTGTGTTCAAGGCTAATAATTGGGAAAGTGCTTCTTC
TTTGGAAAGAGAACAATGTGGATATGTTAGAAAATAATTTTTGGAATTTACTCGGAAATGTT
TGGGCGGTGGCATATTTGTAATAATAGTTGGGCACCGATTGGTGGCTCTCTAAACTTGCTCT
AGCAATTGGGGACTGGGGTCCTATATATACTAGAACCCTCTATAGAACTTTCAATCTCCTTC
ACACACGTGGCGGCCATCCGATATAATATT

>A_DF_166_LR_LVV_16_-_MT214092 Sida micrantha mosaic virus (SiMMV) - DNA A

ACCGGATGGCCGCGCGATTTTTCCCCCCCCCACGTGGCGCGCTGGTGGTCGTGCGATCTCT
CTCCCCCCCTCTCGCGCGACGTGGAGCTCTGGTGTCCGCGGCGTGTCCCCGCCGCGCGCTCT
GCCTTTAATTTAAATTAAAGGAAATAACTTTCATCAGGACCAATGAAATTGCGTCTTTTAAG
CCTAGATATCAGCGTAAGACTTGGGCCCTAAGTTGTTTGACGGCTATATAATTAAGTCATGT
ATGACGTCAGGAATTATTTCGAAATGCCCAAGCGGGATCCCTCATGGCGCCAGATGGCGGG
AACCTCAAAGGTTAGCCGCTCTACCAACTTTTCCCCTCGTGGAGGTGGAGGCCCAAAGTAC
AACAAGGCCTCAGAATGGGTTCACAGGCCCATGTATAGGAAGCCCAGGATATACAGGATG
ATAAGGACTCCTGATGTTCCAAGAGGCTGTGAAGGGCCCTGTAAGGTCCAGTCCTACGAGC
AGCGTCACGACATCTCACATGTCGGGAAGGTCATGTGCATATCTGATGTCACACGTGGCAA
CGGTATTACCCACCGTGTGGGTAAACGTTTTTGTGTCAAGTCTGTGTATATATTAGGCAAGG
TCTGGATGGATGACAACATCAAGTTGAAGAACCACACGAATAGTGTGATGTTCTGGTTAGT
TAGGGACAGGAGACCGTATGGCACTCCCATGGATTTTGGCCAGGTGTTCAACATGTTTGAC
AACGAGCCTAGCACCGCCACTGTGAAAAACGATCTCCGTGATCGTTTTCAAGTCATGCATA
AGTTCTATGCTAAGGTCACAGGTGGACAATACGCAAGCAACGAGCAGGCGCTTGTCAAGC
GGTTCTGGAAGGTCAACAATCATGTCGTCTACAACCATCAAGAAGCTGGAAAATACGAGA
ATCATACGGAGAACGCCCTATTATTGTATATGGCATGTACTCATGCCTCAAACCCCGTGTAT
GCAACTTTAAAAATTCGGATCTATTTTTATGATTCGATAACAAATTAATAAATTTTGAATTTT
ATTGAATGATTCTCGAGTACATAATTTACATAGGCTTTGTCAGTGGCAAAGCGAACAGCTCT
AATTACATTGTTAAGGGAAATTACACCTAATTGTTCTAAGTACATCATTACAAGGCGTCTAA
GCCTAATCAAATAAGTCGTTCCAGAAGCTGTCAGAGAAGTCGTCCAGACTTGGAAGTTCAG
GTAGGCCTTGTGGAGACCCAATGCTTTCCTGAGGTTGTGGTTGAACCTTATCTGTATGTGGT
ACACTCTGGTCCTCGTGTACAGCGGATCCTCTACGTTGTACATCTTGAAATAAAGGGGATTT
GATATCTCCCAAATATAGACGCCATTCTCTGCCTGACGTGCAGTGATGAGTTCCCCTGTGCG
TGAATCCATGTCCTGTGCAGTTGAGATGTTGGTATATGGTGCAGCCGCACTCCAGGTCCACT
CGTCTACGCCTGATTGCTCTCTTCTTGGCAATCCTGTGTCTCGGTTTGATAGAGGGGGGCGTC
GAGGAAGATGAATTTAGCATTGTGAAGTGTCCAGGCTTTTAGTGATGCATTTTCCTCTCTCTC
GAGGAATGCTTTATAACTGGCCCCCTCCCCAGGATTGCACAGCACGATTGAAGGGACCCCA
CCTTCAATCAAACGAGGCTTTCCGTACTTGCAGTTTGTCTGCCATTTCTGTTGGGCCCCTATT
AGCTCTTTCCAGTGTTTCAACTTTAGGTAATGCGGAGCGACGTCATCAATGACGTTATACTC
CACTTGATCAGAATATACCTTTGAGTTGAAATCCAGATGGCCACTGAAGTAATTATGTGGG
CCTAATGCTCTAGCCCACATCGTCTTTCCCGTTCGAGAATCACCTTCAATGATGATACTAAT

AGGTCGTTCCGGCCGCGCAGCGGCACCTCTCCCAAAATAGTCATCTGCCCAAGCTTGCATC
TCTCTCGGTACGCTAGTGAAAGAGGAGAGGGGAAACGTAGGGGCCCATGGCTCCGGAGCC
CTTGTAAATATCCTATCTAAATTACTATTTAGATTGTGAAACTGAAATAAGAACTTTTCAGG
CAGCTTCTCACGGATTATCTGCAAGGCGACGTCTTTGGAAGGTGCGTTCAAGGCTTCTGCGG
CAGCGTCGTTAGCTGTCTGGCAACCGCCTCTAGCACTTCTTCCGTCGATTTGGAATTCCCCC
CACTCGATAGTATCTCCGTCCTTGTCGACATAGGATTTGACGTCGGACGATGACTTAGCTCT
CTGAATGTTCGGATGGAAATGTGTTGACCTTGTTGGGGAGACCAGGTCGAAGAATCGTTGA
TTCGTGCATTGGAATTTCCCTTGGAACTGGATAAGGACGTGGAGATGAGGCTCCCCATTTTC
GTGGAGCTCTCTGCAGATTTTGATGAACTTCTTGTTAACTGGTGTTGCTAAGCTCTGTAATTG
GGAAAGTGTCTCCTCTTTGGTTAAGGAGCACTGGGGATATGTCAAGAAATAATTCTTGGCA
CTTATTTTAAAACGCTTTGGCGGTGGCATTTTTGTAAATAAGGGGTGTACCCCAATTGAGAG
CTCGCTCTATAAGTCATATGAATTGGGGTAATGGGGACAATATATAGTAGAAGATCCTAAG
GGGCTTTAAGCGGCCATCCGCACTAATATT

>GO_298_LR_LVV_17_-_MT214094 Plant associated genomovirus 12

TAATGTTATAGCCCAGGTGACGGGTGACAACCCTTATAAAGAGGCCGCCCCACCTCGCCCC
ACGAAGACCCCACCTCAACACTTTCTCCCCACATGCGCTATGCCTCTCCGTCGGTACAAAG
GGCGCAACAGCGTAAGCTACAAGCGCCGTTCAAAATCCCGCAAGCCAGCCCCAAAGCGGA
GAAAGTATGCGAAGCGCAGGCCGATGACACGGAAGCGAGTCCTCAACATCGCGTCTGTGA
AAAAAGCGGACACAATGGGAACGACCTCTGTTCGTGCCGATGGTACGACATTCAGGGAGC
CATTTGTCATGAACGGCGACAAAACGTATATGATTCCCTGGATTGCGACTTGGCGCAACTTC
CAGACCAGCTCAACAAACAGCACTCGGGGTCTCCGACAGGACCCCACGAGGAGCGCCACG
TCATGCTACATGAGGGGCCTCAAAGAAAATATCCAACTCGGGACGAACGATGGCCAGTGT
TGGCAGTGGAGGCGCATATGCTTCACAATGAAGGGCGACGACATCTACAGCAACCAGAGC
ACCCAGTTCAGGATCGCATTCCAGTCGAGCGCGGGGTACCAACGCGTGTTCTGCGATTGGA
ACAACGACAAGACCAACGGGAGCACGACTTATCCAGCCCCTAGCGTTATGCTCGCGGGCC
CTGTCTTCAAGGGCACGTTGGGTCAGGATTGGGACGATCCATTCATCGCACCAGTGGACCC
ACTGCGTGTGACTCTAAAATACGACAAGACGCGCTATCTACGTTCCGGAAACAATTTCGGA
ATGTTGAAGACAACCAGGCTGTGGCACCCTATGAACGCCACACTCCACTACGACGACGAT
GAAAATGGCGGTGTAGAAGACGGTGCAACCGTATCGGTGAAAACGCATGGAATGGGAGAT
TACTACGTAATTGACTTGTTTCGTGCAGCCACAGGTGCAGTAGCAGGCAGCCAACTGAATC
TGCGAAGCACCTCTACTCTGTATTGGCATGAAAAATAGGCTCCTGTAGCTCCACAAAAATA
CAGTTTGCTTCCAACCAATCAACATCGCTCTGAATCAAATCTCGCTTACTTGCAGCTGACCG
GTCAGACAACTCAGCCCACATCTCTAGACGTGGGTCTCTGTTAGCCAACCAGATGCAAGGT
CTTCCCCATACAAGTTGTACGGGATCTCTGTACAGCTCTTTGACTGTGACCACACTCTGTGC
CCCAAGCCACTCTTTCCATGAATGGAAATATCCAATACCACCTCGCATATCGTCGAAGACA
GCGTACGCAGCTCCCGGTGCATCACGCAGGAGAAGCTTGCCGGACACGATGCCCATACTGT
ACACGTGTGGGCCCAGTGATCTAGCCCAAACAGTCTTTCCAAGTCGTGAAGGACCATAGAG
TACAAGAGACCGCATTCTGCCTTGCTTAGTCAGCATTGCACACACTCACGTAGCGGTCCGA
GCGGCGGAGCGAGATCGGACCTTGCGGAACCGAGCACTACGCTGGCAAAAACCCCTAGGA
GACTATAGACCTCCCGAGAGAGCCCCGCGACCCAGAGGGGAGCGTAGGGCGATTGAGACG
TACCTTCTACTGGATCATTTGCAACAAGCTGTTCTCCCCATCGAGCCAGCTCAGGTACCATT
CCAAGCTCAAATTGCTTCCCATCGGAACCCACATAGGGTTCTGGGGTGGGGGCGTAGGCGG
CGTCGGCATACTTGTTGAGCTCGGTCCAACGAGTGACAAGTGTCTTCGGATCCAGCTGTCGA
AGCAGGTCAAAAAACTCGTCTCGACTTTCTGCCGAGACAATCTCGCGCCATTTATCCGCAA
CTTCAGGTAATCCACCTCCGCCCGGCCTCGCCAACCCCCCTGCAACAACGTTTCCATCTTTG

ACTGCATAGTCGTAACCGAGCTCCGGACGACCTCGAGATGGCGTAATGTTCGGGTGGTGGC
CTCTGACATCAAAGAAATTGGGTCGTCGGGATTGCTTCTTCTTTCCGAAATCCAGAAAAAC
ATGGAGGTGAGTACCTCCATCAGCGTGATCTTCTCGTCCGATGATACACTCAGCTCCAAGC
GCTGACATATGGTCTGAAACGGACCACTCATCAAGGTCTCCACACTGAGCGTAAGTGAGGA
GTGCATAGCGTGCCGAGAATAGAAAAGTCATGTGATAAACCTGAGGAACCTGGGCAAAC

>DF_62_LR_LVV_12_-_MT214093 New world bipartite associated alphasatellite

ACCCCGCTTCGCCACAACTCTTCGCCAAAGGGACCCACTCTAGCAATATAAATATGACTAT
TTTGTGCCTATTGCCTTCATAATCCAAATGACACAAAGTCGAAATTGGTGCTTTACTCTCTTT
GCATACACCCTCCCTCTCTTTGACTCTCTCCCCGAGTGGGCTAATTATATAGTTTTCCAGGAA
GAGGAGTGTCCTACGACCAAGAAGAGACACCTACAGGGGTTCGTTACTCTCAAGAAGAGT
CAAAGGCTATCCTTTCTGAAAGGCAGATTAGGTAATGGCGTCCATCTCGAGATCTCTCGGG
GATCCTCATCCAGCAATAGAGATTATTGCAGAAAGGATGATTCAAGAGTCGCTGGGCCATG
GGAATTCGGTGTACTTGCAGAGCAGGGGAGCAATAAGAGGAAGACGATGGAACGTTTTCA
GGAAGACCCAGAGGAGGTCCGACTGGCCGACCCTAAGTTATATCGTCGCTGCCTGGCGAC
AAAGATTAATAAGGAGTTCCGTGGTCTGGTACTCCCTGCATTTGACAGACCTTGGCAGCTGC
TGGTTGAGAAGATATTGGACAAGGGCCCAGATGACAGAACTATCTTATGGGTGTATGGCTC
TCAAGGCAATGAAGGCAAGACGACCTGGGCCAAGTCTAAGATTCAGGCCGGCTGGTTCTA
CTCCAGAGGAGGAAAGGGAGAGAACATCAAGTACTCGTACGCCGAGCACCTAGGTCATGT
CATATTTGATCTGCCCAGACAGGTTGAAGATGTGCTCCAATACTCGGTTCTGGAGGAAATTA
AGGACAGGTTAATTAGGAGTGGTAAATACGAGCCTATAGATTTTAATTGTAGTGACAAGGT
TCACATTGTCGTTTTAGCTAATTTCCTCCCATCGTTAGATGTTGAGCACGATAATAAGGGAA
ATGTAATTAAGCGCCAACTGTTAAGCAGAGACAGGGTATGTATAGTTAACATTGACGAGTC
TGTTGTTGTACGAGAAGAAGAAACGGTGACGTTTGCTCGTTATTTTGAATAAAAGAAAAAA
ACTTGTTTTTTGTACAGAAAAAAAAGGACTCCGCCTGGTGTCCTGGCTTATATTTACAAATT
TGCCATCACCCACTAACAACTATGAAAAGAAGGGACCGCAGGGACCACAACCAAAAAAA
AAGAAAAGAAAACCACTAACCAAACAGGGATCGATGACGTGGACAACAACAGTTTTTGTT
ACCGGTGACGTGTCAGTCATATGTGTTAGTTACCGGTTCTATTGCCTTGTAAGGTGACCGGC
TACCGTGGCGAGCTACCGGTAAGAAGCGGGTATAGTATT

>B_MG_016_LR_LVV_06_MN746970 Euphorbia yellow mosaic virus DNA-B

ACCGGATGGCCGCGGTTTTTTGGTGTCCTCTATCCCCGGCCCAGGCCCAAACGCATTGTATT
TTGTCCGGGACCACACACAGCAACCAAAGCTGACCAATCAGACTGCGCGTGACGTGTCTAT
TTAAATACAACTTACTAGACAAGTTGTGGTCCCTATAAATGTAGATGACAACGACAGCACA
TCAACTCTTTAATTTGAATTAAGCCCACTTTGTATGGCGGGAGCCTTCAGTCGTTGTGGTCCT
CAACCACAATTCGAAATAAGCTGTCGTCAAGCCGACGTACATCCGAGTCGCTCGAGAGCTC
CACGGTCCAATCATAAACGTTGTACATAGTCTCGTTAACCCAACCGAATTTGTGGACTGGTT
ATTTAAGGACGATATGGATTGGACCTCATATCATATCATCTCGATAACCATTTTATGAAATG
TATACGACTAAGTATAGACGTGGTGGGTCATCTCTTAAGCGACGAGGTTATTCACGACATC
CAGCGTTTAAGCGAATGCAGAATGGAAAACGTTCCGACTGGAGACGTCGACACAGCAGTC
CTTGTAAGGCCCATGACGATACTAAGTGGACGGCCCAATGTATACATGAAGATCAGTTCGG
CCCAGAATTTGTATTGGGCCATAATATAGCTACGTCGACCTATATTAGCTATCCTAGTATGA
GTAAGACTGAACCGAACCGATCCCGATCGTATATTAAGTTGAGGCGTCTACGCTTTAAGGG
CACTCTGAAGGTCGAGCGTGTACACGTCGATGTCAACATGATCGGTTCGAATCCAAAGATA
GAAGGCGTATTCTCTATGGTCGTTATTGTCGACCGTAAACCTCACTTGGGTCCAACTGGATG

CCTTCACACATTTGATGAGGTATTTGGCGCAAGGATCCACAGTCATGGTAATCTAACCATCA
GTCCTTCTTTGAAGGATCGGTACTACATCCGCCATGTATTCAAGCGCGTCATATCTGTCGAG
AAAGACACGATGATGGTGGATATAGAAGGGTCGACGTGTTTATCCACTAGGCGTTCTTCTT
GCTGGGCCACTTTTAAGGATACGGAACGAGAGTCATGTAATGGTGTATATGCGAATATAAG
CAAGAACGCCATATTAGTGTATTATTGCTGGATGTCGGATCATATGTCCAAGGCATCTACAT
TTGTATCATTTGACCTCGATTATGTCGGATGACCAATAAGAACATATTTAACATGATATTAA
TCAAATAGCTTCATCGTACATGCAAATGATAACAACCTCAATAATTGAATTAAATAACGTA
TTATTTAATCAAGCGTTTTATTTCTGGGAGGAATACAATTAGTGCTAATACATTCCTGAACG
GTTGACCTAACTATCTCGTTCAGCTGACTCATTGACATCGATATGTTCGAATGTGTTCTCTGA
GCAGCTAGTACGGAAGCTGAATCTCCCGGGTCTAATACGGGAGTCCCTGGCCTATTTAACT
CCCGATATGGATAAGCGGCGTCTCTCACATCTGAATCCGCATCTAAATGGCCCAGTCCTAT
GTTGGATCTGGTGGCCCATGATTCACCAGGCCTTATTTCGATTGGGCCTTGTAGCCCAAGTC
TCGATGATGATGCGGATCTAATCATTCTTCTCTCCCATTTGCCGTAGCCCACATGACTGAAG
TCGATATCCTTATCTGAAAACTGTTTGGACAGGATCTTGACAGTTGGCGCTCTGAAAGGGAT
ATCAACGGAATGTTTTGCCGTCGATAACTTCAGTTTACCCTTGAACTTCGCAAAATGCGTCA
TCTGATGAACGTTTGAGTCAGAAACCTTATAATATAGCTTCCATGGAATTGGGTCTTTGAGT
GAGAAGAATGATGATGAGAAATAGTGGAGATCTATGTTGCATCTGATGGGAAATGTCCATG
ACGCCTGTAAGGATTCATTTTCAGTCATTCTCTTGTCGTGAATCTCCACTACTACCGAGCCCG
ACGCATTAATTGGCACCTGTTGCCTATACTCTATTACGCAATGGTCGATCTTCATGCAACTG
CGTGAGAGTCGGGCTCCTAATTGAGACGCCGTTGAGGGGAATTGCAAGACTATCTCAGTTA
GGTCGTGCGATAGCTGATACTCGTCACGATGTGACTCTATGTAATTGAAAGCGCTCGGCGG
ATTTACTAACTGAGACCCCTCCATTATATTATGGAAACTTGCCCGCGCAGCGGCACCCCTTC
CCCCAAATTGAACGACTGAGGGAAATTGATAGGTGCCAACCTTATGGAAGGACGATATGC
GTTAGCGATAACAATTTGGGGAAATTTCGAAGATACGATAAGCCTATGTGCAGATTATGAT
TTAATCGGCTGGTAATCTGAGATGAAGAAGTGGAAATTTGCCTATATGTTAGCTATTGAAG
GTCTATATATAGGCAGTCAGATGCGATATGCAACAGGCTGATGGCAAATTTGTAAATAGAA
GTCAGGACACCAGGGGGAGTCCTCTCTAAAACCTATTTGTGTTGGTGTCCTGGTGTCCCATT
TATACTAAAACCCTCTAGGACACTGGGGGGTACGTGGCGGCCATCCGTTAATATT

>A_MG_016_LR_LVV_06_MN746971 Euphorbia yellow mosaic virus DNA-A

ACCGGATGGCCGCGATTTTTTGGTGTCCTCTATCCCGGCCCAGGCCCAAACGCATTTAGTTT
TATCTGGGACCACACACAGCAACTAAAGCTGACCAATAACATCACTGCTGACGAGGCTAT
ATAACTTTGAGCCTACTTGGCCGATAAGTTGTGGTCCCTATATATTTAAAGTAATTGACATT
ATTCGATATCTTTAATTTGAAATGCCTAAGCGAGATGCCCCATGGCGATTAATGGCGGGGA
CCTCCAAGGTCAGTCGTAATGTCAATTATTCGCCCCGTGGTGGTCCTAAAATAGACAAGGC
CTCTTCATGGGTGAACAGGCCCATGTACAGGAAGCCCAGGATCTATCGCATGTATAGAGGC
CCAGATGTTCCGAAGGGATGTGAAGGGCCTTGTAAGGTCCAGTCATACGAGTCTCGTCATG
ACGTTTCCCATGTTGGGAAAGTGATATGTGTATCCGACGTGACACGGGGCAATGGTATTAC
GCATCGTGTGGGCAAGCGATTCTGCGTCAAGTCTGTGTACATACTAGGGAAGATCTGGATG
GATGAGACCATCAAGTTGAAGAACCACACGAATAGCGTCATGTTCTGGTTGGTGCGAGATC
GTCGACCATATAGCTCTCCTATGGATTTTGGCCAAGTGTTCAACATGTTCGACAACGAACCC
AGTACAGCCACTGTGAAGAACGATCTCCGCGATCGCTTCCAAGTGATGCACAAGTTTTACG
CGAAGGTCACTGGTGGACAATATGCGAGCAACGAGCAGGCGATCGTCAAGCGTTTTTGGA
AGGTCAACAACTATGTGGTGTATAACCACCAAGAGGCAGGGAAATACGAGAATCATACGG
AGAACGCGTTACTATTGTACATGGCATGTACTCATGCCTCTAATCCTGTATATGCGACGTTG
AAAATTCGGATCTATTTTTATGATTCGATAACAAATTAATAAAAATTGAATTTTATTTCATG

ATTCTCGAGTACATCATTTACATATGAGCGATCAGTTGCGAAACGAACAGCCCTAATTACA
TGATTAAGACCGACGACACCCAATTGATCTAAATACATTAAAACTAAATGCCTGAATCTAC
TTAAATAAGTCATCCCAGAAGCTCGAATCGATGTCGTCCATACTTGGAAGTTCAGGAAAGC
TTTGCGTAGATTCAGTGCCCTCCTGACGTTGTAATTGAACCGGATCTGGATGTGGTATATCC
GGAGCGACGTGTAAATCGGGTCCTCGACCCTGATGATCTTGAAATAGAGGGGATTTGGAAC
CTCCCAAATAAAAACGGAATTCTCTGCCTGAGACACAGTGATGAACTCCCCGGTGCTTAAA
TCCATAATCTGCACAGTCGATATGGAGAAATATGGAACAGCCGCAGTTCAAGTCAATCCGT
CGTCGACGTATAGCTCTCCTCTTGGCTATTCGGTGCTGTGCTTTGATAGAGGGGGGCGTGGA
GGAAGATGAATTTAGCATTCTTGCGTGTCCAAGCTCTCAACGATGCATTCTCCTCTTTGTTGA
GGAAGTCTTTATAGCTAGCCCCCTCGCCTGGATTGCACAGCACGATTGATGGTATCCCGCCT
TTAATTTGAACCGGCTTTCCATATTTACAGTTGGACTGCCAGTCCCTTTGGGCCCCAATCAA
CTCTTTCCAGTGCTTTAACTTTAGATAATGCGGAGCGACATCATCAATGACGTTGTACTCCA
CGTCATTTGAGTAAACCCTAGAATTGAAATCGAGATGCCCACTAAGATAATTATGTGGGCC
TAAAGCTCTAGCCCACATGGTCTTACCCGTTCGACTATCCCCCTCGACTACGATACTAATAG
GTCTATCTGGCCGCGCAGCGGCACCCCTTCCAAAATAATCATCAGCCCACTCTTGCAAATC
AGACGGGACATTGTTAAATGACGACAGCTGAAATGGGGGAACCCAAGGAAGGGGTGGCTT
AATAAAGAGGCGCTCGACGTTAGCTTTGACATTATGATAATTCACGATGAACGTCTTCGGA
TCACCAGCCCTTATTATGTCGAGAGCCTCTCCTGCAGTTGCTGCATTGACAGCGTTGTGGTA
GACGTCATCTTTATTGGACTTGGAACCCCCAGACACCTTATATTTGCCGGATTCACAATAAT
CACCCTCTTTGGTGATGTAATTTTTGACGGCGTTGGCGTCCTTGGCCGATTGCGCATTTGGGT
GAAATTCGGCAGACCGTCTGGGGTGAGTGAGGTTGAAAAACCTAGCATCCTTGATGTTGGA
CTTGCCGGAGAGTTGAATAAGACAGTGGAGATGGGGGAACCCATCGGCGTGGAGCTCTCG
AGCGACTCGGATGTACGTCGGCTTGACGACAGTCCATGGCAAGTCCCGAAGCAACTGCAG
AACTTCATCTTTCGGAATATCGCACCGGGGATAAGTGAGGAATATATTTTTGGCCGTTAGGC
GAAATGAATTAGGGTTCCGTGGCATAATTGAAAATATAAGCCAGGACACCAGTGGGAGTC
CTCTCAAAAACCTAATTGTCGTGGTGTCCTGGTGTCCCATTTATACTAAAAGCCTCTAGGAC
ACCAGGGGGTACGTGGCGGCCATCCGTATAATATT

>B_MG-150_LR_LVV_01_-_MN337872 Cleome leaf crumple virus (ClLCrV) - DNA B

ACCGGATGGCCGCTGGATCTGTTTTATGTTCGGCCCGCGCAAGCGGAAGAGTTGGGCCGAC
GATGGAAAAAAACTCGGCCCAGATAAGAAAAGGGATAGGATTTCGCTCTCCGCGAAATAA
AAGAGACCGCATTGGAAGGCGCGTGTGGTCCTCGATGACATGGCAATAACGGATGGCCAA
TGGGAGTGGCGCTTCTCGGCAATTTAAAGGGGTACGATTTGGGACCACATGCAATTGCGAC
GTGACATATTTATCAGTATAGCGCATGCTGTGGTACAGGATGCATAAGGCGAGTTGAACTG
GCAATTTGAATTTGAGCACGTCGCCCTTTCTGTGGTCACGGACCACATTCGAAAACCAATCG
AGCTCCGACGTCAAGTCCTACGTCGACAAAGACGGGGATACTCTCGAGGAACACGTGGAC
CACTGGGTTTTCGAATAGGAATTCTAGTTATTCAGATATGGGTTTTGGACGCTTTATATAATG
CGCCAGTTGACAAAGAGTATGCCATCATTAGGTATTTTGACAGGCATATCTGAATATGTATT
CCAGTAAATACAGACGTGGTGAATCGTCCACTCAGAGGCGAAGATATTCACGCTATCAAGC
ATCTAGACGCTCACACGGTGTGAAGCGTAGTGCTGGGAGACGTCGAGGTATAGTTTCTTTG
AAGAGCCGTGATGAAGGCAGGATGCACTCACAGTGCATTCACGAGGACCAATTTGGTCCG
GAATTCGTTATGGGGCATAATAATGCCATTTCAAGCTTCATCACGTACCCCACTCTCGGTAA
TGTTGACCCATGCCGCAGCAGGTCCTATATTAAGTTAAAACGCTTACGTTTTAAAGGAACCC
TCAAGATTGAACGAGTTCATGCGGATGTCAACATGGACGGTAGAGCACCGAAGATTGAGG
GAGTGTTTTCACTTGTTGTGGTGGTCGATCGGCGACCACATTTAAGCCCCACTGGACGCCTG
CATACGTTTGACGAGATGTTCGGTGCCAGGATCCATAGTCACGGGAACTTATCCATCGTTCC

CGCTCTGAAAGACCGGTTTTACGTGCGGCACGTTTTGAAACGTGTATTGTCGGTCGAGAAG
GACTCTCTGATGGTGGATATAGAAGGGACGACCGTCTTGTCTAGCAAGCGGTTTAGCTGTTG
GTCATCGTTTAGGGATCTCGAGAGAGATTCATGTAATGGAGTCTATGCTAATATTAGCAAG
AACTCCCTTTTAGTTTACTACTGCTGGATGTCGGACGCGATTTCCAAGGCGTCGACATTTGT
ATCATATGATCTGGACTATGTTGGCTGAATAAAGTGATTAATGATGTTTATGAAGATAAACA
TGTGACAACATATAGAGGATAATCTGAATTACAGGCACGATAACTATTCTAAAGGCTTGGT
ATGCGAAGGGACACAGTTTGTATTTATGCATTCCTGGACCGTCGTCTTTACTATCTCGTTGA
GTTGGGCTAGCGACAGCGTTATGTTGGACTGTGTCCGCTGAGCCCCAAGTACAGAGGCAGA
CTCTCCTGGGTCCAGGATTGGTGTACCCAATCTGTCTAGGTGCCTGTATGGATGCAGCGCAT
CCCTCCGTTCAGAATCCGCATCGGTATGGGCCGAGTCGATAGCACTTTTGACGGCCCAAGA
CTCTCCTGGTTGTATTTCGATTGGGCCGCCCAACCCATAATGTGACGACGATGCGGATCTCG
CCATTCTTCTCTCCCACTTGCCGTACCCCACGTGGCAGAAATCGATATCCTTCTCGGTGAAC
TGCTTCGACAAGATCTTGACGGTCGGAGCCCTGAACGGGATATCAACGGAGTGCTTCGCCG
TTGATAGTTTCAGCTTCCCCTTGAATTTGGCGAAGTGTGTCTGCTGGTGCACATTCGAATCA
GAAACTCTGTAATAGAGTTTCCATGGGATTGGGTCTTTGAGAGAGAAGAATGAAGAGGAG
AAATAGTGGAGATCGATGTTGCATCTGATCGGGAAAGTCCACGACGCTTGCAGCGACTCGT
TGTCAGTCATCCTCTTGTCGTGGATCTCCACTATCACCGCGCCCGACGCGTTGATGGGAACC
TGCTGCCTGTATTCTATGACGCAGTGGTCTATTTTCATACAGCTCCGAGTGAGTCGGGCGCT
AATCTGTGACGCCGCCGACGGGAATTGGAGGACGATCTCGGTCAGGTCATGCGAAAGCTG
ATACTCATCGCGATGGGATTCGATATAGTTGAATGCGTTCGGCGGCGCAACCAGCTTCGATT
CCATTCCCCCTAGCCGTCGTTGTCCGGTCAACCAGGTTGAGCTAGAGATGATTGATCAATTG
CGATTTAGGAAAGCAAAACGAGAATGGAATCGTGGAGTGAGATGTTAATTGACAGGTTTCT
GCGAGAGAGGAGATATTGAAAGAAGAGGACAGTGTTTCAGCGCTTAATCTGTCTTCGTTTA
TATAGACGATTAAGCAAAAGGAGAACTGAATTGAGGTCCGATATATAGCGCCGATGGCAT
TTTTGTAAATAAGAGATGAGTACCGATAGGAGGGAGGTGAGTACCGATTGGGGAGCTCTCA
ACTTGGTGAAATGAATCGGGGAATGGTACTCAATTTATAGCAGAGTCCGTTAGGGTATATTT
GCCACGTGTCGGCCATCCGTATAATATT

>A_MG-150_LR__LVV_01_-_MN337873 Cleome leaf crumple virus (ClLCrV) - DNA A

ACCGGATGGCCGCTGGATCTGTTTTATGTTCGGCCCGCGCAAGCGGGAGAGTTGGGCCGAC
GATGACAAAAACTCGGCCCAGATAAGAAAAGATGATAAGGGGACCACGTCCCAAAAAGG
AACATATAGATGGGACCACATAAAGACAATGATTACGCCCAATAAGGATTGGGCAACAGA
GCCAAGATATTCAAATATACTTGGCCGATAAGTTGATAAAGCTGTATAAATGGTCCCATCA
CCCTTCGTGGTCTAGTGAGCCCCGACTAGGACTCGACGTTCTGTGGTGCAGAATGCCCAAG
CGGGATGCCCCATGGCGTACGCCTTCAGGGACCACGAAGGTTAGTCGCAACGCCAACTACT
CCCCTCGTGGAGGCACGGGCTCAAGACTAAGCAGGGCCACTGCTTGGGTTAATCGGCCCAT
GTACAGGAAGCCCAGGATATATCGGATGTATAGGTCACCCGATGTGCCCAGAGGGTGTGA
AGGCCCGTGCAAGGTCCAGTCTTACGAGCAGCGCCACGACATATCTCATGTCGGCAAGGTG
ATGTGCGTGTCTGACATTACCCGCGGTAACGGTATTACCCACCGCGTGGGTAAACGTTTTTG
TGTCAAGTCTGTGTATATATTAGGCAAGGTCTGGATGGACGACAACATCAAGTTGAAGAAC
CACACGAATAGTGTGATGTTCTGGTTAGTTAGGGACAGGAGACCGTATGGCACTCCCATGG
ATTTTGGCCAGGTTTTTAACCTGTTTGACAACGAGCCAAGCACCGCTACGGTGAAGAACGA
TCTTCGTGATCGTTTTCAGGTGCTGAACAGGTTCCACGTGAAGGTCACTGGTGGACAGTACG
CGAGCAACGAGCAGGCGCTGGTTAGGCGCTTTTGGAGGGTCAACAACCATGTGGTGTACA
ACCATCAGGAGGCTGCGAAATACGAGAACCATACGGAGAACGCTCTATTATTGTATATGGC
ATGTACTCATGCCTCTAACCCTGTATATGCTACTTTAAAAATTCGGATCTATTTTTATGATTC

GATAACAAATTAATAAAGTTTGAATTGTATTGAATGGTTCTCGCGTACACGTCTTACATACG
GTCTGTCCGTTGCGAAGGTGACAGCTCGGATTACATTGTTAAGGCTAATAACGCCCACGTTA
TTTAGATACATGAGGACCAAATGTTTAAACCTATTTAAATAGGTCTGACCAGAAGCTGTCA
CCGATACTGTCCAGACTTGGAAGTTGAGGAACGCCTTGTGGAGATCCAACGCTTTCCTCAG
GTTGTGGTTGAACCGGATCTGGACGTGGTAGACCCGCGTCGTTCTGGACAGGATCTCCACC
ACGTTGTACATCTTGAAATAAAGGGGATTTGTTATTTCCCAAGTATACACGCCATTCTCTGC
CTGATGTGCAGTGATGAGTTCCCCTGTGCGTGAATCCATTATTCGCGCAGTTTAGGTGGATG
TATATTGTACATCCACAGTTCAGGTCGATACGTCTACGGCGAATGGCTCTCCGTTTCGCCAC
TCTGTGTTGCACCTTGATAGAGGGGAGATTCGAGGAAGACGAACCGCGCATTGTGAAGGGT
CCACGACCGGAGTGCAGCATTCTCCTCTTTCTCGAGGAAGTCTTTATAGCTGGCCCCCTCTC
CTGGATTGCAAAGCACGATTGATGGGATACCGCCTTTAATTTGAACGGGTTTCCCGTATTTG
CAGTTACTTTGCCAGTCTCGTTGGGCCCCAACGAGTTCTTTCCAGTGCTTTAGCTTTAGGTAG
TGCGGGGCAACGTCATCGATGACGTTGTATGCGACCTCGTTTGAGTAGACCCTCGAGTTGA
AGTCGAGGTGTCCACTGAGATAATTGTGGGGACCTAAAGCACGTGCCCACATCGTCTTCCC
CGTCCTCGAATCTCCTTCGACGATCAGACTTATTGGTCTGTTCGGCCGCGCTTCGGCGGATA
AGGCGAAATACTCATCTGCCCACGCCTGCATTTCGTCCGGAACGTTAGTGAATGAGGACAA
CGGGAACGGAGGGACCCATGGTTCCGGAGCCTTGGCGAAGATTTTCTGGACATGTGCTTGC
ACCTTGTCCAAATGGAGGACGAAATCCCGCGGTTGCTCCTCCCTTAGAATCTGCAGTGCTTG
CGCTGCAGACTCGGCGTTAAGGACCTTGGCGTATGTGTCGTTAGCCGATTGGCAACCGCCTC
TAGCACTTCTCCCGTCGATCTGGAATGTTCCCCATTCCAGAGTATCCCCGTCTTTGTCGACGT
AGGACTTGACGTCGGAGCTCGACTTAGCTCCCTGAACGTTCGGATGGAAATGTGCTGACCG
GGACGGGGATACCAGGTCGAAGAATCTTTGATTCTGGCATTTGAACTTCCCCTCGAACTGG
AGAAGTACGTGGAGATGAGGCTCCCCATTCTCGTGGAGCTCACGGGCGATCTTGATGAATT
TCTTGTTCGTCGGCGTCGATAGGGCTTGCAATTGGGAAAGTGCTTCCTCTTTGGTCAGGGAG
CACTTTGGATAAGTGAGGAAATAATTTTTGGCGTTTATTTGGAAACGCTTTGGCGCCGATGG
CATTTTGGTAAATAAGTGGTGAGTACCGATAGGAGGGAGGTGAGTACCGATTGGGGAGCTC
TCAACTTGGTGAAATGAATCGGGGAATGGTACTCAATTTATAGCAGAGTCCGTTAGGGTAT
ATTTGCCACGTGTCGGCCATCCGTATAATATT

>A_DF_46_LR_LVV_13_-_MT214083_ Bean golden mosaic virus (BGMV) - DNA A

ACCGGATGGCCGCGCGATTTTTTTAAGGCCCATTCATTTTGGGCCGCTTGGGCCGTTCTTTA
TTTGAATTAAAGTAAAGTCATTTATTCTGACCAATAACTTTAGTTCTGACGGGTTTAGTTAAT
TTTGAACTTGGCGACCAAGTTCTTTACTAAACTATAAATTTAATCTTGTATTGACCATGTTCT
TTAATTCAAAATGCCTAAGCGTGATGCTCAATGGCGCCACATGGCAGGTACGTCAAAAATT
AGCCGTTCGGCTAATTTCTCTCCTCGTGGGGGAAATGGGCCTAAATACAACAAGGCCGCTG
AATGGGTTAACAGGCCTATGTATAGGAAGCCCAGGATATATCGAACTATACGAGGCCCTG
ATGTTCCACGGGGCTGTGAAGGCCCGTGCAAGGTTCAGTCTTATGAACAACGACATGATGT
GTCACATGTTGGGAAGGTTATGTGTGTATCTGATGTAACAAGAGGTAATGGTATTACCCATC
GTGTAGGAAAACGTTTTTGTGTTAAGTCTGTTTATATTTTAGGTAAGATATGGATGGACGAG
AATATCAAGTTGAAGAATCACACGAACAGTGTGATGTTTTGGTTAGTCAGAGATCGTAGAC
CGTATGGTACTCCTATGGACTTTGGTCAAGTTTTTAATATGTTCGACAATGAACCCAGTACT
GCTACTGTTAAGAACGATCTTCGTGATCGTTTTCAAGTTATGCATAAATTCTATGGAAAGGT
GACAGGTGGACAATATGCTAGCAACGAACAGGCATTGGTCAAACGTTTTTGGAAGGTGAA
TAATTATGTAGTCTACAACCATCAAGAAGCAGGGAAGTACGAGAATCATACAGAGAACGC
ATTATTGTTGTATATGGCATGTACTCATGCCTCAAATCCAGTGTATGCTACTCTAAAAATTCG
GATCTATTTTTATGATTCGATCACCAATTAATAAAATTTATATTTTATTTCATGATTCTCAAGT

ACATAATTTACATATGATTTGTCTGTTGCGAAACGTACAGCTCTGATAACATTGTTAAGCGA
AATAACACCTATTCTATCTAAATACAACAGCACTAAATATTTAAATCTATTTAAATATGTCG
TCCCAGAAGCTTGAAGAGATATCGTCCAGACTTGGAAATTCAGGAAAGCCTTGTGTAGATC
CAGTTTGTTCCTGAGGTTGTGGTTGAACCGTATTTGGAGGTGGTATACTCTGGTCGTTGTGTA
TTGTAGATCCTCTACGTTGTATATCTTGAAATAAAGGGGATTTGATATCTCCCAGATATACA
CGCCATTCTCCGCTTGACGTGCAGTGATGCGTTCCCCTGTGCGTGAATCCATGACCGTAGCA
ATTTATGTGTACATAAATTGTGCAGCCGCACTCTAAATCAACGCGACGTCGTCTGATTGCTC
GTTTCTTCGCAGCTCTATGTTGAACTTTGATAGAGGGGGGAGTCGAGGAAGATGAATTTCGC
ATTATGTATTGTCCAGTTTTTGAGAGCTGCGTTTTCCTCTTTGTCGAGGAAACATTTATAACT
CGCTCCTTCACCTGGATTGCAAAGCACGATTGATGGGATTCCACCTTTAATTTGAACGGGTT
TCCCGTATTTGCAGTTTGATTGCCAGTCCTTTTGAGCTCCCATCAATTCCTTCCAGTGCTTTA
ACTTTAGATAGTGCGGTGCGATGTCATCAATGACGTTATACTCAGCATGATTTGAAAACAC
CTTTGAGTTGAAATCCAGGTGACCACTTAGATAATTGTGGGTCCCTAATGCACGTGCCCACA
TTGTCTTTCCCGTTCGTGAATCACCTTCGACGATGATACTAATAGGTCTCTCCGGCCGCGCA
GCGGCACCTCTCCCGAAATAATCATCTGCCCATTCTTGCATCTCTCTTGGCACGTTAGTGAA
AGAGGAGAGGTGAAACGGAGGTGACCACGGATCCGGAGCCTTTGTGAATATTCTATCCAG
GTTACTGGATAAGTTGTGATATTGGAAGAGAAACTTTTCCGGCAGTTTCTCTTTGATTATTTG
CATGGCTTCTTCCTTTGAAGAAGCATTCAATGCTTCGGATGCCGCATCATTAGCTGTCTGTTG
ACCTCCTCTAGCAGATCTTCCGTCGACTTGAAAATGACCCCATTCGATGTAATCACCGTCTT
TCTCGATGTAGGACTTGACATCAGAGGATGATTTTGCACTCTGTATATTGGCATGGGAGACG
GAAGAGGTTGTTGGGTGTTTGAGGTCGAACACTCTGCAATTCGTGCACTGGAACTTGCCTTC
GAATTGGATAAGAGCATGCAGATGTGGTTCCCCATTCTCGTGAATTTCTCTGCAAATTCTGA
TGTATTTCTTGTTCACCGGTGTTTGTAGGTTCTGAAGTTGTTCAATTGCACTCTCTTTGGTAAT
TGAACATTGTGGGTATGTGAGGAAATAATTCTTTGCGTTTATTTTAAAACGCTTTGGTGGTG
GCATACTTGTAAATAAGAGGGTGTACCCCGATTGAGCTCTCGTTCAAAAGTCTCTATGAATC
GGTGTAATGGTGCCAATATATAGTAAGAAGTTCTTTAAGGATCTGGAGACACGGGAGACAC
GTGGCGGCCATCCGTTATAATATT

>A_GO_143_LR_LVV_08_MN928610 Tomato golden vein virus (TGVV) - DNA A

ACCGGATGGCCGCGCGATTTTTTTAAAGTGGTCCCACTGTGAGTGACCAATCAGATTGTGTC
TTGGACGGTTATTTATTTCGAAATACTTAGGCGCTAAGTTGTTAAAGTTGTATAAATTAGAC
ATTCATAAGTGGTCGACATACTTTAATTCAAAATGCCAAAGCGAGATGCCCCATGGCGTAT
AGTGGCAGGGACCACGAAAGTATCCCGCTCTTCTAATTATTCACCTCGGGGAGGTATACCC
AAGCGGGATGCTTGGGTAAACAGGCCCATGTACAGGAAGCCCAGGATATATCGTACGTTG
AGAGGGCCTGATGTTCCTAAAGGGTGTGAAGGCCCATGTAAAGTTCAATCCTATGAGCAAC
GTCATGACATTTCTCATCTTGGCAAGGTGATGTGTATCTCTGATGTGACACGTGGTAATGGT
ATTACACACCGTGTTGGTAAGCGTTTTTGCGTTAAGTCTGTGTACATATTGGGTAAGATATG
GATGGACGAAAATATTAAACTGAAGAACCATACCAACAGTGTTATTTTTTGGTTAGTGAGG
GATCGTAGACCCTATGGGACACCTATGGATTTTGGTCAGGTGTTTAACATGTTTGATAATGA
GCCTAGTACTGCTACGGTGAAGAACGATCTTCGTGATCGTTATCAAGTCATTCATCGATTCA
ATGCTAAGGTTACGGGTGGACAGTATGCTAGCAACGAGCAAGCTCTTGTTCGGCGTTTCTG
GAAGGTCAATAATCATGTGGTTTATAATCATCAGGAAGCAGGGAAATATGAGAATCATAC
GGAGAACGCTCTGTTATTGTATATGGCATGTACTCATGCCTCTAACCCCGTGTATGCTACTTT
AAAAATTCGGATCTATTTTTATGATTCGATAACAAATTAATAAATTTTAAATTTTATTGAATG
ATTTTCGAGTACAGCATTTACATATGATTTGTCTGTTGCGAAACGAACAGCTCTGATTACAT
TATTAATGGAAATAACGCCTAATTGATTCAGATACAATAAAACGAAATATCTAAATCTAGT

TAAATATGTCATCCCAGAAGCTCTCAGGGATGTCGTCCAGACTTGGAAATTTAGAAATGCC
TTGTGGAGACCCAACGCTTTCCGCAGGTTGTGGTTGAAACGTATCTGCACATGGTATATCCT
GGTCGTTGTATAATAGGGGTCCTCGACGTTGGTTATCTTGAAATAGAGGGGATTTGTAATCT
CCCAAATAAAGACGCCATTCTCTGCTTGAGGCACAGTGATGGGTTCCGCGGTGCGTGAATC
CATTATTTCTACAGTTGATGTGGATGTATATGGAGCAGCCGCAGTTTAGGTCTATGCGTTTA
CGCCTGGTTGTTTTCTGTTTGGCGGCTCTGTGCCGTGGCTTGATAGAGGGGGGAGTTGAGGA
AGATGAATTTAGCATTATGCAGTGTCCAAGCTCTTAGAGATGCATTTTCATGTTTGTCAAGG
AAGTCTTTATAACTAGCCCCCTCTCCTGGATTGCAAAGCACGATTGATGGGATCCCTCCTTT
AATTTGAACCGGCTTTCCGTACTTGCAATTGCTTTGCCAGTCACGCTGGGACCCAATTAGTT
CTTTCCAGTGCTTCATTTTTAGATAATGCGGAGTGACATCATCAATGACGTTATACTCCACTT
CATTTGAATAAACTTTAGAATTAAAATCCAAGTGTCCACTTAAATAATTATGTGGTCCTAGT
GCACGAGCCCACATAGTCTTCCCCGTCCTAGAATCACCCTCAATGATGATACTAATAGGTCT
TTCCGGCCGCGCAGCGGCACCCCTTCCGAAATACTCATCAGCCCATTGCTGCATCTCATCAG
GAACGTGAGTAAAGGAAGATAATTCAAATATAGGGACCCATGGGGCTGGAGCCTTTGCAA
ATATTTTTTGGACATGTGATTGTATTTTATCCAAATGAAGAACATAATCTCTAGGTTGTTCTT
CTTTTAATATATTTAAGGCCTTGGATAGATTTTCTGCGTTTAGAACCTTCGCATAAGTGTCAT
TGGCAGTTTGGCAGCCTCCTCTTGCTGATCTTCCATCGATCTGGAAATTTCCAAAATCAATG
AAGTCTCCGTCTTTTTCCATGTAGGTCTTGACGTCGGACGAGCTTTTAGCTCCCTGAATGTTT
GGATGGAAATGTGCTGATCTTGTTGGGGATACCAAGTCGAAGAATCGTTGATTCTGGCATTT
GAATTTGCCTTCGAATTGAATAAGCACATGGAGATGAGGTTCCCCATCTTCGTGAAATTCTC
TGGTTACACGAATAAATTTCTTGTTTGTTGGTGTTTTAAGTGCCTGTAATTGGGAAAGTGTTT
CCTCTTTGGTTAATGAACAATGAGGGTATGTAAGGAAGAAATTTTTTGCATTTACAGTGAAA
CGCTTTGGTGGTGGCATTGTAGTAAATAAGAAGATGAGACCCGATAGCTCTCCTTCAAACTT
GGCGAAATGAATTGGGGAATGGGTCTCAATATATAGTAGAGTCCATTATAGAATATAATTG
CCACGTGGCGGCCATCCGATATAATATT

>B_GO_143_LR_LVV_08_MN928611 Tomato golden vein virus (TGVV) - DNA B

ACCGGATGGCCGCGCGATTTTTTTTTGGTGGACCCATTCTGAAAGTAATTGAGCGCATTTTT
GACGTCCGCGAATTGAGTTGAGCGCAATATTTGAGTTCCGAAAATATAGTTAGTGGAGTAA
CTTTAATTTGAAATAAATTACAGCTTTTAGGGTAACCAATCAGCTTGCGATTCAGGAGTCTA
TTTATCTATTACATTTATGTCAGTGTCAAGGTATACGGTATTCTTCACGTGGACCAATTAATT
GTTCGCTATGGAGTCTAACTAATTAATATACGAAGTGATGATTTTTTATATAAATTCCATAT
ATATTGTGGTAGATATGATATCGCATCATGTCTCCTACTAGCTATAGACGTGGTTGGTCGTTT
GCTCAACGACGAGGTTATTCTCGTAAATATTTGTTCAAACGTCCTTATTATTTTAAACGTAC
AGATGGGAAACACCGATCGGGTAACTCGAATCAGGTCAATGAGGATATTAAGTTGTCGCA
ACAGCGTATACATGAGAACCAGTTTGGTCCGGAATTTGTAATGGGTCATAATACAGCAATA
TCGACTTTTATCACTTTTCCTAGTCTATGTAAAACTGAACCGGGCAGATCCAGGGCATACAT
TAAGTTGAAACGTTTACGTTTCAAAGGTACCGTCAAGATTGAGCGTGTTCATGCTGATGTGA
CTATGGATGGTATAATACCAAAGATTGAAGGCGTATTTTCTATGGTTGTCGTTGTTGATCGT
AAACCCCATTTGAGTTCATCTGGTTGTCTGCATACATTTGATGAGCTCTTTGGTGCACGGATC
CACAGTCATGGCAATTTGGCTGTAACTCCATCTTTGAAAGAGCGTTTTTACATACGTCACGT
TTGGAAGAAAGTAATATCTGTTGAGAAGGACAGTATGATGGTTGATCTTGAAGGAACGACA
TCGTTGAGTAATAAGCGTTTTAATTGTTGGTCTGCTTTTAAAGATCTTGATCGTGATTCATGT
AATGGTGTTTATGCGAATATAAGCAAGAACGCCCTTTTAGTTTATTACTGTTGGATGTCGGA
TACGATGTCTACGGCGTCATCATTTGTATCGTTCGATCTTGATTATGTTGGTTAAAAATTAAA
ATATTAATGTATTTATACTTACATGAGAAAATAATTCATTTAAGAAAACGTGAGAAAATAA

ATCATTTAAGAAAACGTGAGAAAATAAATCATTTAAGAAAACGTAATCACATAATATAATT
ATAATTACTGAAATATTGGCTGCGCAGCTGCATGCAAAAAAAAATATTGGCAACTTGATAT
TTATTTCAATGATTTGGGTTGTGGAGGAATACAGTTAGATTTAATACATTCTTGGACTGTTGA
CCTAACAAGATCGTTTAATTGGGCCACTGACAGCGTGATGTTGGATTGCGTTCTTTGGGCCC
CAATTATTGAAGCCGAATCACCTGGATCTAATATGGCAGTTCCTAGCCTATTTAGATCTTTG
TATGGATGTAACGCCTCCCCTAGGTCTGATTGGGCTTCTAATTGGGTCGTCTCTATTGTACTC
CTTGTGGCCCATGACTCTCCTGGATTTAGTTCTATTGGGCCATGAAGCCCAAATCTAGAAGT
TGATGCCGATCTTATCAATTTTCTCTCCCACTTTCCATAACCTACATGAGAGAAGTCGATGT
CATTGACGGTAAATTGTTTGGACATTATCTTCACTGTGGGTGCCCTGAAAGGTATATCAACC
GAATGTTTTGCCGTTGATAGCTTCAATTTTCCTTTGAATTTTGCGAAGTGGGTCATCTGATGA
ACGTTTGAGTCAGAAACCTTATAATATAGCTTCCATGGAATTGGGTCTTTGAGTGAGAAAA
ATGATGACGAGAAATAGTGGAGGTCTATGTTGCACCTGATTGGAAAAGTCCATGACGCCTG
TAAGGATTCATTTTCAGTCATCCTCTTGTCATGAATCTCCACAATTACTGATCCAGTTGCGTT
GATTGGTACCTGTTGTCTATATTCAATGACGCAATGATCAATCTTCATACAGCTTCGATTTAG
TCTTGCACTTAATTGAGTTGCTTTTGATGGAAATTGCAATATGATTTCGGTAAGGTCATGAG
ATAGTTGATATTCATCACGACGAGATTCTATATAATTAAAGGCGTTGGGTGGGCAAACTAC
TTGAGAGTCCATATGTATTACAAGATTGAATAAGTGTAGAGAATAATTTAGAAAAAGTAAA
GGAAACAGTAGTAGTGATAGATAAGATGAGTGAATGGATGTGATATTATACAGAATTGTCG
ATCCTTATATAGACTCTTTACTGGTTATGGATAATTTGGTTAAACAATACTGAGTATACTTAC
TAAATAAGTTTATAAAGTTACAGTGAAACGCTTTGGTGGTGGCATTGTAGTAAATAAGAAG
ATGAGACCCGATAGCTCTCCTTCAAACTTGGCGAAATGAATTGGGGAATGGGTCTCAATAT
ATAGTAGAGTCCATTATAGAATATAATTGCCACGTGGCGGCCATCCGATATAATATT

Sanger sequences

>DF_677-DNA-B-ToRMV-DNA-B-F_E06.ab1

TGTAGATGAAATTTAGACAGTCCACGTCGTAAATTTCTTGTATTTTTAACGAACGGTTGTAA
TTACACCACGACAGGAAATAAACCAGCGAAATGATTGATCAATATGAACTATCCAACCTTT
AATTCAAATTAAAGGTGATTCACAATAGCGGATTTCAAAAAGACAATTAACTCATTTCGCG
GACTTCAAAAAAGCGCTCAATTAAAATTGAAAGCGCGAGGCAACGTACACGATTTCGCCA
CGTAGCACATAAGAAAAATCGCGCGGCCATCCGGTAATATTATAACGGATGGCCGCCACG
TGTGGCAATCTCCTTGAAAAGAACTTCTACTATATAAGAGCTACCATAACTACCAATTCATA
TGAGTTTTAGAGCAGCTACCATTTGGTAGTAAGGGTCATATTTACAAATATGCCCTTGCCTT
GAAAATTCTATTTAAAGAGCAAGTAGAGGGGTATTTATCAATTACACCTAAAATAGACAAT
ATCTCATCTCAATTCGTCGTCTCCTGATTCCTCGTTTTTTACTTATTATCATCTCCAATTTCCC
AATTGGATCAACAGACAATTTTCTTGTCATATATTCAGTTGACGAAAATATTATCCTCTTCCC
TTATTAACTTTCCTCAAACATTGTCGCTGCGCTCCCTATTTTCAATAATGGAATCTCAGCTTG
TGAATCCTCCCAACGCATTTAATTATATAGAATCTAAGCGTGATGAATATCAGCTTTCCCAT
GATCTAACCGAGATAATGCTTCAATTCCCTTCAACGGCGTCTCAATTAAGCGCAAGGCTTA
GTCGTAGCTGTATGAAATCGACCATTGCGTAATAGA

>DF_566-A-ToSRV-DNA-A-R_H11.ab1

GCTGCCTGAAAAGTTCTTATTTCAGTTTCACAATCTAAATAGTAATTTAGATAGGATATTTA
CAAGGGCTCCGGAGCCATGGGCCCCTACGTTTCCCCTCACCTCTTTCACTAGCGTACCGAGA
GAGATGCAAGCTTGGGCAGATGACTATTTTGGGAGAGGTGCCGCTGCGCGGCCGGAACGA
CCTATTAGTATCATCATTGAAGGTGACTCTCGAACGGGAAAGACGATGTGGGCACGTGCAT

TAGGGGCCCATAATTATTTGAGTGGACACCTAGATTTCAATCCTAGGGTTTATTCAAATCAT
GTTGAATATAACGTCATTGATGACATCGCACCGCATTATCTAAAGTTAAAGCACTGGAAAG
AATTGCTTGGGGCCCAAAAGGATTGGCAATCAAACTGCAAATACGGAAAGCCAGTTCAAA
TTAAAGGTGGTATCCCGTGCATCGTGCTTTGCAATCCTGGCGAGGGGGCCAGCTATAAATG
TTTCCTCGACAAAGAAGAAAATGCAAGTCTAAATAATTGGACAAAGCACAATGCGAAATT
CGTCTTCCTTAACACCCCCCTCTATCAAGGTACAACACAGAGCTGCTAAAAAACGAGGAAT
CAGGAGACGACGAATTGATATAGAGTGCGGGTGCTCTATTTACGTACACATCGACTGCAGA
GGACATGGATTCACGCACAGGGGAACTCATCACTGCACGTCAGGCAGAGAATGGCGTCTA
TATTTGGGAGATATCAAATCCCCTCTATTTCAAGATAAACAGAGTGGAGGATCCAATGTAC
ACCGCGAGCAGAGTGTACCACGTCCAAATACGGTTCAACCAC

>GO_118-DNA-A-ToRMV-DNA-A-F_C06.ab1

TTTAGAACTACTTAACGCCCAAGTTGCATAACGGCTATAAATTAAACGTGCATATGTCGAT
AGCTTTAATTCAAAATGCCTAAGCGCGATGCCCCATGGCGCCACATGGCTAGTACATCTAA
GATTAGCCGGGGCGTTAATAATTCTCCACGTGCAGGAGTTGGGCCTCGGTCCAACAAGGCC
ACTGACTGGGTAAACAGGCCCATGTACAGGAAGCCCAAGATATATCGGATGTATAGAACC
CCCGATGTTCCAAGGGGTTGTGAAGGCCCATGTAAAGTGCAGTCGTTTGAACAGCGTCACG
ATATCTCCCATACTGGTAAGGTGATGTGCGTCTCCGATGTGACACGTGGTAACGGTATTACA
CACCGTGTGGGTAAGCGTTTCTGTGTTAAGTCTGTGTATATTCTGGGTAAGATATGGATGGA
TGAGAACATCAAGTTGAAGAACCACACGAACAGTGCCATGTTCTGGTTGGTCAGGGACCGT
AGACCTTATGGTACCCCTATGGACTTTGGCCAGGTGTTTAACATGTTCGACAACGAGCCTAG
CACCGCCACTGTGAAAAACGATCTCCGTGATCGTTTTCAAGTCATGCATAAGTTCTATGCTA
AGGTCACAGGTGGACAATACGCAAGCAACGAGCAGGCGCTTGTCAAGCGGTTCTGGAAGG
TCAACAATCATGTCGTCTACAACCATCAAGAAGCTGGAAAATACGAGAATCATACGGAGA
ACGCCCTATTATTGTATATGGCATGTACTCATGCCTCAAACCCCGTGTACGCAACTTTAAAA
ATTCGGATCTATTTTTATGATTCGATAACAAATTA

>A DF_529 ToMoLCV DNA A C06.ab1

ACCGGATGGCCGCGCGGGTTTTTTTTGACCCGCTCCGTGATGTATTTTTTGTCTTTTACTATGT
GGTCCAGTCAATAAATGACAAATATGACCGTCCAATCAGAAATGGTCCTCAAAGCCTAATT
ATTTAAAAATACTTGGTCACTAAGTTTGGTAAAGTTTATAAATGGTCCTTCCTCGTAATGTTA
TACCAACTTTAAGTCATAATGCCTAAGCGTGATGCCCCATGGCGCTCAATGGCGGGGACCT
CAAAGGTTAGTCGGTCCGCCAATTTCTCCCCTCGTGGAGGAATCGGGTCGAAATCAGAGAA
GGCCCAGGCCTGGGTTAACAGGCCCATGTTCAGGAAGCCCAGGATATATCGGATGTATAGA
ACCCCCGATGTACCAAAGGGATGCGAAGGCCCATGTAAGGTCCAATCCTTCGAGCAGCGT
CACGATGTCTCTCATACGGGGAAGGTGATGTGTATTTCCGATGTCACTCGTGGTAACGGTAT
TACCCACCGCGTGGGTAAACGTTTTTGTGTCAAGTCTGTGTATATATTAGGCAAGGTCTGGA
TGGACGACAACATCAAGTTGAAGAACCACACGAATAGTGTGATGTTCTGGTTAGTTAGGGA
CAGGAGACCGTATGGCACTCCCATGGATTTTGGCCAGGTGTTCAACATGTTTGACAACGAG
CCCAGTACTGCCACGGTGAAGAACGATCTCCGCGATCGTTACCAGGTCCTGCACCGGTTCT
ATGCCAAGGTTACAGGTGGACAATACGCCAGCAACGAGCAGGCTATTGTCAAGAGGTTCT
GGAAGGTCAACAACCATGTGGTGTACAACCATCAGGAGGCTGGGAAATACGAGAATCATA
CGGAGAACGCTCTCTTATTGTATATGGCATGTACTCATGCCTCTAACCCCGTGTATGCTACTT
TAAAAATTCGGATCTATTTTTATGATTCGATAACAAATTAATAAAGTTTAAATTTTATTGAAT
GATTTTCGAGTACATAATTTACATATGGTT

>GO_143-B-ToGVV-DNA-B-R_G11.ab1

GCTTCATGGCCCATAGAACTAAATCCAGGAGAGTCATGGGCCACAAGGAGTACAATAGAG
ACGACCCAATTAGAAGCCCAATCAGACATAGGGGAGGCGTTACATCCATACAAAGATCTA
AATAGGCTAGGAACTGCCATATTAGATCCAGGTGATTCGGCTTCAATAATTGGGGCCCAAA
GAACACAATCCAACATCACGCTGTCAATGGCCCAATTAAACGATCTTGTTAGGTCAACAGT
CCAAGAATGTATTAAATCTAACTGTATTCCTCCACAACCCAAATCATTGAAATAAATATCA
AGTTGCCAATATTTTTTTTTGCATGCAGCTGCGCAGCCAATATTTCAGTAATTATAATTATAT
TATGTGATTACGTTTTCTTAAATGATTTATTTTATCACGTTTTCTTAAATGTATTATTTTCTCAC
GTTTTCTTAAATGAATTATTTTCTCATGTAAGTATAAATACATTAATATATTAATTTTTAACC
AATATAATCAAGATCGAACGATACAAATGATGACGCCGTAGACATCGTATCCGACATCCA
ACAGTAATAAACTAAAAGGGCGTTCTTGCTTATATTCGCATAAACACCATTACATGAATCA
CGGTCGAGATCTTTAAAAGCAGACCAACAATTAAAACGCTTATTGCTCAATGATGTCGTTC
CTTCAATATCAACCATCATACTGTCCTTCTCAACAGATATTACTTTCTTCCAAACGTGACGTA
TGTAAAAACGCTCTTTCAAAGATGGAGTTACAGCCAAATTGCCATGACTGGTGGATCCGTG
CACCAAAGAGCTCATCAAAT

>GO_143-A-ToGVV-DNA-A-R_F11.ab1

AGTATTTCGGAGGGGTGCCGCTGCGCGGCCGGAAAGACCTATTAGTATCATCATTGAGGGT
GATTCTAGGACGGGGAAGACTATGTGGGCTCGTGCACTAGGACCACATAATTATTTAAGTG
GACACTTGGATTTTAATTCTAAAGTTTATTCAAATGAAGTGGAGTATAACGTCATTGATGAT
GTCACTCCGCATTATCTAAAAATGAAGCACTGGAAAGAACTAATTGGGTCCCAGCGTGACT
GGCAAAGCAATTGCAAGTACGGAAAGCCGGTTCAAATTAAAGGAGGGATCCCATCAATCG
TGCTTTGCAATCCAGGAGAGGGGGCTAGTTATAAAGACTTCCTTGACAAACATGAAAATGC
ATCTCTAAGAGCTTGGACACAGCATAATGCTAAATTCATCTTCCTCAACTCCCCCCTCTATC
AAGCCACGGCACAGAGCCGCCAAACAGAAAACAACCAGGCGTAAACGCATAGACCTAAA
CTGCGGCTGCTCCATATACATCCACATCAACTGTAGAAATAATGGATTCACGCACCGCGGA
ACCCATCACTGTGCCTCAAGCAGAGAATGGCGTCTTTATTTGGGAGATTACAAATCCCCTCT
ATTTCAAGATAACCAACGTCGAGGACCCCTATTATACAACGACCAGGATATACCATGTGCA
GATACGTTTCAACCACAACCTGCGGAAAGCGTTGGGTCTCCACAAGGCATTTCTAAATTTC
CAAGTCTGGACGACATCCCTGAGAGCTTCTGGGATGACATATTTAACTAGATTTAGATATTT
CGTTTTATTGTATCTGAA

>DF_640 New species#1 - DNA A-F_A06.ab1

GCGCTCTATGGCGGGTATCTCAAAGGTTAGCCGCTCACTTAATTTCTCGCCTCGTGGAGGTA
TTAATCCCAAATTTGATAGGGCCTCAGCGTGGGTCAATCGACCCATGTACAGGAAGCCCAG
GATATATCGGACTATTCGAAGCCCTGATGTCCCTAAAGGGTGTGAGGGGCCTTGTAAGGTC
CAATCATTTGAACAGCGCCACGATATCTCCCATACCGGGAAGGTGCTGTGTATATCTGATGT
GACACGGGGCAGTGGTATTACCCACCGTGTCGGTAAGCGTTTTTGCGTGAAGTCCGTTTATA
TTTTAGGCAAGGTATGGATGGACGAGAACATCAAGTTGAAGAACCATACGAACAGCGTCA
TGTTCTGGTTGGTTAGAGACAGGAGACCCTATGGCACGCCGATGGATTTTGGACAGGTGTTC
AACCTGTTTGATAATGAGCCCAGTACTGCGACTGTGAAGAACGATCTCCGGGATCGTTTCC
AAGTGATGCACAGATTCCACACTAAGGTGACGGGTGGACAGTATGCGAGCAACGAGCAGG
CTCTTGTCCGGCGATTTTGGAAGGTCAACACCCATGTTGTGTACAACCATCAGGAAGCTGC
GAAGTATGAGAACCACACGGAGAACGCCCTCTTATTGTATATGGCATGTACTCATGCATCT
AACCCTGTGTATGCAACCCTTAAGATCCGGATCTATTTCTACGATTCGGTATCGAATTAATA

AAGTTTGAATTTTATTGAATGATCTTCGAGTACAGATGTTACATATGGTCTGTGTGTTGCGA
AACGAACAGCTCTAATTACATTATTAATGG

>DF_640 New species#1 - DNA B-F_B06.ab1

TTGGTTGATTATTAAGTTAACCGTGTGTATTTGCATAAAATATAGACAAGTGTGTACTGATT
GTTATATTATAAATCCAAAAAATAATTATAAATAAGATATGGTCTTGCAAAATAAATAATT
GTAATAACTTTATATTTATTTTAATGACTTGGGTTGTTGTGGAGTACAATTTGTGTTAATGCA
CTCTTGGACCGTTGTCCTCACAATATCGTTTAGTTGGGCCAACGACATTGTTATGTTGGATTG
TGTCCTTTGAGCCCCGACTATTGAAGCTGATTCACCTGGGTCTAGCATGGCGGTCCCTAGTC
TGTTGAGTTCTCGATACGGATGTATTGTGTCACCTATGTCGGACTCCGCATTTTCATGGCTGT
ATCCTATCGTGCTTCTGGTAGCCCATGACTCTCCGGGCTTTAGTTCAATTGGGCCGGGTAGC
CCAAATCTAGACGTGGAAGCGGATCGTATGAGCTTTCTCTCCCATTTTCCGTAGCCCACATG
ACAGAAATCTATGTCTTTTTCTGTGAACTGCTTAGACAAGATTTTAACCGTCGGAGCTCGGA
ATGGGATATCAACTGAATGTTTAGCAGTGGACAGTTTGAGTTTGCCTTTAAATTTGGCGAAA
TGTGTGCTCTGATGTACGTTGGTGTCGCAAACTCTGTAATATAGCTTCCATGGAATTGGGTC
CTTTAGGGAAAAGAAGGAAGAGGAGAAATAATGTAGATCTATGTTACACCTGATCGGAAA
TGTCCATGACGCTTGTAAGGATTCGTTGTCTGTCATCCTTTTGTCATGAATCTCGACAATTAC
CGAACCTGTAGCGTTGATA

>MG_388-ToCmMV-R_E11.ab1

TATTCAGATTAACGGCGAGAAACATATTTCTAACATATCCACAGTGCGATATACCAAAAGA
TGAAGCTCTTCAAATGCTTCAATGTCTTCCGTGGTCAGTCGTCAAACCCATATACATACGCG
TCGCCAGAGAGGAACACTCCGACGGGTTCCCCCATCTTCACTGTCTTGTCCAACTCTCCGGA
AAATCGAATATCAAGGATGTTAGGTTTTTCGACCTCACTCACCCCAGACGGTCTGCCGGATT
TCACCCAAATGTTCAGGCAGCCAAAGACACCAATGCAGTCAAGAATTACATCACCAAAGA
AGGTGATTATTGTGAATCCGGACAGTACAAGGTGTCTGGGGGAACAAAGGCAAATAAAGA
CGACGTGTACCACAACGCCGTCAATGCACCTTCTCCGGGAGAGGCTCTCGACATTATCAGG
GCCGGAGACCCCAAGACTTTCCTCGTCAACTATCACAATGTGAAGGCTAATCTAGAGAGGA
TTTTCCAAAAGGCTCCGGCACCGTGGGTTCCTCCGTTTCCCCTCTCCTCATTCACTAACGTGC
CCGACGAGATGCAAGAATGGGCTGATGATTATTTGGAAGGGATGCCGCTGCGCGGCCTAT
CAGACCTATCAGTATTGTCATCGAGGGTAATAGTCGGACGGGGAAGACGATGTGGGCGCGT
GCACTTGGCCCACATAATTATCTAAGTGGACACCTGGATTTCAATTCTAGGGTTTACTCAAA
CGATGTGCTGTATAACGTCATTGATGACGTCTCTCCGCATTACTTAAAGTTAAAGCACTGGA
AAGAATTGA

>MG_378 New species#2_B11.ab1

CCTGGGAGGTGCACAATCCCCTTTATTTCAAGATAACAAACGTGGAGGTTTTACTCCACGC
AAACACAAGAGTGTACCACATCCAAATACGGTTCAACCACAACCTGAGGAGAGCGTTGGA
TCTCCACAAGGCATACCTGAACTTCCAAGTCTGGACGACTTCACTGACAGCTTCTGGGACG
ACTTACTTAAATAGATTTAAATTCTTGGTCAATCTGTACTTAGATCAATTAGGAGTTATTTGC
ATTAACAATGTAATTAGAGCTGTTCGCTTCGCAACAGACAAACCGTATGTAAATCATGTAC
TCGAAGACCATTCAATAAAATTTAATATTTATTAATTTAAGACCGAATCATAAAAATAGAT
CCGAATCTTAAGGGTAGCATACACAGGGTTGGAGGCATGCGTACATGCCATATACAATAAC
AGGGCGTTCTCCGTGTGATTCTCGTACTTCGCGGTCTCTTGGTGGTTGTAGACCACGTGGTTG
TAGACCTTCCAAAAACGCCGCACAATAGCCTGCTCGTTACTGGCATACTGACCACCCGTGA

CCTTGGCGGAGAACTTGTGCAAAACCTGAAAACGATCACGGAGATCGTTCTTCACAGTAGC
GGTACTGGGCTCATTATCATACAGATTGAAAACCTGGCCGAAGTCCATAGGGTTACCATAC
GGCCTCCTATCCCTAACTAACCAAAACAATACGCTATTCGTATGGTTCTTCAACTTAATATT
ATCGTCCATCCACACCTTCCCTAATATATACACAGACTTAACACAAAAACGCTTACCGACA
CGGTGAGTATTACCATTACCCC

>MG_150-DNA-B-ToCMoV-DNA-B-R_G12.ab1

TATTTACTGTATAACCAAGATAACCCATTGATGCAGTCACAGCTATCTATATAACGGATTGA
TATGGATAATTATAAAACGTCCAGTAACAATTTTGAACATGTATTTTAATACTAATAAGCGT
CGTTGGTTATCCACCAACCGTCGTAATTACGTGCGATATCCTACGTTTAAAAAGTCACATTC
TGTGGTACGCAATGATGGGAAACGTCGACTGGGTATTTCAAACAAGCCCCACGACGATAGT
AAGATGTCTTCGCATACGATTCATGAAAACCAATATGGGCCTGACTTTGTATTGGGCCATA
ATACAGCGATATCCACGTTTATAACTTACCCCTCTCTTGGTAAAAGTGAGCCCAATCGTATT
AAGTCATACATTAAGCTGAAACGTCTGCGTTTTAAAGGTACTGTTAAGATTGAACGTGCTCC
CAACGATTTGAACATGAATGGATTACCTGCTAAGATTGAAGGAGTATTTTCTTTGGTTATTG
TTGTCGATCGTAAACCTCATTTGAGTCCTTCTGGAAGTCTCTACACATTCGATGAGTTATTTG
GTGCTAGGATCCATAGCCATGGAAATTTAGCGATAATCCCGTCTCTGAAGGATCGTTTTTAC
ATACGTCATGTTGCTAAACGTGTCTTGTCAGTGGAGAAGGATAGTAATATGATTGACGTTGC
TGGGATTACAACACTCTCTAGTAGGCGTTATAATTGCTGGGCAAATTTTAGAGATTTCGATC
ATGAATCCTGTAACGGTGTATATGCTAACATAAGCAAGAACGCCCTACTAGTTTATTATTGT
TGGATGTCGGAAACAGTTTCCAAGCATCTACATTTGTATCATTTGA

>MG_150-DNA-A-ToCMoV-DNA-A-F_F06.ab1

ATATCGGATGGCCGCCACGTGTGTGAACGAGATTGAAAGTTCTATAGAGGGTTCTAGTATA
TATAGGACCCCAGTCCCCAATTGCTTTAGTAAGTTTGAGAAGACCCCAATCGGTGCCCAGC
TATTATTACAAATATGCCACCGCCCAAACATTTCCGAGTAAATTCCAAAAATTATTTCCTGA
CATATCCACACTGTTCTCTTTCCAAAGAAGAAGCACTTTCCCAATTATTAGCCCTAAACACA
CCAACTAATAAATTATTCATTCGTGTCTCGAGAGAACTACACGAAGATGGGCAACCTCATC
TCCACGTGCTCATCCAATTCGAAGGAAAATACAACTGCAAAAATAACAAGTTCTTCGACCT
CGTCTCCCCAAGTAGGTCAGCACATTTCCATCCAAACATACAGGGAGCTAAGAGCTCGTCA
GATGTCAAATCATACGTGGAAAAAGACGGAGACTTCGTTGATCATGGAGTTTTCCAGATCG
ATGGCAGATCAGCTAGAGGAGGTCAGCAATCTGCCAACGACACGTACGCCAAGGTTCTCA
ACGCAGGATCTGTCATGGAAGCACTCAATATATTAAGGGAGGAACAACCCAAAGATTTCG
TGCTTCAACATCATAACATTAGGTCAAATTTAGAGCGTATTTTCGCAAAGGCTCCAGAACCT
TGGGTTCCTCCGTTTCCACTCTCGTCGTTCACTAACGTGCCTGTCGAGATGCAAGATTGGGT
AGATAATTATTTCGGGAGAGATGCAGCTGCGCGGCCGGAGAGACCTATCAGTATAATAATC
GAGGGTGATTCTCGAA

>MG_122-DNA-B-ToRMV-DNA-B-F_D06.ab1

TTATATAGAGGAAATTTAGACAGTCCACGTCGTAAATTTCTTATATTTTTAAAGAACGGTTG
TAATTACACCACGACAAACAATAAACCAGCGAAATGATTGATCAATATGTACTATCCAACC
TTTAATTCAAATTAAAGTTGATTGACGGACAGCGGATTTCAAAAAGACAATTAAATCATTT
CGCGGACTTCAAAAAACCGCTCAATTTAAATATAAAGCGCGAGGCAACGTACACGATTTC
GCCACGTAGCACATAAAAAAATCGCGCGGCCATCCGGTAATATTATAACGGATGGCCGCC
ACGTGTGGCAATCTCCTTAAAAAGAACTTCTACTATATAAGAGCTACCATAACTACCAATT

CATATGAGTTTTAGAGCAGCTACCAATTGGTAGTAAGAGTCATATTTACAAATATGCCATTG
CCTTAAAAATTCTATTTAAAGAGCAAATAGAGGGGCATTTATCATTTACACCTTAAATAAAT
AATATATCCTCTCAATTCGTCGTCTCCTGATTCCTCGTTTTTTAGTTATTATCATCTCCAATTT
CCCAATTGGATCAACAGACATTTTCTTGTCATATATTCAGTTAACGAAAATATTATCCTCTTC
CCTTATTAACTTTCTTCAAACACTGTCGCTGCGCTCCCTATTTTCAATAATGGAATCTCAGCT
TGTGAATCCTCCCAACGCATTTAATTATATAGAATCTAAGCGTGATGAATATCAGCTTTCCC
ATGATCTAACCGAGATTATGCTTCAATTTCCTTCAACGGCGTCTCAATTAAGCGCAAGGCTT
AGTCGTAGCTTTTATGAAAATCGACCATTGCGTAATAGAATACAGACAA

>GO_298-Gemycircularvirus_E12.ab1

ACTCAGCCCACATCTCTAGACGTGGGTCTCTGTTAGCCAACCAGATGCAAGGTATGACTTG
CAAGTTGTACGGGATCTCTGTACAGCTCTTTGACTGTGACCACACTCTGTGCCCCAAGCCAC
TCTTTCCATGAATGGAAATATCCAATACCACCTCGCATATCGTCGAAGACAGCGTACGCAG
CTCCCGGTGCATCACGCAGGAGAAGCTTGCCGGACACGATGCCCATACTGTACACGTGTGG
GCCCAGTGATCTAGCCCAAACAGTCTTTCCAAGTCGTGAAGGACCATAGAGTACAAGAGA
CCGCATTCTGCCTTGCTTAGTCAGCATTGCACACACTCACGTAGCGGTCCGAGCGGCGGAG
CGAGATCGGACCTTGCGGAACCGAGCACTACGCTGGCAAAAACCCCTAGGAGACTATAGA
CCTCCCGAGAGAGCCCCGCGACCCAGAGGGGAGCGTAGGGCGATTGAGACGTACCTTCTA
CTGGATCATTTGCAACAAGCTGTTCTCCCCATCGAGCCAGCTCAGGTACCATTCCAAGCTCA
AATTGCTTCCCATCGGAACCCACATAGGGTTCTGGGGTGGGGGCGTAGGCGGCGTCGGCAT
ACTTGTTGAGCTCGGTCCAACGAGTGACAAGTGTCTTCGGATCCAGCTGTCGAAGCAGGTC
AAAAAACTCGTCTCGACTTTCTGCCGAGACAATCTCGCGCCATTTATCCGCAACTTCAGGTA
ATCCACCTCCGCCCGGCCTCGCCAACCCCCCTGCAACAACGTTTCCATCTTTGACTGCATAG
TCGTAACCGAGCTCCGGACGACCTCGAGATGGCGTAAT

>GO_169 New species#3_D05.ab1

CCGCGCGATTTTTGGAGTCCTATGCGCTCTGGCCCAGCCCATTTGAATTAAAGATCGATATC
TTTTCGCTCGACCAATGATATCGCTTCTGGGAAGCCTAGATATTTGTTCCGATACTTACGGC
CGAAGTTCTTGATTAACGGCTATAAATTAAAGGAAGACTGGTCACAGTCTTTAATTCATAAT
GCCTAAGCGGGACGCCCCGTGGCGCCTGATGGCGGGAACTTCGAAGGTTAGCCGCTCTACG
AACAGTGCCCCTCGTGGAGGGTTTGGGCCTAAGATCAACAAGGCCTCTGAATGGGTTAACA
GGCCAATGTATAGGAAGCCCAGGATATACCGGGCTTTCAGAACTCCCGACGTTCCTCGAGG
GTGTGAAGGGCCTTGCAAGGTGCAGTCGTACGAGCAGCGACACGACGTCTCACATGTCGGC
AAGGTGATGTGCATATCCGATGTGACACGTGGTAACGGCATTACCCACCGTGTCGGTAAGC
GTTTTTGTGTTAAGTCTGTGTACATTTTAGGTAAGATATGGATGGATGAGAATATCAAGCTC
AAGAATCACACGAATAGTGCGATGTTCTGGTTGGTCAGAGATCGTAGACCGTATGGTACCC
CAATGGATTTTGGGCAGGTGTTCAACATGTTCGACAACGAGCCCAGCACTGCCACGGTTAA
GAACGATCTCCGCGATCGTTACCAGGTCTTGCATAAGTTCTATGCCAAGGTCACCGGAGGA
CAGTATGCCAGCAATGAACAGGCCCTAGTCAAGAGGTTCTGGAAGGTCAACAATCATGTG
G

>DF-46_BGMV DNA A

ACGGATTGCCGGCGAATTTTTTACTGGGCCATCATGGCCGTGCCGTCTATGATAGTAAAGTC
ATTATTCGACATTACTAGTCGACGCTTAGTATTTGACTTGCGAACAAGTTCTTACTAACTAT
ATTATCTTGTATGCATGTTCTTATCAAATGCTAGCGTGATGCTCATGCGCCACCATGCAGTA

CGTCAAAATAGCGTCGCTATTCTCTCCTCGTGGGGAAATGGGCCTAAATACAACAAGCCG
CTGAATGGGTTAACAGGCCTATGTATAGGAAGCCCAGGATATATCGAACTATACGAGGCCC
TGATGTTCCACGGGGCTGTGAAGGCCCGTGCAAGGTTCAGTCTTATGAACAACGACATGAT
GTGTCACATGTTGGGAAGGTTATGTGTGTATCTGATGTAACAAGAGGTAATGGTATTACCCA
TCGTGTAGGAAAACGTTTTTGTGTTAAGTCTGTTTATATTTTAGGTAAGATATGGATGGACG
AGAATATCAAGTTGAAGAATCACACGAACAGTGTGATGTTTTGGTTAGTCAGAGATCGTAG
ACCGTATGGTACTCCTATGGACTTTGGTCAAGTTTTTAATATGTTCGACAATGAACCCAGTA
CTGCTACTGTTAAGAACGATCTTCGTGATCGTTTTCAAGTTATGCATAAATTCTATGGAAAG
GTGACAGGTGGACAATATGCTAGCAACGAACAGGCATTGGTCAAACGTTTTTGGAAGGTG
AATAATTATGTAGTCTACAACCATCAAGAAGCAGGGAAGTACGAGAATCATACAGAGAAC
GCATTATTGTTGTATATGGCATGTACTCATGCCTCAAATCCAGTGTATGCTACTCTAAAAATT
CGGATMTATTTTTATGATTCGATCACCAATTAATAAAATTTATATTTTATTTCATGATTCTCA
AGTACATAATTTACATATGATTTGTCTGTTGCGAAACGTACAGCTCTGATAACATTGTTAAG
CGAAATAACACCTATTCTATCTAAATACAACAGCACTAAATATTTAAATCTATTTAAATATG
TCGTCCCAGAAGCTTGAAGAGATATCGTCCAGACTTGGAAATTCAGGAAAGCCTTGTGTAG
ATCCAGTTTGTTCCTGAGGTTGTGGTTGAACCGTATTTGGAGGTGGTATACTCTGGTCGTTGT
GTATTGTAGATCCTCTACGTTGTATATCTTGAAATAAAGGGGATTTGATATCTCCCAGATAT
ACACGCCATTCTCCNGCTTGACGTGCAGTGATGCGTTCCCCTGTGCGTGAATCCATGACCGT
AGCAATTTATGTGTACATAAATTGTGCAGCCGCACTCTAAATCAACGCGACGTCGTCTGATT
GCTCGTTTCTTCGCAGCTCTATGTTGAACTTTGATAGAGGGGGGAGTCGAGGAAGATGAATT
TCGCATTATGTATTGTCCAGTTTTTGAGAGCTGCGTTTTCCTCTTTGTCGAGGAAACATTTAT
AACTCGCTCCTTCACCTGGATTGCAAAGCACGATTGATGGGATCCCACCTTTAATTTGAACG
GGTTTCCCGTATTTGCAGTTTGATTGCCAGTCCTTTTGAGCTCCCATCAATTCCTTCCAGTGC
TTTAACTTTAGATAGTGCGGTGCGATGTCATCAATGACGTTATACTCAGCATGATTTGAAAA
CACCTTTGAGTTGAAATCCAGGTGACCACTTAGATAATTGTGGGTCCCTAATGCACGTGCCC
ACATTGTCTTTCCCGTTCGTGAATCACCTTCGACGATGATACTAATAGGTCTCTCCGGCCGC
GCAGCGGCACCTCTCCCGAAATAATCATCTGCCCATTCTTGCATCTCTCTTGGCACGTTAGT
GAAAGAGGAGAGGTGAAACGGAGGTGACCACGGATCCGGAGCCTTTGTGAATATTCTATC
CAGGTTACTGGATAAGTTGTGATATTGGAAGAGAAACTTTTCCGGCAGTTTCTCTTTGATTA
TTTGCATGGCTTCTTCCTTTNGAAGAAGCATTCAATGCTTCGGATGCCGCATCATTAGCTGTC
TGTTGACCTCCTCTAGCAGATCTTCCGTCGACTTGAAAATGACCCCATTCGATGTAATCACC
GTCTTTCTCGATGTAGGACTTGACATCAGAGGATGATTTTGCACTCTGTATATTGGCATGGG
AGACGGAAGAGGTTGTTGGGTGTTTGAGGTCGAACACTCTGCAATTCGTGCACTGGAACTT
GCCTTCGAATTGGATAAGAGCATGCAGATGTGGTTCCCCATTCTCGTGAATTTCTCTGCAAA
TTCTGATGTATTTCTTGTTCACCGGTGTTTGTAGGTTCTGAGTTGTTCAATTGCACTC

>DF23-Alphasatellite

ACCCCGCTTCGCCACAACTCTTCGCCAAAGGGACCCACTCTAGCAATATAAATATGACTAT
TTTGTGCCTATTGCCTTCATAATCCAAATGACACAAAGTCGAAATTGGTGCTTTACTCTCTTT
GCATACACCCTCCCTCTCTTTGACTCTCTCCCCGAGTGGGCTAATTATATAGTTTTCCAGGAA
GAGGAGTGTCCTACGACCAAGAAGAGACACCTACAGGGGTTCGTTACTCTCAAGAAGAGT
CAAAGGCTATCCTTTCTGAAAGGCAGATTAGGTAATGGCGTCCATCTCGAGATCTCTCGGG
GATCCTCATCCAGCAATAGAGATTATTGCAGAAAGGATGATTCAAGAGTCGCTGGGCCATG
GGAATTCGGTGTACTTGCAGAGCAGGGGAGCAATAAGAGGAAGACGATGGAACGTTTTCA
GGAAGACCCAGAGGAGGTCCGACTGGCCGACCCTAAGTTATATCGTCGCTGCCTGGCGAC
AAAGATTAATAAGGAGTTCCGTGGTCTGGTACTCCCTGCATTTGACAGACCTTGGCAGCTGC

TGGTTGAGAAGATATTGGACAAGGGCCCAGATGACAGAACTATCTTATGGGTGTATGGCTC
TCAAGGCAATGAAGGCAAGACGACCTGGGCCAAGTCTAAGATTCAGGCCGGCTGGTTCTA
CTCCAGAGGAGGAAAGGGAGAGAACATCAAGTACTCGTACGCCGAGCACCTAGGTCATGT
CATATTTGATCTGCCCAGACAGGTTGAAGATGTGCTCCAATACTCGGTTCTGGAGGAAATTA
AGGACAGGTTAATTAGGAGTGGTAAATACGAGCCTATAGATTTTAATTGTAGTGACAAGGT
TCACATTGTCGTTTTAGCTAATTTCCTCCCATCGTTAGATGTTGAGCACGATAATAAGGGAA
ATGTAATTAAGCGCCAACTGTTAAGCAGAGACAGGGTATGTATAGTTAACATTGACGAGTC
TGTTGTTGTACGAGAAGAAGAAACGGTGACGTTTGCTCGTTATTTTGAATAAAAGAAAAAA
ACTTGTTTTTGTACAGAAAAAAAAGGACTCCGCCTGGTGTCCTGGCTTATATTTACAAATT
TGCCATCACCCACTAACAACTATGAAAAGAAGGGACCGCAGGGACCACAACCAAAAAAA
AAGAAAAGAAAACCACTAACCAAACAGGGATCGATGACGTGGACAACAACAGTTTTTGTT
ACCGGTGACGTGTCAGTCATATGTGTTAGTTACCGGTTCTATTGCCTTGTAAGGTGACCGGC
TACCGTGGCGAGCTACCGGTAAGAAGCGGGTATAGTATT

>DF_166 SiMMV - DNA A-F_C02.ab1

ACCGGATGGCCGCGCGATTTTTCCCCCCCCCCACGTGGCGCGCTGGTGGTCGTGCGATCTCT
CTCCCCCCCTCTCGCGCGACGTGGAGCTCTGGTGTCCGCGGCGTGTCCCCGCCGCGCGCTCT
GCCTTTAATTTAAATTAAAGGAAATAACTTTCATCAGGACCAATGAAATTGCGTCTTTTAAG
CCTAGATATCAGCGTAAGACTTGGGCCCTAAGTTGTTTGACGGCTATATAATTAAGTCATGT
ATGACGTCAGGAATTATTTCGAAATGCCCAAGCGGGATCCCTCATGGCGCCAGATGGCGGG
AACCTCAAAGGTTAGCCGCTCTACCAACTTTTCCCCTCGTGGAGGTGGAGGCCCAAAGTAC
AACAAGGCCTCAGAATGGGTTCACAGGCCCATGTATAGGAAGCCCAGGATATACAGGATG
ATAAGGACTCCTGATGTTCCAAGAGGCTGTGAAGGGCCCTGTAAGGTCCAGTCCTACGAGC
AGCGTCACGACATCTCACATGTCGGGAAGGTCATGTGCATATCTGATGTCACACGTGGCAA
CGGTATTACCCACCGTGTGGGTAAACGTTTTTGTGTCAAGTCTGTGTATATATTAGGCAAGG
TCTGGATGGATGACAACATCAAGTTGAAGAACCACACGAATAGTGTGATGTTCTGGTTAGT
TAGGGACAGGAGACCGTATGGCACTCCCATGGATTTTGGCCAGGTGTTCAACATGTTTGAC
AACGAGCCTAGCACCGCCACTGTGAAAAACGATCTCCGTGATCGTTTTCAAGTCATGCATA
AGTTCTATGCTAAGGTCACAGGTGGACAATACGCAAGCAACGAGCAGGCGCTTGTCAAGC
GGTTCTGGAAGGTCAACAATCATGTCGTCTACAACCATCAAGAAGCTGGAAAATACGAGA
ATCATACGGAGAACGCCCTATTATTGTATATGGCATGTACTCATGCCTCAAACCCCGTGTAT
GCAACTTTAAAAATTCGGATCTATTTTTATGATTCGATAACAAAT

>MG_016 EuYMV - DNA A-F_C03.ab1

ATCTGGTCTCGTAAGGTGCCGGCTGGTTATCCGAATACGTTCCCCGTGAATTATCATAATGT
CAAAGCTAACGTCGAGCGCCTCTTTATTAAGCCACCCCTTCCTTGGGTTCCCCCATTTCAGC
TGTCGTCATTTAACAATGTCCCGTCTGATTTGCAAGAGTGGGCTGATGATTATTTTGGAAGG
GGTGCCGCTGCGCGGCCAGATAGACCTATTAGTATCGTAGTCGAGGGGGATAGTCGAACG
GGTAAGACCATGTGGGCTAGAGCTTTAGGCCCACATAATTATCTTAGTGGGCATCTCGATTT
CAATTCTAGGGTTTACTCAAATGACGTGGAGTACAACGTCATTGATGATGTCGCTCCGCATT
ATCTAAAGTTAAAGCACTGGAAAGAGTTGATTGGGGCCCAAAGGGACTGGCAGTCCAACT
GTAAATATGGAAAGCCGGTTCAAATTAAAGGCGGGATACCATCAATCGTGCTGTGCAATCC
AGGCGAGGGGGCTAGCTATAAAGACTTCCTCAACAAAGAGGAGAATGCATCGTTGAGAGC
TTGGACACGCAAGAATGCTAAATTCATCTTCCTCCACGCCCCCTCTATCAAAGCACAGCAC
CGAATAGCC

>MG_016 EuYMV - DNA B-F_A03.ab1

CCACGTACCCCCTGGTGTCCTAGAGGCTTTTAATATAAATGGGACACCAGGACACCACGAA
AAATAGGTTTTAGAGAGGACTCCCCCTGGTGTCCTGACTTCTATTTACAAATTTGCCATCAG
CCTGTTGCATATCGCATCTGACTGCCTATATATAGACCTTCAATAGCTAACATATAGGCAAA
TTTCCACTTCTTCATCTCAGATTACCAGCCGATTAAATCATAATCTGCACATAGGCTTATCGT
ATCTTCGAAATTTCCCCAAATTGTTATCGCTAACGCATATCGTCCTTCCATAAGGTTGGCAC
CTATCAATTTCCCTCAGTCGTTCAATTTGGGGGAAGGGGTGCCGCTGCGCGGGCAAGTTTCC
ATAATATAATGGAGGGGTCTCAGTTAGTAAATCCGCCGAGCGCTTTCAATTACATAGAGTC
ACATCGTGACGAGTATCAGCTATCGCACGACCTAACTGAGATAGTCTTGCAATTCCCCATC
AACGGCGTCTC

>MG_150 ClLCrV - DNA A-F_B01.ab1

CCTTCGGGACCACGAAGGTTAGTCGCAACGCCAACTACTCCCCTCGTGGAGGCACGGGCTC
AAGACTAAGCAGGGCCACTGCTTGGGTTAATCGGCCCATGTACAGGAAGCCCAGGATATA
TCGGATGTATAGGTCACCCGATGTGCCCAGAGGGTGTGAAGGCCCGTGCAAGGTCCAGTCT
TACGAGCAGCGCCACGACATATCTCATGTCGGCAAGGTGATGTGCGTGTCTGACATTACCC
GCGGTAATGGTATTACCCACCGCGTTGGTAAGCGATTTTGCGTCAAGTCTATGTACATACTA
GGCAAGGTCTGGATGGACGAGAACATCAAGTTGAAGAACCACACGAACACGGTGATGTTG
TGGTTAGTTAGGGACAGGAGACCTTACGGGACGCCCATGGATTTTGGACAGGTTTTTAACCT
GTTTTTTCTATGAGCCAAGCACCGCTACGGTGAAGAACGATCTTCGTGATCGTTTTCAGGTG
CTGAACAGGTTCCACGTGAAGGTCACTGGTGGACAGTACGCGAGCAACGAGCAGCGCTGG
TTAGGCGCTTTTGGAGGGTGAACACCCATGTGACGTATAACCACCAGGAGGCGGCGAAGT
ACGA

>MG_150 ClLCrV - DNA B-F_A04.ab1

TTTTGAAACGTGTATTGTCGGTCGAGAAGGACTCTCTGATGGTGGATATAGAAGGACGAC
CGTCTTGTCTAGCAAGCGGTTTAGCTGTTGGTCATCGTTTAGGGATCTCGAGAGAGATTCAT
GTAATGGAGTCTATGCTAATATTAGCAAGAACTCCCTTTTAGTTTACTACTGCTGGATGTCG
GACGCGATTTCCAAGGCGTCGACATTTGTATCATATGATCTGGACTATGTTGGCTGAATAAA
GTGATTAATGATGTTTATGAAGATAAACATGTGACAACATATAGAGGATAATCTGAATTAC
AGGCACGATAACTATTCTAAAGGCTTGGTATGCGAAGGGACACAGTTTGTATTTATGCATTC
CTGGACCGTCGTCTTTACTATCTCGTTGAGTTGGGCTAGCGACAGCGTTATGTTGGACTGTGT
CCGCTGAGCCCCAAGTACAGAGGCAGACTCTCCTGGGTCCAGGATTGGTGTACCCAATCTG
TCTAGGTGCCTGTATGGATGCAGCGCATCCCTCCGTTCAGAATCCGCATCGGTATGGGCCG
AGTCGATAGCACTTTTGACGGCCCAAGACTCTCCTGGTTGTATTTCGATTGGGCCGCCCAAC
CCATA
